# Complex multicomponent patterns rendered on a 3D DNA-barrel pegboard

**DOI:** 10.1038/s41467-020-18910-x

**Published:** 2020-11-13

**Authors:** Shelley F. J. Wickham, Alexander Auer, Jianghong Min, Nandhini Ponnuswamy, Johannes B. Woehrstein, Florian Schueder, Maximilian T. Strauss, Jörg Schnitzbauer, Bhavik Nathwani, Zhao Zhao, Steven D. Perrault, Jaeseung Hahn, Seungwoo Lee, Maartje M. Bastings, Sarah W. Helmig, Anne Louise Kodal, Peng Yin, Ralf Jungmann, William M. Shih

**Affiliations:** 1 Department of Biological Chemistry and Molecular Pharmacology, Harvard Medical School, Boston, MA 02115 USA; 2 Department of Cancer Biology, Dana-Farber Cancer Institute, Boston, MA 02115 USA; 3 Wyss Institute for Biologically Inspired Engineering, Cambridge, MA 02138 USA; 4 School of Chemistry, The University of Sydney, Sydney, NSW Australia; 5 School of Physics, The University of Sydney, Sydney, NSW Australia; 6 University of Sydney Nanoscience Institute, Sydney, NSW Australia; 7 Faculty of Physics and Center for Nanoscience, Ludwig Maximilian University, Munich, Germany; 8 Max Planck Institute of Biochemistry, Martinsried, Germany; 9 Danish National Research Foundation: Centre for DNA Nanotechnology at Interdisciplinary Nanoscience Center (iNANO), Department of Chemistry, Aarhus University, DK-8000 Aarhus, Denmark; 10 Department of Systems Biology, Harvard University, Boston, MA 02115 USA

**Keywords:** Synthetic biology, DNA nanostructures

## Abstract

DNA origami, in which a long scaffold strand is assembled with a many short staple strands into parallel arrays of double helices, has proven a powerful method for custom nanofabrication. However, currently the design and optimization of custom 3D DNA-origami shapes is a barrier to rapid application to new areas. Here we introduce a modular barrel architecture, and demonstrate hierarchical assembly of a 100 megadalton DNA-origami barrel of ~90 nm diameter and ~250 nm height, that provides a rhombic-lattice canvas of a thousand pixels each, with pitch of ~8 nm, on its inner and outer surfaces. Complex patterns rendered on these surfaces were resolved using up to twelve rounds of Exchange-PAINT super-resolution microscopy. We envision these structures as versatile nanoscale pegboards for applications requiring complex 3D arrangements of matter, which will serve to promote rapid uptake of this technology in diverse fields beyond specialist groups working in DNA nanotechnology.

## Abstract (web summary)

The design and optimisation of 3D DNA-origami can be a barrier to rapid application. Here the authors design barrel structure of stacked 2D double helical rings with complex surface patterns.

## Introduction

Structural DNA nanotechnology, which relies on Watson–Crick base pairing almost exclusively to drive association, has witnessed an exponential rate of increase in maximum demonstrated complexity of designed shapes since its inception 34 years ago^1–5^. Over the last decade, the most prevalent paradigm for achieving greater complexity has been DNA origami, where the key distinguishing feature is the employment of a long scaffold strand that traverses most or all of the entire shape^6–10^. DNA origami has been explored for diverse applications^11^, including tools for biophysics^12–14^ and plasmonics^15–17^, platforms for diagnostics and therapeutics^18–20^, and templates for nanofabrication^21–23^.

In most of these applications, a single shape of DNA origami has been used with high frequency: the 2D rectangle^6^. The generic appeal of the 2D rectangle can be seen largely in its correspondence to a pegboard, in that it displays a regularly spaced, high-density array of positions (200 in total with a pitch of ~5 nm) for hosting other functional components that is convenient for diverse applications. The design of the underlying staple strands at each position is modular: each is a 32mer and most have a central 16mer that nucleates attachment to the scaffold, and flanking 8mers to link to adjacent helices. Furthermore, folding of 2D rectangles is robust and occurs with high yield. Both the modular arrangement of staple strands and the reliability of assembly introduce ease of operation for newcomers and experts alike.

Here we present DNA-origami barrels as 3D analogs to the 2D rectangle. DNA barrels retain the modular arrangement of staple strands and robust, high-yield assembly of 2D rectangles, while additionally offering features that are desirable for many applications: addressability and shape control in 3D, greater rigidity, an inner surface that is shielded from interaction with other large objects, and a range of sizes. The set of barrels with different diameters (30–120 nm) and heights (15–250 nm) form an all-purpose 3D pegboard, which can be easily repurposed for different designs and applications.

## Results

### DNA-origami barrel architecture

**Fig. 1:**
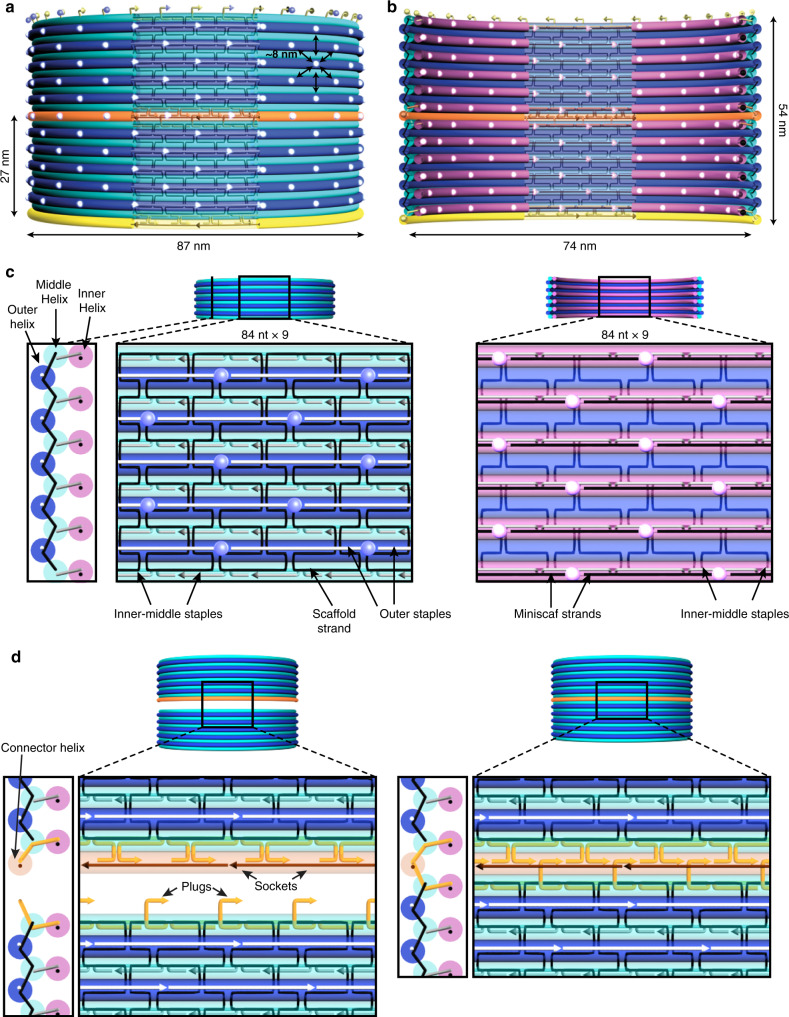
Schematic of coaxially stacked dimer of DNA-origami barrels with ~90-nm outer diameter. Barrels consist of three layers of circular double helices arranged with honeycomb-lattice spacing: **a** measured from helix mid-point, outer helices (dark blue) are 84 nm in diameter, inner-middle helices (cyan) are 81.5 nm in diameter, **b** inner helices (magenta) are 76.5 nm in diameter. Including helix thickness of 2.6 nm gives external barrel diameter of 87 nm and height of 27 nm, with internal cavity of 74 nm. **c** Zoom-in of barrel wall cross-section (left panel), outer surface (middle panel), and inner surface (right panel) for central cut-away region. On the outer surface (left), scaffold routing is rendered as black pipes, and only runs through the outer and middle helix layers. Outer staple strands are rendered as white pipes, and blue fluorescent spheres indicate 3′ ends of outer staples, which form the rhombic-lattice distribution of outside pixel sites. Inner-middle staples are rendered as gray pipes on both surfaces. On the inner surface (right), there is no M13 scaffold DNA. Inner staple strands hybridize to short miniscaf strands, which are rendered as black pipes (left panel). Rhombic-lattice distribution of inside pixel sites, which are 3′ ends of inner miniscaf strands, is rendered as magenta fluorescent spheres. **d** Zoom-in detail of the staple strands that mediate coaxial stacking between monomers, rendered as orange pipes. Staple-strand extensions (plugs) on the top of the interface hybridize to connector strands (dark orange), leaving single-stranded regions (sockets), which plugs from the second barrel hybridize to. In zoom-out view **a**, **b** two orthogonal sets of connector strands are rendered in yellow and orange.

The basic architecture of a DNA-origami barrel is depicted for the 87-nm outer diameter variant (90-nm barrel) in Fig. 1, and for other barrel variants in Supplementary Note 1 and Supplementary Figs. 1–11^24^. One perspective for the architecture of 90-nm barrels is as coaxially stacked DNA duplex rings^25^. Another perspective is a single-layer corrugated sheet, built on a honeycomb lattice, where each double helix is 48 turns long and arranged horizontally; in the view in Fig. 1b, the concave side is facing the viewer. We designate the helices closer to the viewer as middle helices (cyan in Fig. 1) and the helices further from the viewer as outer helices (blue in Fig. 1). To add structural rigidity, a parallel inner-helix ridge (magenta in Fig. 1) is mounted on every middle helix. The inner-helix layer consists of short miniscaf strands, rather than the long M13 scaffold strand, which hybridize to the staple strands to form the scaffold-parity side of the DNA duplex^26^ (Supplementary Note 1). A useful feature of a DNA-origami barrel is the presentation of a rhombic lattice of strand nick points that can serve as pixel sites, each spaced ~8 nm apart from six nearest neighbors and available for potential functionalization by guests, on both inside (concave) and outside (convex) surfaces. As a comparison, the typical Rothemund rectangle is designed with nick points, projecting from one face, arranged in a rhombic lattice with ~5-nm spacing. We designed barrel monomers with diameters from 30 to 90 nm, and heights ranging from 19 (4 outer helices, 5 inner helices) to 65 nm (14 outer helices, 15 inner helices).

DNA-origami barrels were folded using standard folding conditions with yields of 68–85% (Supplementary Tables 1–4 and Supplementary Figs. 12–32). In TEM, the monomer particles appeared quite uniform (Fig. 2 and Supplementary Figs. 33–60). For 60- and 90-nm barrels that landed on their sides, flattening could be seen (e.g., measured diameters ~50% larger than designed diameters; see Supplementary Table 5 for statistics), presumably due to drying artifacts commonly seen in negative stain. Some of the 90-nm barrels appear to land on one point on the side and with the rest standing upright, leading to a distortion of the barrel. This indicates the increased flexibility of the 90-nm barrels, which is consistent with its larger diameter combined with its shorter height. For the particles that land with their barrel axis upright, a generally round profile can be seen. We interpret these deviations from perfect roundness as consistent with the limited thickness of the walls of the barrels (~6 nm). This highlights the design tradeoff between rigidity and wall thickness; if an alternative was to be pursued with thicker walls and therefore more rigidity, but otherwise the same outer dimensions, then the cost of materials per particle would be increased.

**Fig. 2:**
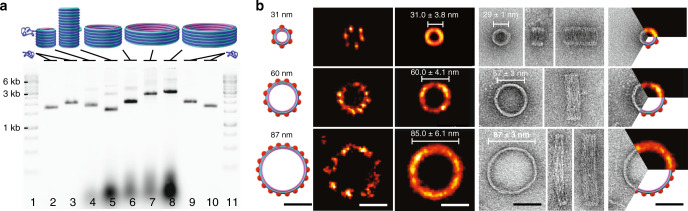
DNA-origami barrel monomers. **a** Agarose gel electrophoresis of five barrel designs. Lanes 4–8 are barrels with diameter–height in nm: 30–27, 30–65, 60–30, 90–19, 90–23. Controls are 1-kb ladder (250–10,000 bp, lanes 1, 11; reference bands marked for 1, 3, 6-kb dsDNA), and unfolded scaffold (7308-nt lanes 2, 10; 8634-nt lanes 3, 9). **b** Design and molecular microscopy of barrel monomers excised from agarose gels as in **a** and recovered by centrifugation. Imaging was performed via (middle) DNA-PAINT super-resolution fluorescence microscopy or (right) uranyl-formate negative-stain TEM. To prepare for DNA-PAINT imaging, monomers first were functionalized with 6, 12, 18 biotins on their bottom inner helices for upright attachment to streptavidin-coated glass surfaces, and 6, 12, 18 docking handles on one of the outer helices (for 30, 60, 90-nm diameter barrels, respectively; indicated by red dots in design schematic). DNA-PAINT images are given for a single structure (left) and as a summed image (right) of *N* = 726, *N* = 16, *N* = 27 particles for the 30, 60, 90-nm barrels, respectively. Design and measured diameters of barrels are indicated on panels for both imaging methods. Scale bars 50 nm.

The drying process inherent in negative-stain preparation is well known to create particle compression artifacts, especially pronounced for hollow objects such as our DNA-origami barrels, as we discussed above. In order to obtain more accurate measurements of the solution dimensions of barrels, we performed super-resolution imaging with DNA-PAINT^27–30^. This allowed the visualization of the DNA nanostructures in their native aqueous environment (Fig. 2b and Supplementary Figs. 61, 62). We functionalized the bottom ring of each barrel with 6, 12, 18 regularly spaced biotins on 30, 60, 90-nm diameter barrels, respectively, to facilitate attachment in an upright orientation on a streptavidin-coated glass slide. After super-resolution imaging, a set of *n* = 726, 16, 27 individual structures was selected for the 30, 60, 90-nm barrels respectively, and combined in a sum image by center-of-mass alignment. The monomer diameter in the sum image was determined with a histogram along the radial axes originating from the center of mass. Comparing designed (D) diameter versus measured DNA-PAINT (P) diameter in upright-oriented particles versus measured TEM (T) diameter in upright-oriented particles, we measured the following in nanometers: (D 31, P 31.0 ± 3.8, T 29 ± 1), (D 60, P 60.0 ± 4.1, T 57 ± 3), (D 87, P 85.0 ± 6.1, T 87 ± 3) (Supplementary Table 5).

### Coaxial assembly of DNA-origami barrel polymers

The interface between coaxially stacked barrels is formed by an additional outer helix (e.g., orange or yellow helix in Fig. 1 and Supplementary Figs. 1–11). Specific coaxial stacking is directed by staple-parity plugs that base pair to ssDNA sockets on the bottom of the cognate barrel (e.g., dark orange ssDNA outer miniscaf segments at the bottom of Fig. 1d). Both plugs and sockets are derived from short synthetic strands, therefore custom sequences can be designed to generate many orthogonal pairings without requiring a change to the core staple-strand sequences. With this design, pixel positions on the connector helix are in register with those present on the body of the barrel, and the rhombic-lattice pattern is continuously maintained (Supplementary Fig. 11).

**Fig. 3:**
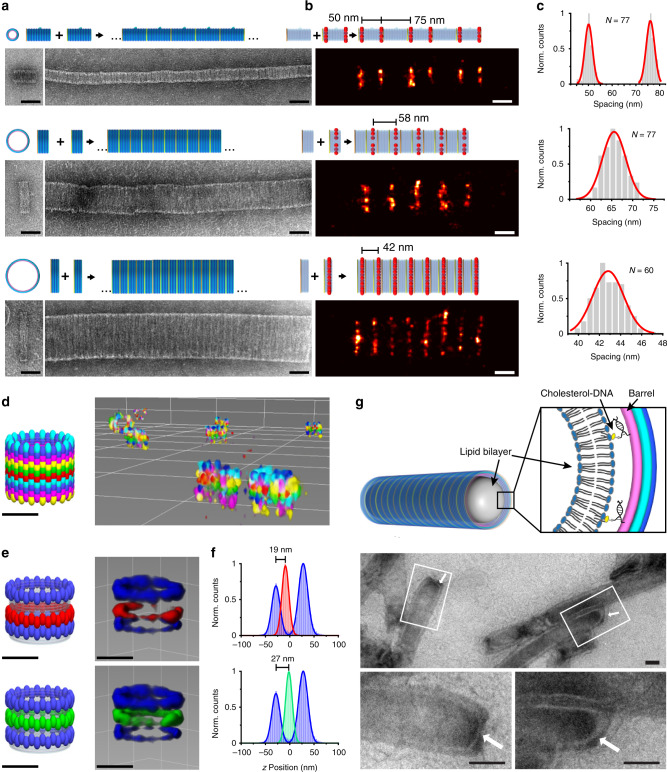
DNA-origami barrel polymers assembled from repeating *α* and *β* monomers. **a** Models of barrel polymers illustrating placement of DNA-PAINT docking handles. 30-nm barrel design: *α* monomer with two DNA-PAINT rings spaced 50 nm apart, and *αβ* repeating height of 125 nm; 60-nm barrel design: *α* monomer with one DNA-PAINT ring and *αβ* repeating height of 58 nm; 90-nm barrel design: *α* monomer with one DNA-PAINT ring and *αβ* repeating height of 42 nm. **b** DNA-PAINT images of representative polymers with corresponding models listing designed spacings above. (Scale bars in **a** and **b** are 50 nm). **c** Histograms of measured spacings of DNA-PAINT rings. **d** Model of 90-nm barrel *αβα* trimer with four DNA-PAINT rings for each monomer, showing DNA-PAINT images of individual particles (colors for *α*: cyan, blue, magenta, yellow, colors for *β*: yellow, green, red, cyan). **e** Composite sum image (outer ring docking sites: X4, X5, X6, X1, X2, X3, X4, X5, X6, Supplementary Table 9). 3D model (left) shows designed ring spacing of 17 and 26 nm. **f** Histogram of distances between DNA-PAINT rings, measured ring spacing of 19 ± 10 and 27 ± 10 m. **g** Negative-stain TEM images of lipid nanotubes reconstituted within the interior of 90-nm barrel polymers. Scale bars are 50 nm.

We used DNA-PAINT to measure the periodicity in height of the barrel monomers within the polymers in a 2D projection and compare to TEM (Fig. 3a–c and Supplementary Figs. 63–69). First we functionalized the side walls of the DNA barrels with biotins (two or four per monomer) to facilitate lateral attachment to the streptavidin-coated surface. Using cross-sectional histogram measurements of *n* = 77, 77, 60 individual polymers for the 30, 60, 90-nm barrels respectively, the monomer barrel heights were determined (Supplementary Fig. 73 and Supplementary Table 5). Subsequent frequency count analysis of the measured distances in Fig. 3 yielded DNA-barrel heights of 63, 33, 22 nm for 30, 60, 90-nm barrels, respectively. Comparing designed (D) height versus DNA-PAINT (P) height in sideways-oriented particles versus TEM (T) height in sideways-oriented particles, we measured the following in nanometers: 30-nm barrel (D 62, P 63 ± 1.8, T 59 ± 1), 60-nm barrel (D 29, P 33 ± 2.8, T 26 ± 2), 90-nm barrel (D 21, P 22 ± 1.5, T 20 ± 1).

### Discrete DNA-origami barrel trimer and decamer

Next we constructed trimers of the 90-nm barrel functionalized with 18 biotins on the bottom ring for attachment in an upright orientation to enable independent measurement of height using 3D DNA-PAINT imaging (Fig. 3d–f and Supplementary Fig. 70)^29^. For the trimer, we encoded distinct pseudocolors as orthogonal docking-site sequences. These could be imaged in separate rounds with Exchange-PAINT, where each round addresses one pseudocolor through flow introduction of the corresponding fluorescent imager sequence. Simultaneous imaging of included tetrahedron DNA origami^30^ as registration markers enabled alignment of the individual pseudocolor images into a composite. Individual colors were aligned to each other in a two-step process (Supplementary Fig. 74). First, coarse alignment in *xy* direction between each round was performed using cross-correlation. Next the registration markers were selected, and with center-of-mass calculations, target-independent fine alignment was employed in *xyz* direction. Cross-sectional histogram analysis along the *z* axis yields a monomer height of 28 $\pm$ 7 m (cf. designed height of 25 nm). In one imaging round, we achieved FWHM spatial resolution in *z* of 17 nm, which easily allows us to resolve rings spaced by 52 nm within the same channel (pairs of blue rings in Fig. 3e, f and Supplementary Fig. 70).

Functionalizing many pixels on the outer staple strands with ssDNA handles, and the combination of both many plugs and many handles, contributes to poor monomer yields, due to aggregation during folding (Supplementary Figs. 15 and 16). The concern over unwanted multimerization at interfaces is consistent with a larger theme in structural DNA nanotechnology, where large arrays of ssDNA segments presented on one side of a DNA shape (e.g., edge of a DNA origami) constitute a sticky interface prone to unwanted interactions through cooperative linkage of weakly complementary sequences. Weak complementarity can be attenuated by use of three-letter codes, especially ones that are composed of adenosines, cytosines, and thymidines to avoid guanosine, which is the most promiscuous base. We discuss additional strategies to mitigate unwanted multimerization in Supplementary Note 2.

In order to assemble a discrete stack of ten monomers (i.e., ~90-nm wide, ~250-nm high), we designed nine orthogonal interfaces and distributed the subcomponents to ten different monomers each folded in a separate tube (Supplementary Figs. 19 and 20). Monomers were purified using gradient centrifugation (Supplementary Fig. 30), either separately or else first combined and then purified as a pool (this pooling strategy is much less laborious). Purified monomers then were combined together in the presence of masking strands (to reduce unwanted aggregation, Supplementary Fig. 29) and outer miniscaf strands (to mediate coaxial stacking). As with the uncontrolled length polymers, increased occupancy of pixel positions with handles led to lower yields of decamer assembly due to unwanted multimerization. Decamers could be enriched by another round of glycerol gradient purification (Supplementary Fig. 31). The highest yield was achieved for the bare decamer, at ~5% relative to amount of input scaffold initially added during monomer folding. The lowest yield was observed for the decamer with all 2124-pixel sites occupied, at ~0.7% (Supplementary Fig. 26 and Supplementary Table 4). Purified decamers were observed to be stable when stored either at 4 °C or −20 °C (Supplementary Fig. 32) over a period of at least 3 months.

### Addressability of DNA-origami barrel assemblies

**Fig. 4:**
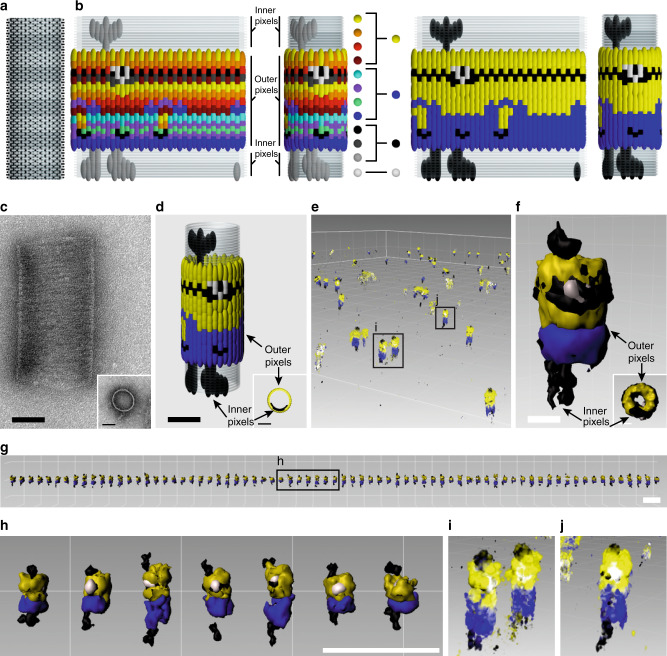
Molecular microscopy of DNA-origami barrel decamers patterned with complex features (Supplementary Movies 1–6). **a** Model of decamer, showing all possible pixel locations for DNA-PAINT docking handles. **b** Model of designed pattern of docking handles, for flattened and side-view (orthographic) of decamer. The design includes both outer and inner pixels. Pixels are colored by the 12 rounds of Exchange-PAINT (left) or grouped as four pseudocolors for ease of visualization (right). Model pixel size represents DNA-PAINT FWHM-resolution. **c** TEM of sideways decamer. Inset, TEM of upright monomer. **d** Model of designed placement of DNA-PAINT docking handles by pseudocolour, perspective view. **e** Exchange-PAINT image of a field of upright patterned particles alongside a few registration particles, with volume rendering. **f** Composite sum image with surface rendering. Inset, view from above. **g** Computationally arranged panel of selected particles with surface rendering. **h** Zoomed-in view seven particles boxed in **g** with surface rendering. **i**, **j**, Zoomed-in volume rendered particles boxed in **e**. Scale bars are 50 nm for **c–f** and are 500 nm for **g**, **h**.

To demonstrate the exquisite addressability of the decamer DNA origami, we performed a 12-plex 3D-Exchange-PAINT experiment with these nanostructures decorated with docking sites in a complex pattern, shown in Fig. 4b, c and Supplementary Figs. 75, 76. This pattern includes features on both the outer and inner surfaces of the decamer. After reconstruction of every round and alignment using registration markers (same procedure as described above), *n* = 72 structures were picked to create a sum image using center-of-mass alignment and 3D cross-correlation (Fig. 4c). Individual rounds with the same visualization color were grouped together and rendered separately (Supplementary Fig. 77). Nearest neighbor analysis in *xy* direction yields localization precisions between 3.6 and 6.3 nm, translating to FWHM-resolution ~ 8.5 and ~14.8 nm (see Supplementary Table 8), using the NeNA metric of precision accepted in super-resolution microscopy^31^. We observed unexpected variations in the *z*-height of the particles (Fig. 4e, f and Supplementary Fig. 78). Further analysis indicated that the apparent *z*-height was correlated with the radial position of the particle in the field of view (Supplementary Fig. 79).

The efficiency of pegboard labeling can be estimated from the DNA-PAINT data to give an indication of staple incorporation. Generally for DNA origami, an average of ~80% strand availability for imaging and modification is observed using super-resolution imaging^32^. We measured an average of 71% (*N* = 741) staple availability for outer staples of the 30-nm barrel (Supplementary Fig. 62).

To demonstrate the templating potential of DNA-barrel polymers, we reconstituted lipid nanotubes within their interior cavities (Fig. 3g and Supplementary Note 3)^33^. Oligonucleotide-conjugated lipid molecules are site-specifically placed onto the interior surface of the 90 nm DNA-origami nanotube, which nucleate the formation of an enclosed lipid nanotube (Fig. 3g and Supplementary Figs. 80–82). This demonstrates that barrels can be used to scaffold large lipid nanotubes within their interior cavity. In contrast, unwanted aggregation was the result when it was attempted to scaffold the lipid nanotube formation on the outside of the barrels.

## Discussion

Here we have described design and folding optimizations for DNA-origami barrels that can function as versatile three-dimensional pegboards. Although a large number of applications may be well served by two-dimensional pegboards (i.e., Rothemund rectangle), nevertheless many other important applications can be better served with three-dimensional analogs. In particular, some applications demand separation of an inside compartment from an outside compartment, for example to insulate against interactions between those two environments. Example applications for compartments include drug-delivery vehicles, nanoreactors such as assembly chaperones or degradation factories, depots for triggered release. An important class of applications is as molds for assembly of other material. Molds can circumscribe the maximum dimensions of polymer assembly on the inside or outside^34^ (e.g., nanowires). Conversely, molds can block unwanted interactions between independent assemblies. For example, here we show that lipid nanotubes can be templated inside but not outside the barrels, and we recently demonstrated scaffolding of large lipid nanodiscs inside of DNA-origami barrels^35^. This strongly suggests that the DNA barrel can act as a bumper to prevent unwanted aggregation of molecular assemblies hosted on the inside. We also observed some general limitations of 3D DNA-origami assembly, including the incomplete labeling efficiency of functionalized staples^32^, and the relatively low assembly yields of large multimers with complex patterning^3^. Recently, we have also shown stabilization of barrels for use in physiological conditions^36,37^.

The modularity of DNA-barrel structures serves to promote rapid uptake of this technology in diverse fields beyond specialist groups working in DNA nanotechnology. The barrels are a set of precise, robust, and optimized templates for the 3D arrangement of matter on the nanoscale. Our DNA-barrels incorporate key features for usability and accessibility to nonspecialists, such as regular arrangement of surface pixels for functionalization with guest molecules, and are thoroughly experimentally characterized. These structures will facilitate diverse applications by allowing for rapid prototyping of a range of functional nanodevices, incorporating guest molecules from metallic nanoparticles and polymers for nano-electronics and plasmonics, to proteins, lipids and therapeutic molecules for single-molecule biology and targeted drug delivery.

## Methods

### Assembly of DNA-barrel monomers

DNA barrels were designed using software caDNAno^24^. Detailed description of the design is given in Supplementary Note 1, design schematics are shown in Supplementary Figs. 1–11, and DNA sequences in Supplementary Data 1–8, cadnano files are included as Supplementary Software. Design-specific staple strands were purchased from IDT Technologies, the scaffold strand (p7308, p8064, p8634) was produced from M13 phage replication in Escherichia coli. DNA barrels were folded by mixing scaffold (p8634 for 90–23, p8064 for 60–32, p7308 all other designs) with tenfold excess of staples and miniscaf short scaffold-parity strands in folding buffer containing 5-mM Tris, 1-mM EDTA, pH, and with MgCl_2_ from 6 to 20 mM. Samples were thermally annealed in a PCR machine (Tetrad 2 Peltier thermal cycler, Bio-Rad). Annealing ramps include: (1) 65–25 °C for 18, 66, or 72 h, (2) 65 °C for 15 min and 50–40 °C for 18, 66, or 72 h, and (3) 65 °C for 15 min and 47 °C for 18, 66, or 72 h. Optimal folding conditions varied with the design. Optimal folding of 30–27 barrel is at 8-mM MgCl_2_ 47 °C for 18 h. For all other structures optimal folding is at 10-mM MgCl_2_ with a ramp of 65 °C for 15 min and decreasing linearly from 50 to 40 °C for either 66 or 72 h.

### Hierarchical assembly of polymers and multimers

Monomers were folded with 10× excess of staple-parity plugs to form the coaxial connection interface. Each monomer with a unique interface sequence set was folded in a separate pool. For each design, monomers were folded either with or without the outer miniscaf connector strands, based on which condition gave the highest monomer yield by gel analysis. Monomer designs folded with 10× excess connector strands are: 30–27, 30–65, 60–27, and 90–19. Designs folded without connector strands are: 60–32 and 90–23. For monomers folded with connector strands, unique monomers were purified separately and then combined in a second step (i.e., hierarchical assembly). Designs that are folded with connector strands assemble into multimers on mixing. Designs that are folded without connector strands (60–32 and 90–23) have no active interface when folded as monomers (only plugs, no sockets), and assemble into multimers only on addition of connector strands to step 2. For these designs, monomers were combined first and then purified together as a single pool, followed by addition of connector strands.

Additional yield improvements during hierarchical assembly were achieved by including a thermal ramp from 40 to 20 °C (12 h), as compared to fixed incubation at 25 °C, and increasing the magnesium concentration to 20 mM as compared to 10 mM during this incubation period. For designs with complex outer pixel decorations, addition of masking strands in 1.5× excess to total docking-site concentration was found to reduce aggregation and increase yield of correctly formed multimers.

### Decamer assembly workflow

The overall process for assembling the 90–23-nm decamer (Fig. 4) involves the following steps.

1. Fold ten monomers in separate pools.
2. Run analytical gel to quantify the relative yield of the monomers.
3. Pool monomers with volumes weighted by relative yield estimated by gel.
4. PEG precipitate combined monomer pool.
5. Purify monomer pool by glycerol gradient purification and then immediately add mask strands for outer docking sequences present in the design, at 1.5× concentration of each docking sequence.
6. PEG precipitate purified monomer pool.
7. Measure concentration of monomers by UV absorption at 260 nm (Nanodrop, Thermo Scientific), assuming that A260 = 1 for 50 μg ml^−1^ for double-stranded DNA.
8. Trigger second assembly step by adding:
  - connector strands for all nine interfaces, 10× excess to total monomer concentration
  - Mask strands, 1.5× excess to number of docking sites per sequence in design.
  - MgCl_2_ up to 20-mM final concentration.
9. Anneal in PCR machine from 40 to 20 °C for 12 h.
10. Purify decamers by glycerol gradient purification.
11. Add mask strands back into purified decamer fractions.
12. Dialysis into folding buffer to remove excess glycerol (slide-a-lyzer Mini, 10 kDa, Thermo Scientific).
13. For DNA-PAINT imaging, wash mask away after incubating samples with coverslip.

### Calculation of DNA-barrel decamer assembly yields

Monomer yields are calculated by UV absorption of the purified samples, either as a pool or as separate monomers, using Mw of 9.05 mDa. Decamer yields were calculated by UV absorption measurements of final samples after using calculated combined molecular weight of the 90–23-nm decamer of 90.5 mDa.

### Agarose gel electrophoresis

After folding, monomer samples were analyzed by agarose gel electrophoresis (2% agarose, 0.5× TBE 10-mM MgCl_2_, 2-μL ethidium bromide) using Thermo Scientific EasyCast Mini Gel System. Twenty microliter of each sample was added to 4 μl of 6× loading buffer (60% glycerol, 1× TE, 0.02% bromophenol blue) prior to loading. GeneRuler DNA Ladder 1 kb, 250–10,000 bp (ThermoFisher SM0311) was added as a control lane. The samples were run for 2–3 h at 65 V at room temperature with running buffer: 0.5× TBE, 11-mM MgCl_2_. For decamer samples, gels were run similarly, but with 1.5% agarose and with run times of 5–6 h. Gels were imaged with Typhoon FLA 9000 (GE Healthcare Life Sciences). To recover purified samples, bands were excised from the gel on a UV transilluminator, crushed, and extracted using a DNA spin column (Freeze and Squeeze, Bio-Rad) in a benchtop centrifuge at 13,000 *g* for 20 min at 4 °C. Band intensities were analyzed using Bio-Rad Imagelab software.

### Purification by gradient ultracentrifugation

DNA-barrel monomers were purified by a rate-zonal centrifugation procedure using a 15–45% (v/v) glycerol gradient^38^. The 45 and 15% glycerol solutions were made in 1× TE buffer with 10-mM MgCl_2_. For all size barrel monomers, samples were spun in Beckman SW55-Ti rotor at 36,6942 *g* (55,000 rpm) at 4 °C for 1 h. For 90–23-nm decamers, samples were spun in Beckman SW41-Ti rotor at 287,730 *g* (41,000 rpm) at 4 °C for 25 min. Following ultracentrifugation, gradients were fractionated into 200-µL fractions by an automated system (Gradient Station, BioComp) with UV detection at 260 nm.

### PEG precipitation

Purified barrel monomer and decamer samples were concentrated by PEG precipitation^39^. Samples were combined 1:1 by volume with 2 × PEG stock (20% PEG 20k, 2-M NaCl, 20-mM MgCl_2_), incubated at 4 °C for 30 min, then precipitated by spinning in a cooled benchtop centrifuge (Eppendorf) 15,000 *g*, 30 min, 4 °C. Supernatant was discarded, and samples then spun again at 15,000 *g*, 5 min, 4 °C, followed by removal of the last of the supernatant with small volume (10 μL) micropipette. Precipitated samples were resuspended in 25 μL of 1× folding buffer (1× TE, 10-mM MgCl_2_), and quantified by UV absorption.

### Transmission electron microscopy

Three microliter of DNA-barrel samples were pipetted onto a plasma-treated carbon Formvar grid (Electron Microscopy Sciences) and incubated for 1 min. The solution was wicked away onto filter paper, and 7 μl of freshly prepared 2% uranyl formate (in H2O, w/v) was immediately added. This was incubated for 20 s and then wicked away by filter paper. Imaging was carried out on a JEOL 1400 TEM at 80 kV in bright-field mode.

### TEM image processing

To estimate DNA-barrel diameters from TEM data, we utilized built-in phase-coding circular hough transform-based algorithm in MATLAB 2018a (Supplementary Fig. 72). For height estimation, DNA-barrel objects were extracted morphologically from thresholded images. Barrel objects, isolated from these images, were subsequently reoriented to a vertical layout. A 2D cross-correlation was computed between the individual barrel images and a library of binary images of rectangles that were pre-generated with varying heights and widths (Supplementary Fig. 71).

### DNA-PAINT sample preparation

Streptavidin was ordered from Invitrogen (cat: S-888). Tris 1 M pH 8.0 (cat: AM9856), EDTA 0.5 M pH 8.0 (cat: AM9261), magnesium 1 M (cat: AM9530G), and sodium chloride 5 M (cat: AM9759) were ordered from Ambion. Ultrapure water (cat: 10977-035) was purchased from Gibco. For Exchange-PAINT experiments ibidi sticky-Slides VI 0.4 (cat: 80608) were purchased from ibidi. Coverslips were purchased from Marienfeld (cat: 0107042). PCD (cat: P8279-25UN), PCA (cat: 37580-25G-F), and Trolox (cat: 238813-1G) were ordered from Sigma-Aldrich.

Three buffers were used for sample preparation and super-resolution imaging: buffer A (10-mM Tris-HCl pH 7.5, 100-mM NaCl, 0.05% Tween 20, pH 7.5); buffer B (5-mM Tris-HCl pH 8, 10-mM MgCl_2_, 1-mM EDTA, 0.05% Tween 20, pH 8); buffer C (1× PBS pH 7.2, 500-mM NaCl). 100× Trolox: 100-mg Trolox, 430-μl 100% methanol, 345-μl 1-M NaOH in 3.2-ml H_2_O. 40× PCA: 154-mg PCA, 10-ml water and NaOH were mixed and adjusted to pH 9.0. 100×PCD: 9.3-mg PCD, 13.3 ml of buffer (100-mM Tris-HCl pH 8, 50-mM KCl, 1-mM EDTA, 50% glycerol).

Samples were prepared for Exchange-PAINT in ibidi sticky-Slide^29^. For Exchange-PAINT chamber preparation, a piece of coverslip (no. 1.5, 24 × 60 mm^2^, ~0.17-mm thick) was placed at the adhesive of an ibidi sticky-Slide and pressured with a pipette tip. First, 40 μl of biotin-labeled bovine albumin (1 mg/ml, dissolved in buffer A) was flown into the chamber and incubated for 5 min. Then the chamber was washed using180 μl of buffer A. Second, 40 μl of streptavidin (0.5 mg/ml, dissolved in buffer A) was then flown through the chamber and incubated for 5 min. Next, the chamber was washed with 180 μl of buffer A and subsequently with 180 μl of buffer B. Then ~500 pM of the (premixed) DNA-barrel structures were flown into the chamber and allowed to attach to the surface for 8 min. Finally, the imaging buffer with buffer B with dye-labeled imager strands was flowed into the chamber. Imager concentrations of the experiments can be found in Supplementary Tables 6–8. When multiple imager sequences were used simultaneously, the final concentration of each imager sequence was inversely weighted with the number of docking sites on the DNA-barrel structure. Previous imagers from an Exchange-PAINT round were removed with 4 × 180-μl washing with buffer B. The liquid exchange was performed using an electric syringe pump with tubing fixed to one of the ibidi sticky-Slide chamber outlets. To minimize shift of the sample chamber when new buffers were introduced into the chamber, the buffers were dripped contactless into the second outlet using a pipette.

### DNA-PAINT super-resolution microscopy

Super-resolution imaging for Fig. 3 was carried out on an inverted Nikon Ti-Eclipse microscope (Nikon Instruments) with the Perfect Focus System. For the experiment, an oil-immersion objective (Apo TIRF 100′, NA 1.49, Oil, Nikon Instruments) was used. As excitation laser, a 561 nm (300-mW nominal, Coherent Sapphire) was used. Excitation light was filtered with a laser cleanup filter (zet561/10x, Chroma Technology Corp). As dichroic, a laser dichroic mirror was used (zt561rdc-UF2, Chroma Technology Corp). Fluorescence light was spectrally filtered with an emission filter (et600/50 and et575lp, Chroma Technology Corp) and imaged on a sCMOS camera (Andor Zyla 4.2, Oxford Instruments).

Super-resolution imaging for Figs. 2 and 4 was carried out on an inverted Nikon Ti-Eclipse microscope (Nikon Instruments) with the Perfect Focus System. For the experiment, an oil-immersion objective (Apo SR TIRF 100′, NA 1.49, Oil, Nikon Instruments) was used. As excitation laser, a 561 nm (200-mW nominal, Coherent Sapphire) was used. Excitation light was filtered with a laser cleanup filter (zet561/10x, Chroma Technology Corp). As dichroic, a laser dichroic mirror was used (zt561rdc-UF2, Chroma Technology Corp). Fluorescence light was spectrally filtered with an emission filter (et600/50 and et575lp, Chroma Technology Corp) and imaged on a sCMOS camera (Andor Zyla 4.2, Oxford Instruments). Astigmatism for 3D imaging was introduced with the commercial N-STORM Adapter (Nikon Instruments).

### Super-resolution data processing

Super-resolution DNA-PAINT reconstruction, drift correction, alignment, and averaging was carried out using the software package Picasso^29^. The DNA-barrel data were first drift-corrected with redundant cross-correlation. Second, the DNA-barrel structures were picked using Picasso’s semiautomated particle picking tool. All picked structures were examined manually to only select drift nanostructures and exclude target structures. Residual drift was then corrected with the picked drift nanostructures as fiducial markers. The same selection of structures was then used to align the different Exchange-PAINT rounds. Two-dimensional super-resolution data were rendered with Picasso render. The NeNA metric was used for a realistic estimate of resolution^31^.

### 3D particle data processing

3D particle sum images were created using the module Average3 from the software package Picasso. The source code is publicly available: https://github.com/jungmannlab/picasso/. 3D sum images were generated by projecting 3D localizations to 2D planes and summation in 2D. For this, a two-dimensional cross-correlation approach was used^29^. Summing was performed interactively by performing alignment operations in individual 2D planes with increasing oversampling, e.g., first alignment in *xy* with oversampling 10, then *xz* with oversampling 10, then again *xy* with oversampling 20. Careful manual inspection the sum image results and adjustment of parameters are required to avoid optimizing to local minima.

For the sum image of multiple Exchange-PAINT rounds, all datasets were loaded simultaneously. To find an optimal rotational angle, the normalized cross-correlation value for each round and angle was stored in a matrix. The optimal angle was chosen to be the angle where the sum of all cross-correlation values is maximized. Translation and rotation were applied to all rounds so that the relative alignment between rounds is preserved. When obvious artefacts could be observed (e.g., when locking-in on an Exchange-round with few localizations) individual rounds were selected and deselected during interactive averaging steps.

The resulting 3D super-resolution sum images were imported as voxels (voxel size was chosen to match the *x*–*y*–*z* spatial resolution) to Bitplane Imaris 9 (Oxford Instruments) and rendered with the particular pseudocolor.

### DNA-barrel lipid nanotube/liposome reconstitution

DNA barrels were encapsulated by annealing lipid-oligonucleotides to the nanostructure in a surfactant buffer^33^. The annealed product was purified by glycerol gradient, then mixed with lipid and surfactant. This was then dialyzed to remove surfactant. In a typical experiment, a 200-μL solution was prepared, containing 5-nM DNA barrel mixed with a 10 molar excess (relative to handle number) of lipid-oligonucleotide conjugates in buffer (containing 5-mM Tris, 1-mM EDTA, 12-mM MgCl_2_, pH 8, 2% octyl glycoside (OG)). The solution was incubated for 18 h at 30 °C on a Tetrad 2 Peltier Cycler (Bio-Rad). The annealed product was purified from excess lipid-DNA conjugates via glycerol gradients containing 2% OG surfactant. Glycerol gradients were prepared using solutions of 15% glycerol with 2% OG and 45% glycerol with 2% OG in buffer. The annealed product was layered on top of the gradients and centrifuged in Beckman SW55-Ti rotor for 1 h at 55,000 rpm (36,6942 *g*) (for SW-55Ti rotor tubes). The gradients were then fractionated, and appropriate fractions were combined and concentrated back to the starting volume (i.e., 400 μL) using an Amicon 30K device. The concentration of the product was determined by UV absorbance at 260 nm on Nanodrop. Liposomes were added by transferring 0.5 vol of prepared liposomes into the solution. This was mixed on a Thermomixer at 300 rpm at room temperature for 1 h. A volume of reconstitution buffer (containing 5-mM Tris, 1-mM EDTA, 12-mM MgCl_2_, pH 8) equivalent to the current total volume was added and mixed gently. The entire solution was then transferred into an appropriately sized 7K MWCO Slide-a-Lyzer dialysis cassette (Thermo Scientific). The cassette was floated in 2 L of reconstitution buffer for 48 h. After dialysis, the sample was recovered from the dialysis cassette and concentrated using an Amicon 30K column. Reconstituted nanostructures were separated from excess lipids by equilibrium centrifugation using iodixanol (OptiPrep reagent, Sigma-Aldrich) gradient (35, 28, 18, and 8% from bottom to top). The gradient solutions were layered into ultracentrifuge tubes and centrifuged in Beckman SW41-Ti rotor at 287,730 *g* (41,000 rpm) for 5 h at 4 °C. The gradient was fractionated, and 50 μL of each fraction was transferred into a 96-well fluorescence plate (BD Bioscience).

### Reporting summary

Further information on research design is available in the Nature Research Reporting Summary linked to this article.

## Supplementary information

Supplementary Software

Supplementary Data 1

Supplementary Data 2

Supplementary Data 3

Supplementary Data 4

Supplementary Data 5

Supplementary Data 6

Supplementary Data 7

Supplementary Data 8

Supplementary Movie 1

Supplementary Movie 2

Supplementary Movie 3

Supplementary Movie 4

Supplementary Movie 5

Supplementary Movie 6

Supplementary Information

Reporting Summary

Description of Additional Supplementary Files

## Data Availability

All data are available in the manuscript or the supplementary materials. Any additional relevant data are available from the authors upon reasonable request.

## Code availability

Free software to view Cadnano design files for all DNA-origami barrel designs can be downloaded from https://cadnano.org/. We recommend use of cadnano v0.2.3 (honeycomb lattice) to view these files (https://cadnano.org/legacy.html).
