# Supplementary material for: Complex multicomponent patterns rendered on a 3D DNA-barrel pegboard
Source: Nat Commun. 2020 Nov 13;11:5768. doi: 10.1038/s41467-020-18910-x (PMC7666213; doi:10.1038/s41467-020-18910-x)
Supplement: Supplementary file 16 — Supplementary Information [file 41467_2020_18910_MOESM16_ESM.pdf]

Supplementary Information for

Complex multicomponent patterns rendered on a 3D DNA-barrel pegboard

Shelley F. J. Wickham[1,2,3,4,5,6] , Alexander Auer[7], Jianghong Min[1,2,3], Nandhini Ponnuswamy[1,2,3,4], Johannes B. Woehrstein[7], Florian Schueder[7], Maximilian T Strauss[7] , Jörg Schnitzbauer[7], Bhavik Nathwani[1,2,3], Zhao Zhao[1,2,3], Steven D. Perrault[1,2,3], Jaeseung Hahn[1,2,3], Seungwoo Lee[1,2,3], Maartje M. Bastings[1,2,3], Sarah W. Helmig[8], Anne Louise Kodal[8], Peng Yin[4,9], Ralf Jungmann[7], William M. Shih[1,2,3]

*To whom correspondence should be addressed. Email: William_Shih@dfci.harvard.edu

This PDF file includes:

Supplementary Notes 1-3

Supplementary Figures 1-82

Supplementary Tables 1-10

Supplementary data files include:

Supplementary Data 1-8 (DNA sequences)

Supplementary Data 9-23 (Cadnano design files)

Supplementary Movie files include:

Supplementary Movies 1-6

Table of Contents:

Supplementary Note 1: DNA Barrel Design **7**

Supplementary Note 2: Optimisation of coaxial interfaces to reduce aggregation. **10**

Supplementary Note 3: DNA barrel scaffolded lipid tubes and spheres **11**

Supplementary Note 4: Description of supplementary data and movie files **12**

Supplementary Figures **13**

Supplementary Tables **89**

Table of Supplementary Figures:

Supplementary Figure 1. Scaffold and staple paths, 30-27 nm barrel, core and coaxial 13
Supplementary Figure 2. Scaffold and staple paths, 30-65 nm barrel, core.. 14
Supplementary Figure 3. Scaffold and staple paths, 30-65 nm barrel, coaxial 15
Supplementary Figure 4. Scaffold and staple paths, 30-61 nm barrel, core.. 16
Supplementary Figure 5. Scaffold and staple paths, 60-27 nm barrel, core and coaxial 17
Supplementary Figure 6. Scaffold and staple paths, 60-32 nm barrel, core and coaxial 18
Supplementary Figure 7. Scaffold and staple paths, 90-19 nm barrel, core and coaxial 19
Supplementary Figure 8. Scaffold and staple paths, 90-23 nm barrel, core and coaxial 20
Supplementary Figure 9. Scaffold and staple paths, 120-15 nm barrel, core.. 21
Supplementary Figure 10. Pixel locations for representative barrel monomer: 90-23 nm monomer..... 22
Supplementary Figure 11. Pixel locations for coaxial barrel multimer: 90-23 nm dimer....................... 23
Supplementary Figure 12. Optimised folding yields of barrel monomers. 24
Supplementary Figure 13. 30-27 nm barrel folding optimisation. 25
Supplementary Figure 14. 30-65 nm barrel folding optimisation 26
Supplementary Figure 15. 60-27 nm barrel folding optimisation 27
Supplementary Figure 16. 90-19 nm barrel folding optimisation 28
Supplementary Figure 17. 90-23 nm barrel folding optimisation 29
Supplementary Figure 18. 90-23 nm barrel folding optimisation, coaxial 30
Supplementary Figure 19. 90-23 nm barrel folding optimisation, m0-m9 31
Supplementary Figure 20. 90-23 nm barrel folding optimisation, mask strands.................................... 32
Supplementary Figure 21. Quantification of monomer folding yields 33
Supplementary Figure 22. Schematic of coaxial interface variants 34
Supplementary Figure 23. 90-19 nm coaxial optimisation, sequence variants...................................... 35
Supplementary Figure 24. 90-19 nm coaxial optimisation, connection design variants 36
Supplementary Figure 25. 60-27 nm coaxial optimisation, connection and sequence variants 37
Supplementary Figure 26. 90-23 nm decamer gel yields for different pixel designs 38
Supplementary Figure 27. 90-23 nm barrel 3/5/10-mer assembly 39
Supplementary Figure 28. 90-23 nm decamer optimisation of assembly conditions............................. 40
Supplementary Figure 29. 90-23 nm decamer optimisation of pixel mask strands 41
Supplementary Figure 30. 90-23 nm monomer glycerol gradient purification 42
Supplementary Figure 31. 90-23 nm decamer glycerol gradient purification 43
Supplementary Figure 32. 90-23 nm decamer assembly process 44
Supplementary Figure 33. 3D models of DNA barrel monomers and controlled multimers 45
Supplementary Figure 34. Cropped TEMs, 30-27, 30-65, 30-131, and 30-61 nm. 46
Supplementary Figure 35. Cropped TEMs, 60-27, 60-32, and 60-98 nm.. 47
Supplementary Figure 36. Cropped TEMs, 90-19, 90-61 (3-mer), 90-23, 90-248 (10-mer), 120-15 nm48
Supplementary Figure 37. Field-of-view TEM 30-27 nm barrel 49
Supplementary Figure 38. Field-of-view TEM 30-65 nm barrel 49
Supplementary Figure 39. Field-of-view TEM 30-65 nm barrel dimer (height = 131 nm)...................... 50
Supplementary Figure 40. Field-of-view TEM 30-61 nm barrel 50
Supplementary Figure 41. Field-of-view TEM 60-27 nm barrel 51
Supplementary Figure 42. Field-of-view TEM 60-32 nm barrel 51
Supplementary Figure 43. Field-of-view TEM 60-27 nm barrel trimer (height = 98 nm)........................ 52
Supplementary Figure 44. Field-of-view TEM 90-19 nm barrel 52
Supplementary Figure 45. Field-of-view TEM 90-19 nm barrel trimer (height = 61 nm), unpurified...... 53
Supplementary Figure 46. Field-of-view TEM 90-23 nm barrel 53
Supplementary Figure 47. Field-of-view TEM 90-23 nm barrel decamer, purified 54
Supplementary Figure 48. Field-of-view TEM 90-23 nm barrel decamer, purified 54
Supplementary Figure 49. Field-of-view TEM 120-15 nm barrel 55
Supplementary Figure 50. 3D models of DNA barrel monomers and polymers.................................... 56
Supplementary Figure 51. Cropped TEMs, 30-27 nm polymer 57
Supplementary Figure 52. Cropped TEMs, 30-65 nm polymer 58
Supplementary Figure 53. Cropped TEMs, 60-27 nm polymer 59
Supplementary Figure 54. Cropped TEMs, 90-19 nm polymer 60
Supplementary Figure 55. Field-of-view TEM 30-27 nm barrel polymer 61

Supplementary Figure 56. Field-of-view TEM 30-65 nm barrel polymer **62**
Supplementary Figure 57. Field-of-view TEM 60-27 nm barrel polymer **63**
Supplementary Figure 58. Field-of-view TEM 90-19 nm barrel polymer **64**
Supplementary Figure 59. 30-65 nm barrel polymer, with and without coaxial brace strands **65**
Supplementary Figure 60. 90-19 nm barrel polymer, before and after interface design optimisation ... **66**
Supplementary Figure 61. 3D models of DNA-PAINT barrel monomers and polymers. **67**
Supplementary Figure 62 Cropped DNA-PAINT monomers **68**
Supplementary Figure 63 Cropped DNA-PAINT polymers, 30-65 nm, scale 100 nm **69**
Supplementary Figure 64 Cropped DNA-PAINT polymers, 60-27 nm, scale 100 nm **70**
Supplementary Figure 65 Cropped DNA-PAINT polymers, 90-19 nm, scale 100 nm **71**
Supplementary Figure 66. Field-of-view DNA-PAINT polymers, 90-19 nm, extended polymers **72**
Supplementary Figure 67. Field-of-view DNA-PAINT polymers, 30-65 nm, truncated.......................... **73**
Supplementary Figure 68. Field-of-view DNA-PAINT polymers, 60-27 nm, truncated.......................... **74**
Supplementary Figure 69. Field-of-view DNA-PAINT polymers, 90-19 nm, truncated.......................... **75**
Supplementary Figure 70. DNA-PAINT data for 90-23 nm trimer (Supplement to Figure 3)................. **76**
Supplementary Figure 71. Quantification of barrel height from TEM images **77**
Supplementary Figure 72. Quantification of barrel diameter from TEM images.................................... **78**
Supplementary Figure 73. Example quantification of barrel height from DNA-PAINT images **79**
Supplementary Figure 74. Particle alignment process for DNA-PAINT images **80**
Supplementary Figure 75. DNA-PAINT docking site pattern locations for Figure 4 .... **81**
Supplementary Figure 76. 3D models for DNA-PAINT docking site pseudocolor allocation................. **82**
Supplementary Figure 77. DNA-PAINT decamer pattern, rendered as separate pseudocolors **83**
Supplementary Figure 78. DNA-PAINT decamer pattern, volume and surface rendering **84**
Supplementary Figure 79. DNA-PAINT z-height variation over field-of-view **85**
Supplementary Figure 80. Lipid functionalization of DNA-barrel polymers (Supplement to Figure 3)... **86**
Supplementary Figure 81. DNA-barrel spherical-liposome reconstitution **87**
Supplementary Figure 82. DNA-barrel nanotube-liposome reconstitution **88**

Table of Supplementary Tables:

Supplementary Table 1. Monomer folding yields, quantified from agarose gel analysis 89
Supplementary Table 2. Purified 90-23 nm monomer yields, by monomer, by UV absorption.............. 90
Supplementary Table 3. Purified 90-23 nm monomer yields, averaged m0-m9, by UV absorption 90
Supplementary Table 4. Final purified 90-23 nm decamer yields, by UV absorption 91
Supplementary Table 5. Barrel dimensions from TEM and DNA-PAINT Quantification 92
Supplementary Table 6. Experimental conditions for super-resolution imaging in Figure 2 93
Supplementary Table 7. Experimental conditions for super-resolution imaging in Figure 3 94
Supplementary Table 8. Experimental conditions for super-resolution imaging in Figure 4 95
Supplementary Table 9. DNA-PAINT docking site sequences 96
Supplementary Table 10. DNA-PAINT imager sequences 97

Supplementary Data (in separate zip file):

Supplementary Data 1 - 30-27 nm barrel custom DNA sequences

Supplementary Data 2 - 30-65 nm barrel custom DNA sequences

Supplementary Data 3 - 30-61 nm barrel custom DNA sequences

Supplementary Data 4 - 60-30 nm barrel custom DNA sequences

Supplementary Data 5 - 60-32 nm barrel custom DNA sequences

Supplementary Data 6 - 90-19 nm barrel custom DNA sequences

Supplementary Data 7 - 90-23 nm barrel custom DNA sequences

Supplementary Data 8 - 120-15 nm barrel custom DNA sequences

Supplementary Data 9 - 30-27 nm barrel monomer Cadnano file

Supplementary Data 10 - 30-27 nm barrel coaxial interface Cadnano file

Supplementary Data 11 - 30-61 nm barrel monomer Cadnano file

Supplementary Data 12 - 30-65 nm barrel monomer Cadnano file

Supplementary Data 13 - 30-65 nm barrel coaxial interface Cadnano file

Supplementary Data 14 - 60-27 nm barrel monomer Cadnano file

Supplementary Data 15 - 60-27 nm barrel coaxial interface Cadnano file

Supplementary Data 16 - 60-32 nm barrel monomer Cadnano file

Supplementary Data 17 - 60-32 nm barrel coaxial interface Cadnano file

Supplementary Data 18 - 90-19 nm barrel monomer Cadnano file

Supplementary Data 19 - 90-19 nm barrel coaxial interface Cadnano file

Supplementary Data 20 - 90-23 nm barrel monomer Cadnano file

Supplementary Data 21 - 90-23 nm barrel coaxial interface Cadnano file

Supplementary Data 22 - 120-15 nm barrel monomer Cadnano file

Supplementary Data 23 - 90-23 nm barrel decamer Cadnano file

Supplementary Notes

Supplementary Note 1: DNA Barrel Design

Monomer Scaffold Architecture. Detailed scaffold and staple paths for all barrel monomers are shown in Supplementary Figures 1-11, and all follow the same general motif. The long scaffold strand forms the scaffold-parity regions of the outer and middle layers of the barrel (long black strand in Supplementary Figures 1-11). It starts at the top left, and traverses down through the helices via 4/3 turn intervals and antiparallel crossovers, then to the right and back up via 2/3 turn intervals and antiparallel crossovers. For the 30, 60, 90 and 120 nm diameter barrels, this pattern repeats 12, 24, 36 and 48 times across the sheet respectively. Each inner helix is formed from staple-strand extensions that emerge as antiparallel crossovers from its cognate middle helix at 21 bp intervals, that are base-paired to short scaffold-parity (miniscaf) strands (5). For our experimental implementation, we employ an m13-based sequence as the long scaffold strand, and custom-sequence oligonucleotides as miniscaf and staple strands.

Detailed scaffold and staple path schematics of all barrel designs are shown in Supplementary Figures 1-9. All show: 3D model (top-left), cross-section through barrel side-wall (top-middle) and zoom-in (top-right) of detailed paths for scaffold (black), outer staples (orange), inner-middle staples (green) and inner scaffold-parity staples (black). For coaxial connections, figures show 3D model and zoom-in the interface for monomer (middle) and polymer (bottom) states.

Monomer Outer staple strands. The outside (convex) nick points are implemented as the termini of outer-helix staple strands that are four turns long (i.e. ~15 nm) (orange staples in Supplementary Figures 1-11). These strands do not cross over to adjacent helices, (i.e. all crossovers between outer and middle helices are scaffold parity). Outer helices are 4.16 nm apart along the long axis of the barrel (Figure 3). Relative to adjacent outer helices, each outer nick point is staggered by two turns. Therefore, the two nearest-neighbor nick points along the barrel axis are two outer helices away, or ~8 nm apart. The four nearest-neighbor, off-axis nick points, which are on adjacent outer helices, are ~8 nm apart as well. In other words, the outer-helix nick points are arranged in a rhombic lattice with ~8 nm spacing (bright blue spheres in Figure 1a, Supplementary Figure 10).

Monomer Inner-middle staple strands. All crossovers between inner and middle helices are provided by inner-middle staple strands, at a density of one crossover per two turns (i.e. there are no scaffold-parity crossovers between these helices) (green staples in Supplementary Figures 1-11). Each inner-middle staple strand has a central domain within the inner helix and flanking domains within the middle helix. The inner-middle staple strands away from the top and bottom lips of the barrel are not used for functionalization and can be considered as invariant core elements.

Monomer Inner miniscaf strands. The inside (concave) nick points are implemented as the termini of inner-helix, short scaffold-parity (inner-miniscaf) strands that are four turns long (i.e. ~13 nm) (short black staples in Supplementary Figures 1-11). As with the outer staple strands, inner-miniscaf strands do not

cross over to adjacent helices, and inner helices are 4.16 nm apart along the long axis of the barrel. The inner-helix nick points (bright pink spheres in Figure 1b) are arranged in a rhombic lattice with ~8 nm spacing. The inner nick-point lattice does not overlay with the outer nick-point lattice. Instead, relative to the outer lattice, the inner lattice is shifted down by ~2.2 nm in the axial direction and rotated counterclockwise (looking from the outside perspective, as in Figure 1a left and Supplementary Figures 10,11) by 10 bp around the axis.

Monomer Barrel curvature. The basic pseudo-repeat of the 90 nm diameter barrel consists of an outer helix of 792 bp (72 turns, 11.00 bp/turn), a middle helix of 768 bp (72 turns, 10.67 bp/turn), and an inner helix of 720 bp (72 turns, 10.00 bp/turn) (Figure 1, Supplementary Figures 7,8). We also generated barrel versions with outer diameters of 60 nm (60 nm barrel, Supplementary Figures 5,6) and 31 nm (30 nm barrel, Supplementary Figures 1-4). For the 60 nm barrel, the basic pseudo-repeat consists of an outer helix of 540 bp (48 turns, 11.25 bp/turn), a middle helix of 516 bp (48 turns, 10.75 bp/turn), and an inner helix of 468 bp (48 turns, 9.75 bp/turn). For the 30 nm barrel, the basic pseudo-repeat consists of an outer helix of 270 bp (24 turns, 11.25 bp/turn), a middle helix of 240 bp (24 turns, 10.0 bp/turn), and an inner helix of 192 bp (18 turns, 10.67 bp/turn).

Monomer Barrel height variants. We designed different height (assuming 2.4 nm effective helix diameter) versions of each of these three diameter barrels: 90 nm wide, 23 nm tall (8568 nt of p8634 scaffold used) (Supplementary Figure 8) or 19 nm tall (7008 nt of scaffold p7308 used) (Supplementary Figure 7); 60 nm wide, 32 nm tall (7908 nt of p8064 used) (Supplementary Figure 6) or 27 nm tall (6852 nt of p7308 used) (Supplementary Figure 5); 30 nm wide, 65 nm tall (7110 nt of p7308 used; assembled using two-domain architecture) (Supplementary Figures 2,3) or 61 nm tall (6870 nt of p7308 used) (Supplementary Figure 4) or 27 nm tall (3300 nt of p7308 used) (Supplementary Figure 1).

Coaxial stacking interfaces. The interface between coaxially stacked barrels is formed by an additional outer helix (e.g. orange or yellow helix in Figure 1) whose scaffold-parity material is provided by outer miniscaf strands (i.e. not m13-based scaffold strand) that do not cross over. Specific coaxial stacking is directed by staple-parity plugs, 7 or 8 nt in length, that crossover from the top middle helix of one barrel (e.g. light yellow staple-strand extensions at the top of Figure 1, or shown in red in Supplementary Figures 1-11) that base pair to ssDNA sockets on the bottom of the cognate barrel (e.g. dark yellow ssDNA outer miniscaf segments at the bottom of Figure 1, or shown in black in Supplementary Figures 1-11). The outer miniscaf strands interact with twice the number of base pairs on one end (eg. bottom) of one barrel, as compared with the other end (eg. top) of the cognate barrel. In this design, crossovers occur every 7 or 8 basepairs. Therefore to avoid breaking staple-strand segments into shorter than 7 or 8 nt fragments, half crossovers were implemented (i.e. a nick is present on one strand at each crossover junction). Both plugs and sockets are derived from short synthetic strands, therefore custom sequences can be designed to generate many orthogonal pairings without requiring a change to the core staple-strand sequences. With this design, pixel positions on the connector helix are in register with those present on the body of the barrel, and the rhombic-lattice pattern is continuously maintained (Supplementary Figure 11).

Monomer pixel sites for DNA barrel functionalisation. Schematic (Supplementary Figure 10, top) shows 3' nicks on outer staples (blue) or inner scaffold-parity strands (magenta), which act as molecular pixels. Here they are depicted as functionalised with 10-nt DNA-PAINT docking sites. Inset (top): the DNA-PAINT imager strand transiently binds to the 10-nt docking sequence, which can be located either on outer (blue) or inner (magenta) surface of barrel.

Number of pixels. For 30, 60, 90, and 120 nm diameter barrel monomers, each outer and inner helix has 6, 12, 18, or 24 pixel sites respectively. Total number of pixel sites on barrel monomers are; 30-27: 78 px (36 outer, 42 inner), 30-65: 168 px (78 outer, 90 inner), 30-61: 162 px (78 outer, 84 inner), 60-30: 156 px (72 outer, 84 inner), 60-32: 180 px (84 outer, 96 inner), 90-19: 162 px (72 outer, 90 inner), 90-23 198 px (90 outer, 108 inner). 120-15: 168 total (72 outer, 96 inner). For coaxially stacked barrels, an additional helix of outer pixels is added at each connection interface, eg. 90-23 decamer has 10 monomers each with 198 pixels, and 9 interfaces with 18 pixels, giving 2142 pixels total (1062 outer, 1080 inner).

Pixel Masks. Functionalizing many pixels on the outer staple strands with ssDNA 'handles' also causes aggregation. The combination of both many plugs and many handles contributes to decreased monomer yields, due to aggregation during folding (Supplementary Figures 15,16,19**,** Supplementary Table 1). Coaxially stacked monomers tend to stick to each other laterally when many pixel sites are occupied (Supplementary Figure 66). Occupying these pixel handles with 10nt masking strands during hierarchical assembly helped to mitigate these unwanted interactions (Supplementary Figure 10), as observed by gel analysis of 90-23 nm decamer assembly (Supplementary Figure 29). To further limit this length-dependent effect, we included 5% capping monomers to reduce the average length of polymers for DNA-PAINT experiments (Figure 3, Supplementary Figures 67-69).

Coaxial barrel in multimer. Coaxial connector strands were modified to incorporate outer pixels as 3' extensions of staple 'plugs' on the top helix of the lower barrel of the interface (Supplementary Figure 11). For the 90-23 nm barrel there are 36 such 3' plug ends. An alternating design of 18 pixels is selected to achieve continuity of the hexagonal pixel array across the barrel interface. For coaxially stacked barrels, an additional helix of outer pixels is added at each connection interface, eg. 90-23 decamer has 10 monomers each with 198 pixels, and 9 interfaces with 18 pixels, giving 2142 pixels total (1062 outer, 1080 inner).

Capped polymers. Extended polymers labelled with DNA-PAINT docking sites were observed to aggregate into bundles (Supplementary Figure 66), as discussed above. Capping units, with either top or bottom helix lacking interface connectors, were added at 5% to total monomer concentration to assemble truncated polymers. These were used for DNA-PAINT imaging of barrel polymers in Supplementary Figures 67-69, and for quantification of barrel heights by DNA-PAINT.

Supplementary Note 2: Optimisation of coaxial interfaces to reduce aggregation.

Coaxial interface optimisation strategy. Generally, the concern over unwanted multimerization at interfaces is consistent with a larger theme in structural DNA nanotechnology, where large arrays of ssDNA segments presented on one side of a DNA shape (e.g. edge of a DNA origami) constitute a sticky interface prone to unwanted interactions through cooperative linkage of weakly complementary sequences.

Importantly, we found that design of longer plugs (e.g. 14 nt; 30-27, 30-65, 60-27, 90-19) (Supplementary Figures 1,2,5,7), as opposed to twice the number of plugs of half that length (60-32, 90-23) (Supplementary Figures 6,8), can contribute to unwanted aggregation during monomer folding (Supplementary Figures 15,16). This was especially the case for larger barrels that present a large number of these plugs (cf. 12 plugs for 30 nm barrels, 24 plugs for 60 nm barrels, 36 plugs for 90 nm barrels), with 90-19 nm barrel monomers being almost entirely aggregated (Supplementary Figure 16). Undesirable non-coaxial stacking during multimer assembly was observed by TEM (Supplementary Figure 60), and bundling of barrel polymers was observed in DNA-PAINT samples (Supplementary Figure 66)

The coaxial interface was optimised to reduce unwanted aggregation during monomer folding, as observed for initial designs of the 60-27 and 90-19nm barrels (Supplementary Figures 15,16), and non-coaxial stacking during multimer assembly, as observed by TEM (Supplementary Figure 60). It was found that this aggregation could be mitigated by design of hairpin protectors (Supplementary Figure 22), as seen in further gel analysis of monomer folding (Supplementary Figures 24,25). Weak complementarity can also be attenuated by use of three-letter codes, especially ones that are composed of adenosines, cytosines, and thymidines to avoid guanosine, which is the most promiscuous base. Our final staple strand designs used GC content ~50%, and G:C ratio ~1:4 (Supplementary Figure 23). For the 90-23 and 60-32 nm barrels, significant unwanted multimerization was observed when the outer miniscaf strands (i.e. that mediate coaxial stacking) were included during monomer folding (Supplementary Figure 23). To avoid this issue, outer miniscaf strands only were added after prior folding of monomer barrels and then mixing of cognate barrels.

Coaxial braces. Coaxial stacking was less robust for the 30 nm barrel, presumably due to the limited number of plug/sockets per interface. This resulted in obvious gaps between barrel monomers in polymers, as observed by TEM (Supplementary Figure 59). Stacking yield was improved by introduction of coaxial bracing on the inside of the barrel (Supplementary Figure 2).

Supplementary Note 3: DNA barrel scaffolded lipid tubes and spheres

DNA barrel scaffolded lipid tubes and spheres. Fabrication of lipid nanotubes with prescribed structure and composition parameters (i.e. diameter, lipid composition and protein stoichiometry) could provide a synthetic platform for in vitro study of complex biological phenomena involving lipid membranes. Previously we reported the use of DNA origami as templates to synthesize spherical liposomes with precisely controlled size[33] . Here we extended the method to non-spherical lipid structures through reconstitution of lipid nanotubes under mechanical confinement by our 90 nm diameter polymerized barrels (Figure 3g, Supplementary Figure 80-82).

Oligonucleotide-conjugated lipid molecules are site-specifically placed onto the interior surface of a DNA-barrel nanotube, which nucleate the formation of an enclosed lipid nanotube during a reconstitution procedure. The growth of the lipid nanotube is confined by the surrounding double-stranded DNA helices, so the final geometry of the lipid nanotube is complementary to hollow cavity within the DNA nanotube.

Supplementary Figure 80 illustrates the procedure to produce lipid nanotubes. We first assembled 90-23 nm barrels (75 nm inner diameter) bearing 54 ssDNA handles protruding towards the inside. The barrels were then polymerised. Lipid-DNA conjugates were added to hybridize with handles inside the DNA nanotubes, in the presence of detergent, followed by addition of lipid molecules. During removal of detergent by dialysis, homogenous lipid nanotubes were formed inside the DNA barrel polymers

As shown in Supplementary Figure 81, before lipid treatment, the tubular DNA-barrel structures were darkly stained throughout the interior. After lipid reconstitution, a very uniform and smoothly curved white layer was seen at the boundaries of the lumen of the DNA nanostructures (diameter: 74.4 ± 5.8 nm, N=45), especially on the two ends of the tube, suggesting that the inner space was lined with a lipid bilayer. Using gradient density ultracentrifugation, we purified the DNA-scaffolded tubular liposomes from excess lipid, TEM images indicate that more than 90% of the space inside DNA nanotubes were occupied by lipid nanotubes.

We also investigated decoration of the outside of a barrel polymer with lipid handles for templating lipid nanotube manufacture; previously we had success with this strategy for encapsulating 60-nm diameter DNA wireframe octahedra with high efficiency[33]. However, after decorating 30 nm-barrels with lipid-DNA conjugates, extensive aggregation of barrels was observed, even in detergent-containing buffer (4% OG). External decoration of DNA nanotubes with hydrophobic anchors lead to massive unwanted aggregation, while internal decoration of DNA nanotubes did not result in aggregation.

DNA barrel monomers were also used to capture liposomes of varying diameter. 90, 60 and 30 nm barrels with inner diameters of 75-nm, 48-nm, and 18-nm respectively, were assembled with 36, 24, and 12 lipid handles. After the lipid reconstitution procedure described above, TEM images (Supplementary Figure 81) showed that the 90, 60, and 30 nm barrels yielded liposomes with outer diameters of 69.1 ± 6.9 nm (N=52), 39.7 ± 3.8 nm (N=47) and 15.4 ± 2.1 nm (N=58), respectively.

Supplementary Note 4: Description of supplementary data and movie files

Supplementary Data Files 1-8. CSV files containing full DNA sequence databases for the staple and miniscaf strands for all barrel designs. DNA sequences are annotated as core strands or coaxial strands. Additional annotations give identity of strand as coloured in schematics and cadnano files: outer staples = light orange, inner-middle staples = light green, miniscaf (short scaf strand) = no colour, coaxial interface top = blue, coaxial interface bottom = red. Orthogonal coaxial connector sets are given as set 0, set 1, etc. Helix and base pair position numbers for start and end point of each strand are given, and correspond to position coordinates in cadnano files.

Supplementary Data Files 9-23. Cadnano design files for all DNA origami barrel designs. File names give barrel size and indicate monomer or coaxial version. Free software to view these files can be downloaded from https://cadnano.org/. We recommend use of cadnano v0.2.3 (honeycomb lattice) to view these files (https://cadnano.org/legacy.html).

Supplementary Movie 1. Movie illustrating the complex pattern rendered on the 90-23 nm decamer in Figure 4, comparing designed pattern and averaged data side-by-side, as in Figure 4d,f.

Supplementary Movie 2. Movie of averaged DNA-PAINT data for complex pattern rendered on the 90-23 nm decamer in Figure 4, rotating, as in Figure 4f.

Supplementary Movie 3. Movie of field-of-view DNA-PAINT data for complex pattern rendered on the 90-23 nm decamer in Figure 4, rotating and with zoom in to area highlighted in Figure 4i.

Supplementary Movie 4. Movie of individual particle DNA-PAINT data for complex pattern rendered on the 90-23 nm decamer in Figure 4, particles are selected and computationally aligned in a line to aid visual comparison as in Figure 4g,h.

Supplementary Movie 5. Movie of DNA-PAINT data for a single individual structure for complex pattern rendered on the 90-23 nm decamer in Figure 4, rotating, as highlighted in Figure 4j.

Supplementary Movie 6. Movie of DNA-PAINT data for two closely spaced individual structures for complex pattern rendered on the 90-23 nm decamer in Figure 4, rotating, as highlighted in Figure 4i.

Supplementary Figures

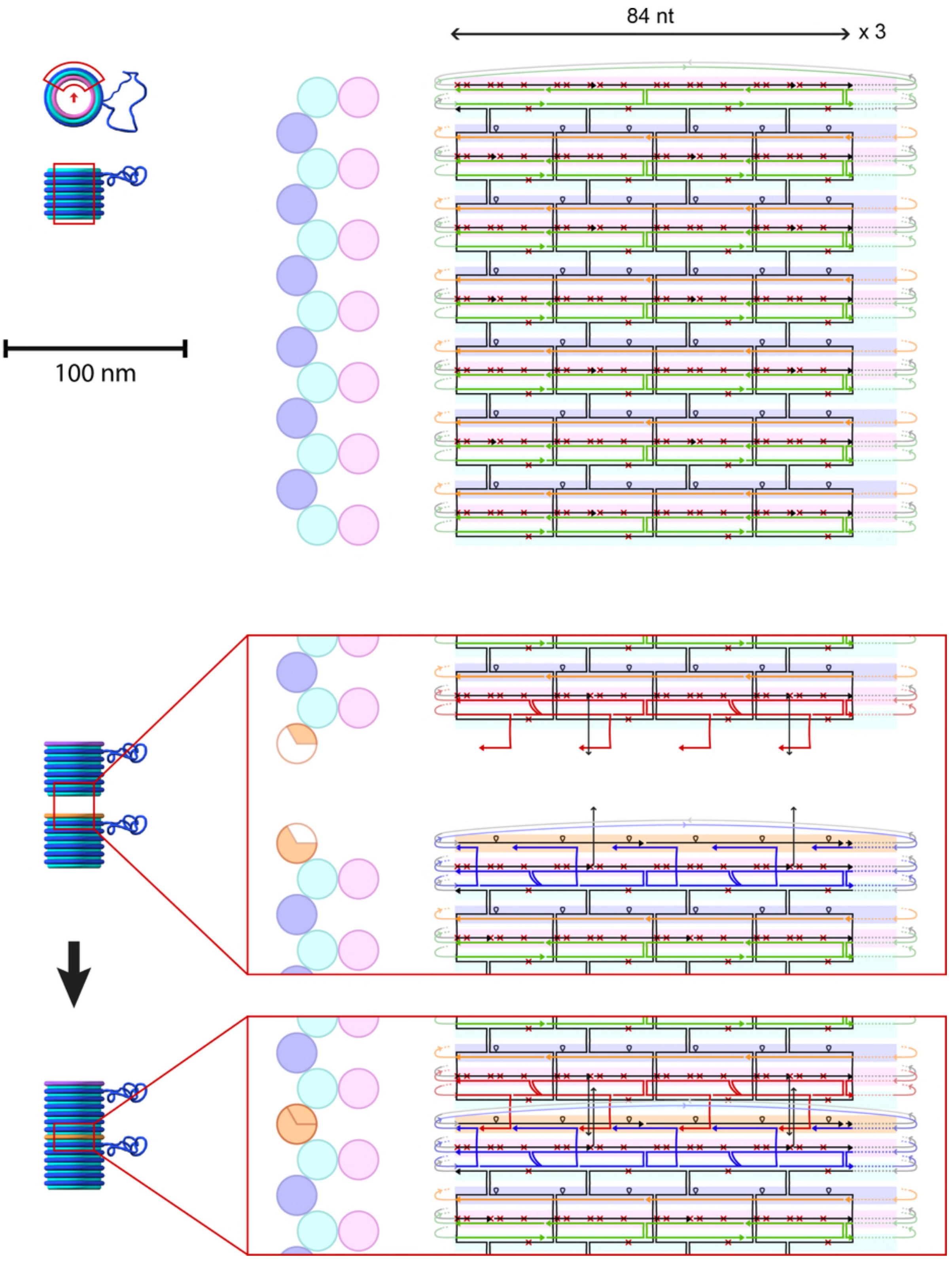

Supplementary Figure 1. Scaffold and staple paths, 30-27 nm barrel, core and coaxial

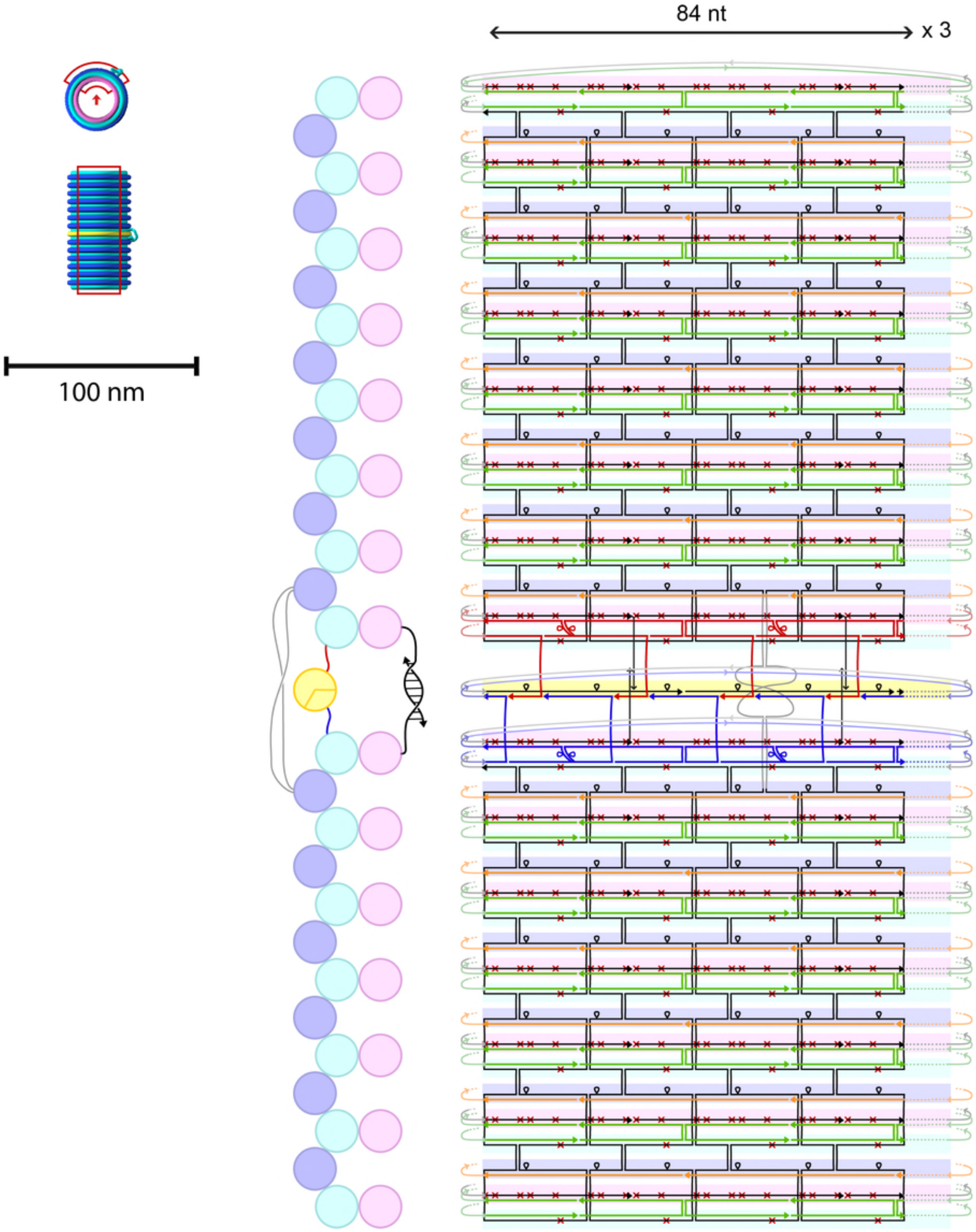

Supplementary Figure 2. Scaffold and staple paths, 30-65 nm barrel, core

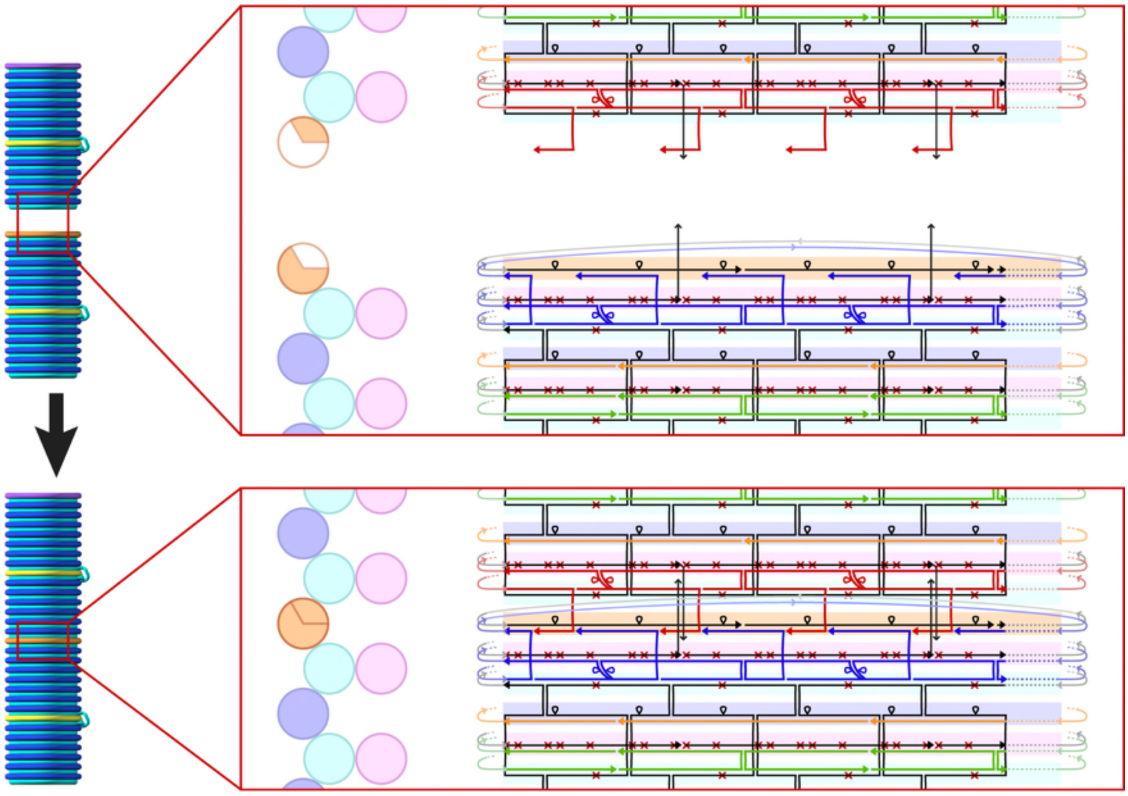

Supplementary Figure 3. Scaffold and staple paths, 30-65 nm barrel, coaxial

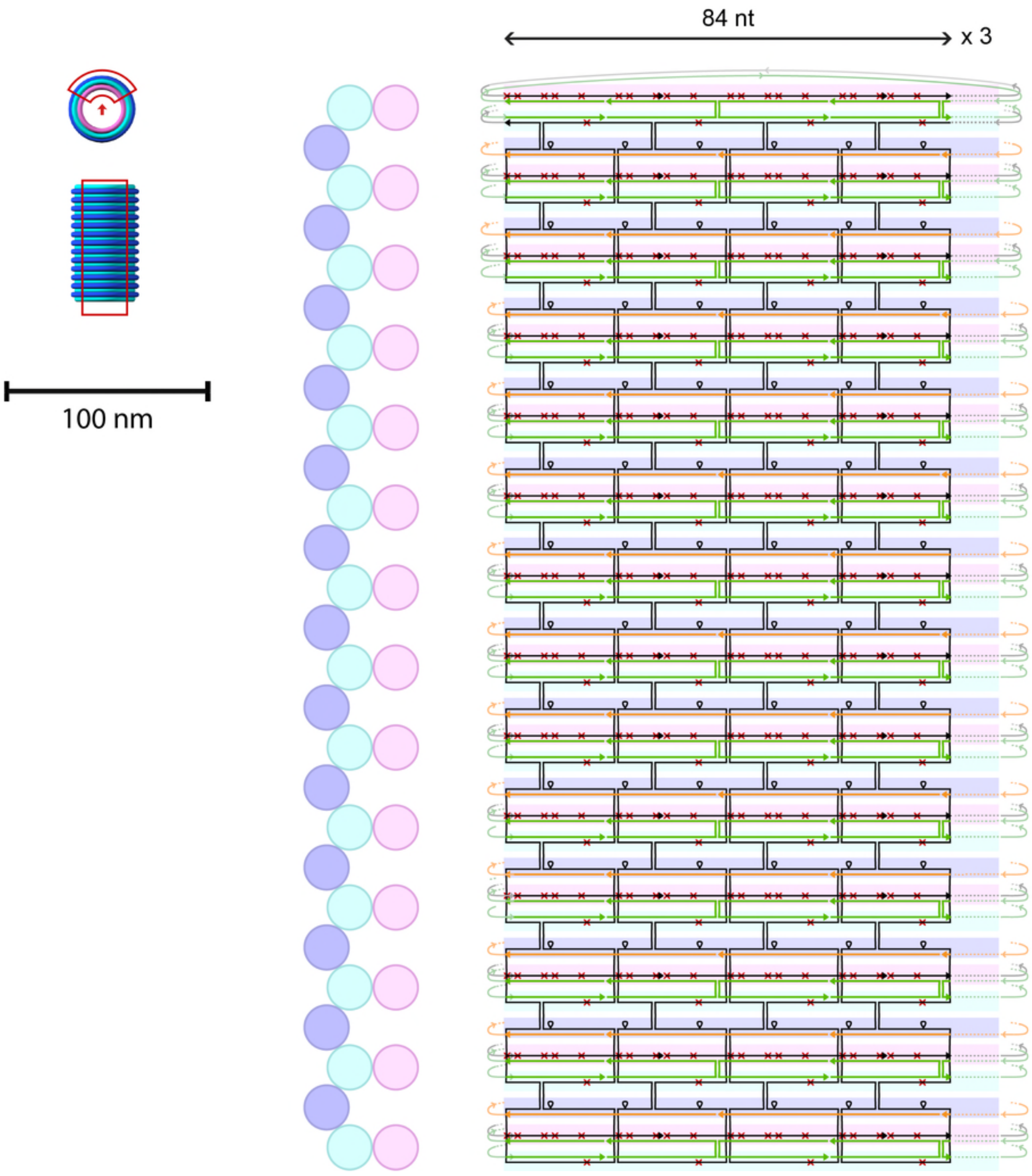

Supplementary Figure 4. Scaffold and staple paths, 30-61 nm barrel, core

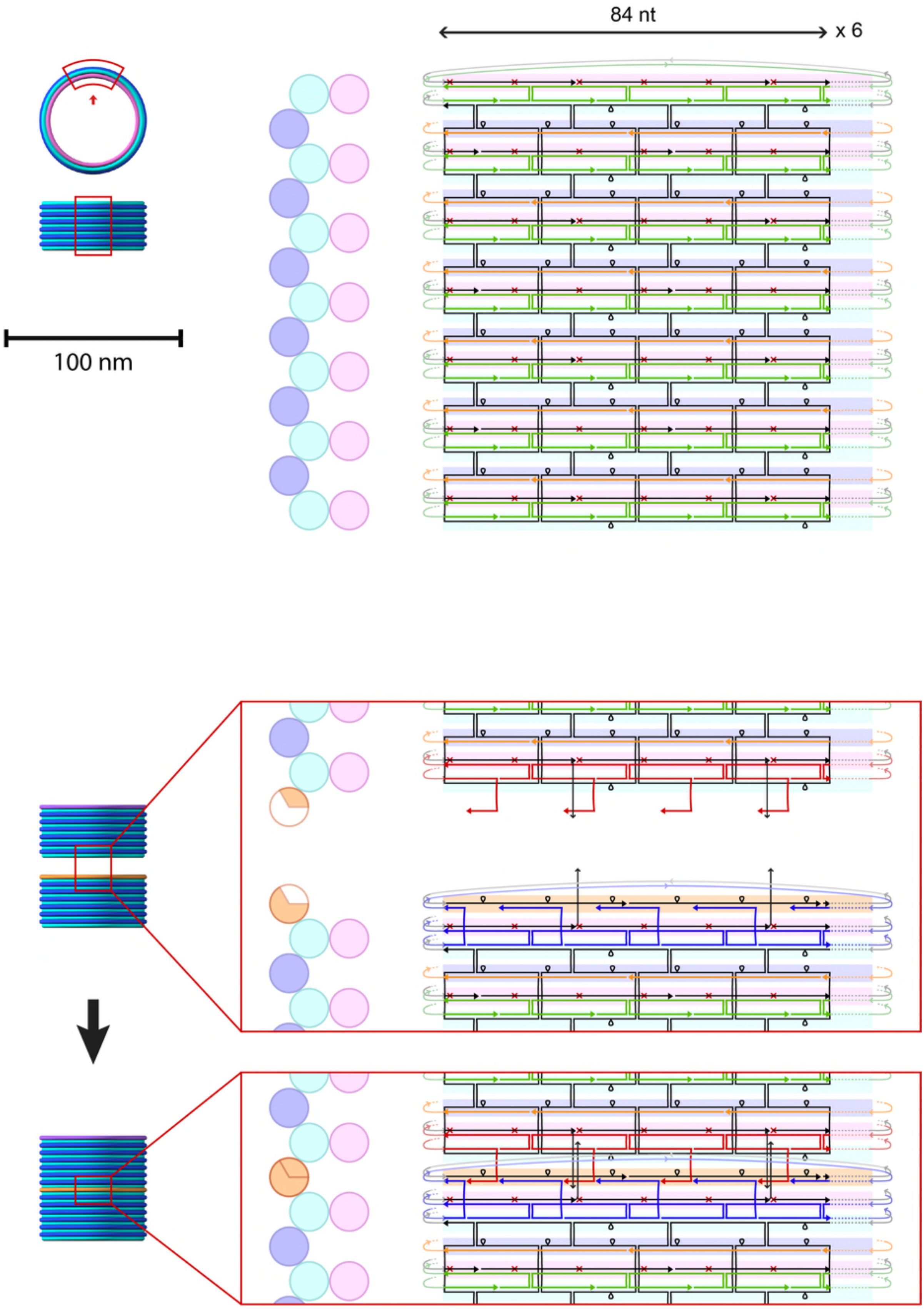

Supplementary Figure 5. Scaffold and staple paths, 60-27 nm barrel, core and coaxial

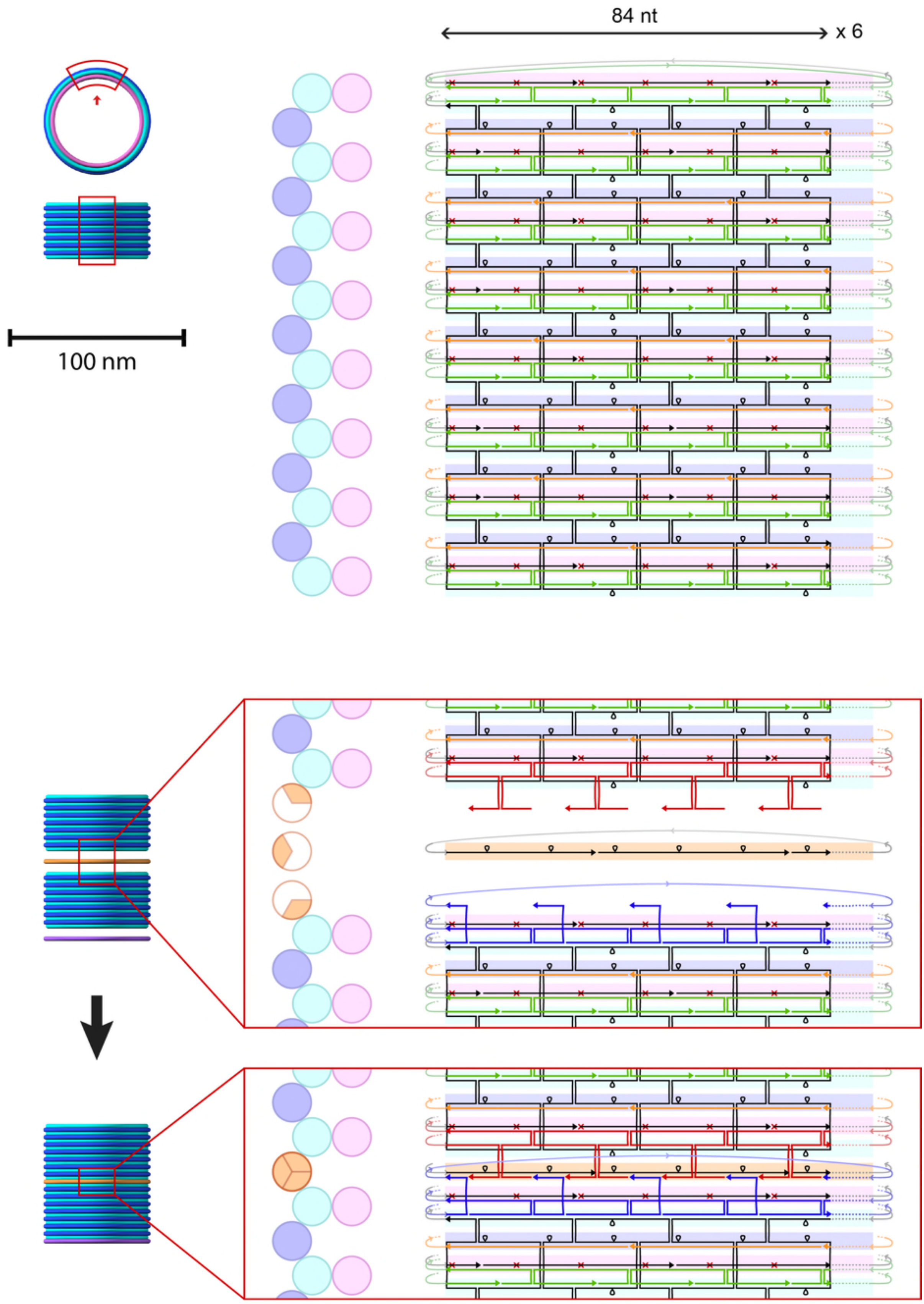

Supplementary Figure 6. Scaffold and staple paths, 60-32 nm barrel, core and coaxial

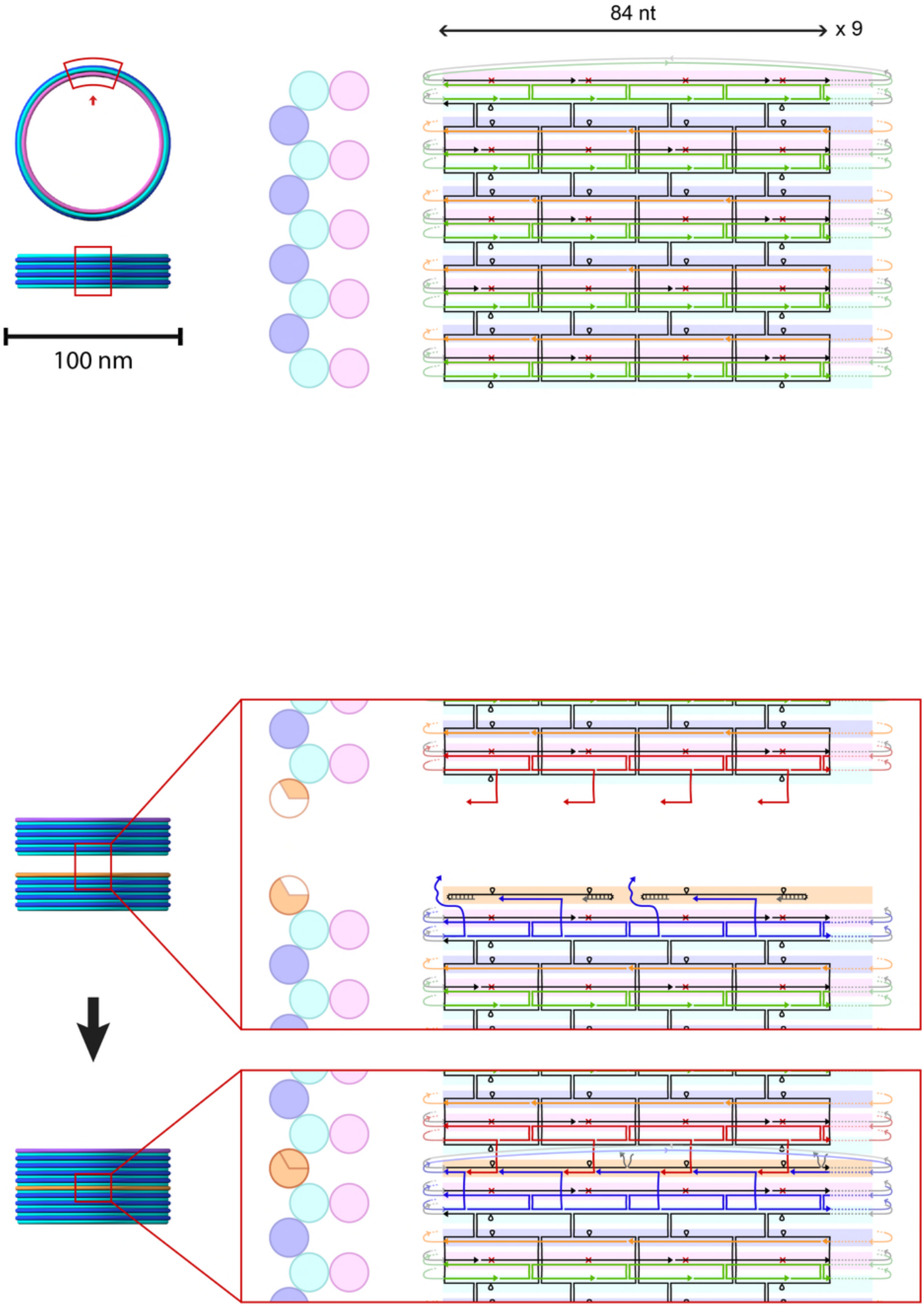

Supplementary Figure 7. Scaffold and staple paths, 90-19 nm barrel, core and coaxial

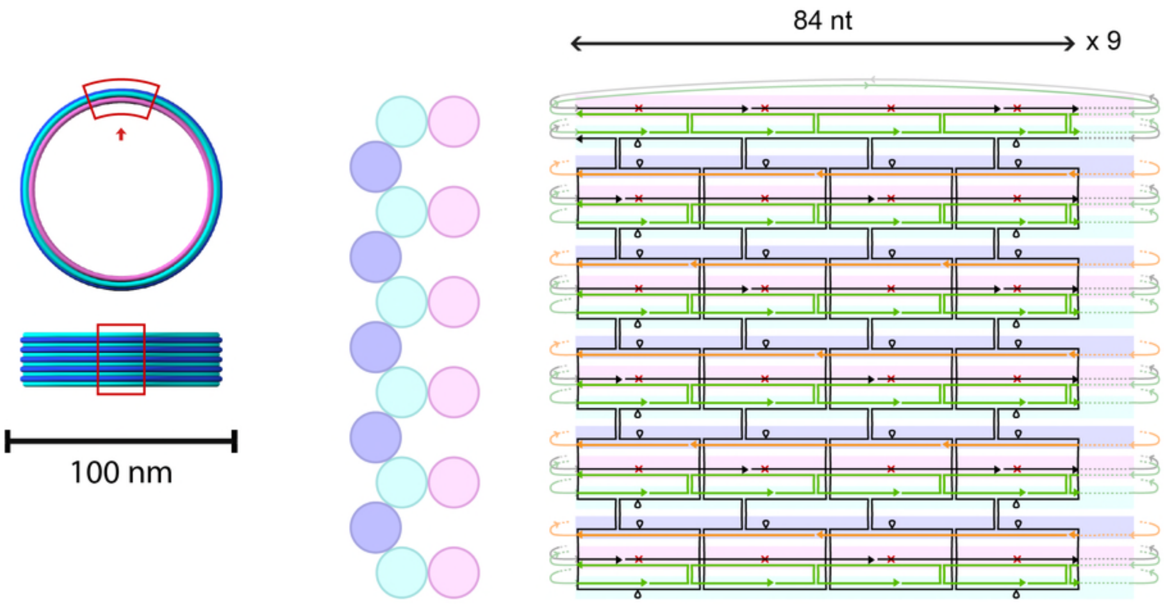

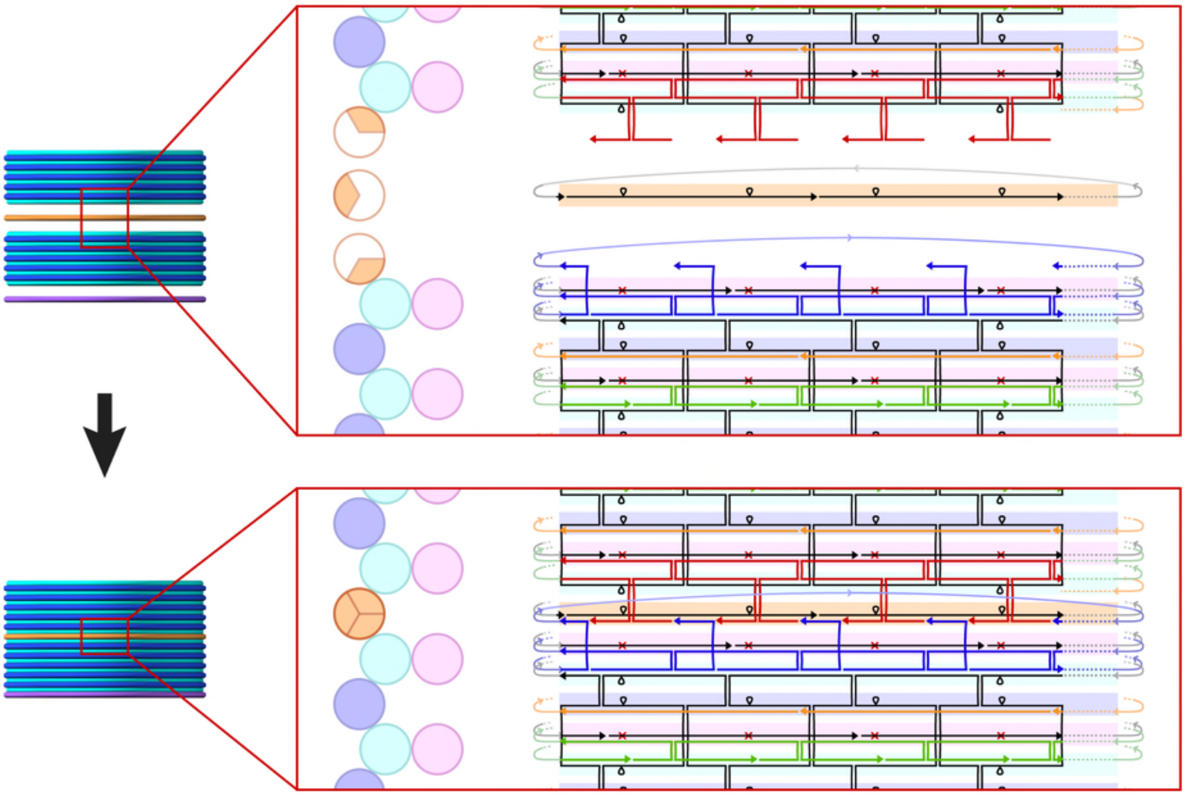

Supplementary Figure 8. Scaffold and staple paths, 90-23 nm barrel, core and coaxial

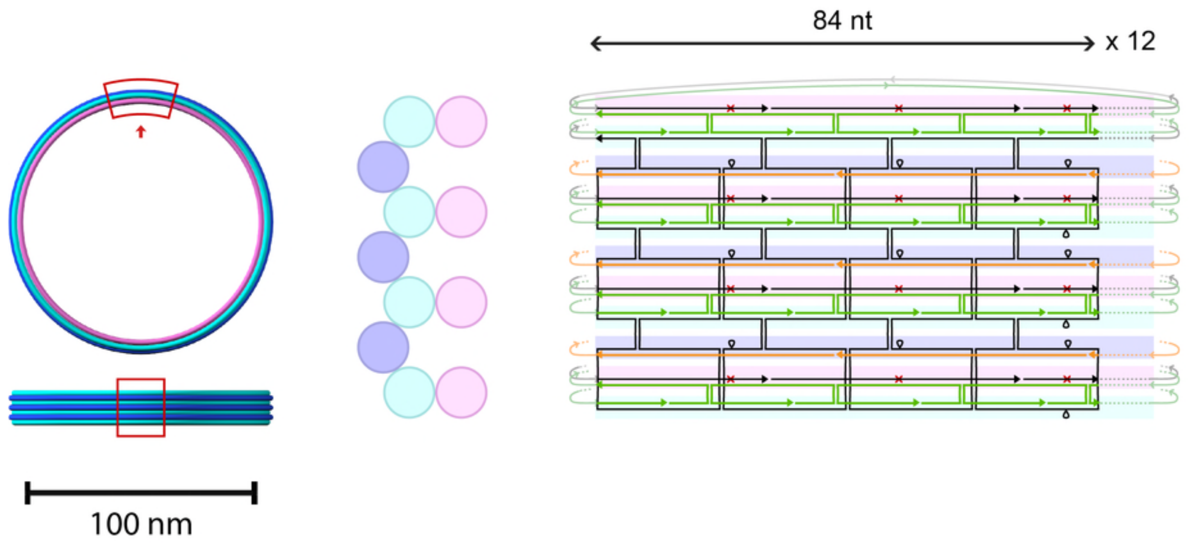

Supplementary Figure 9. Scaffold and staple paths, 120-15 nm barrel, core

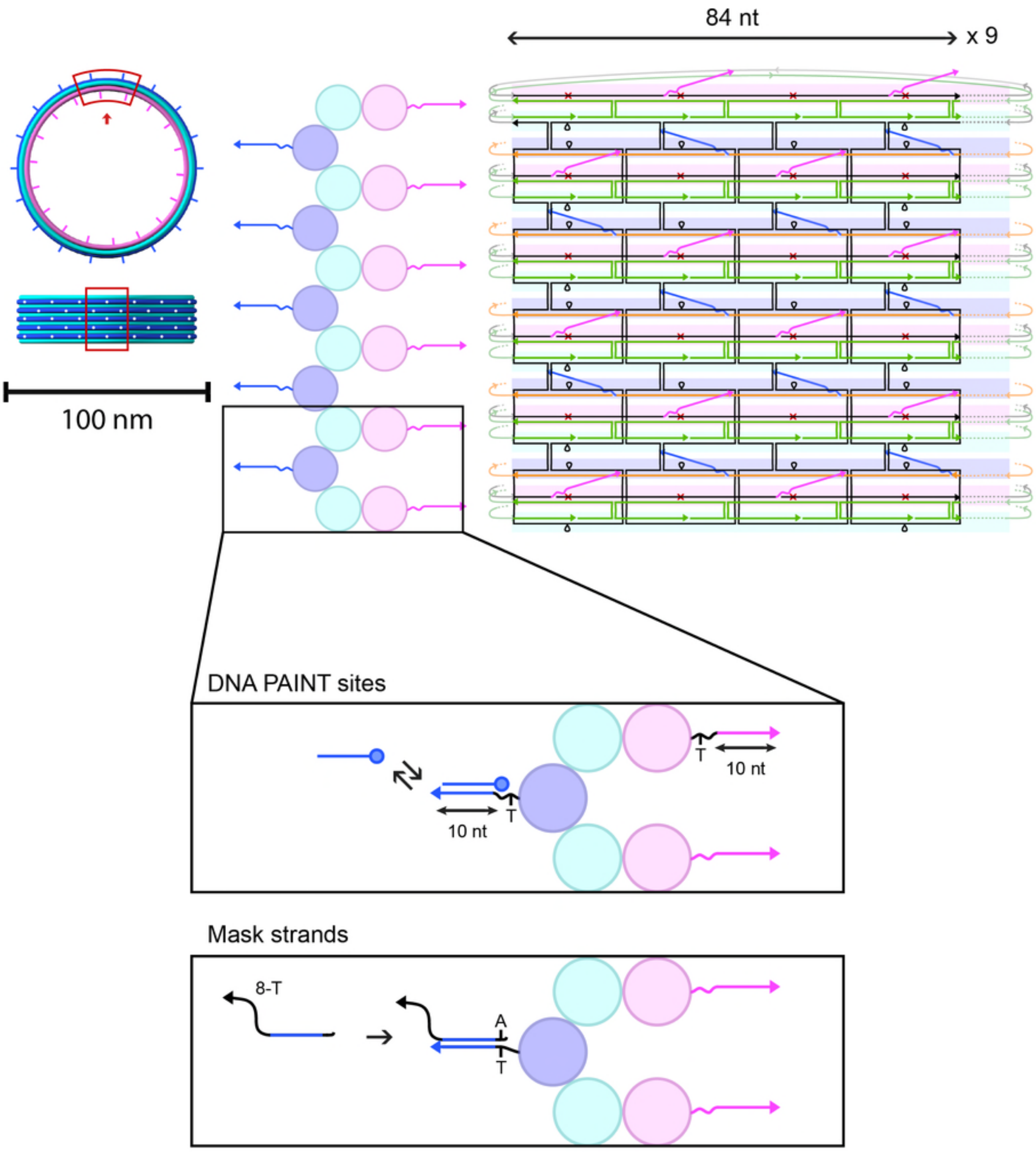

Supplementary Figure 10. Pixel locations for representative barrel monomer: 90-23 nm monomer. Inset (bottom): 19-nt 'Mask' strands are complimentary to the 10-nt PAINT docking site plus T-linker, and have a 5' 8-T overhang. Mask strands are added during decamer barrel assembly to reduce unwanted aggregation.

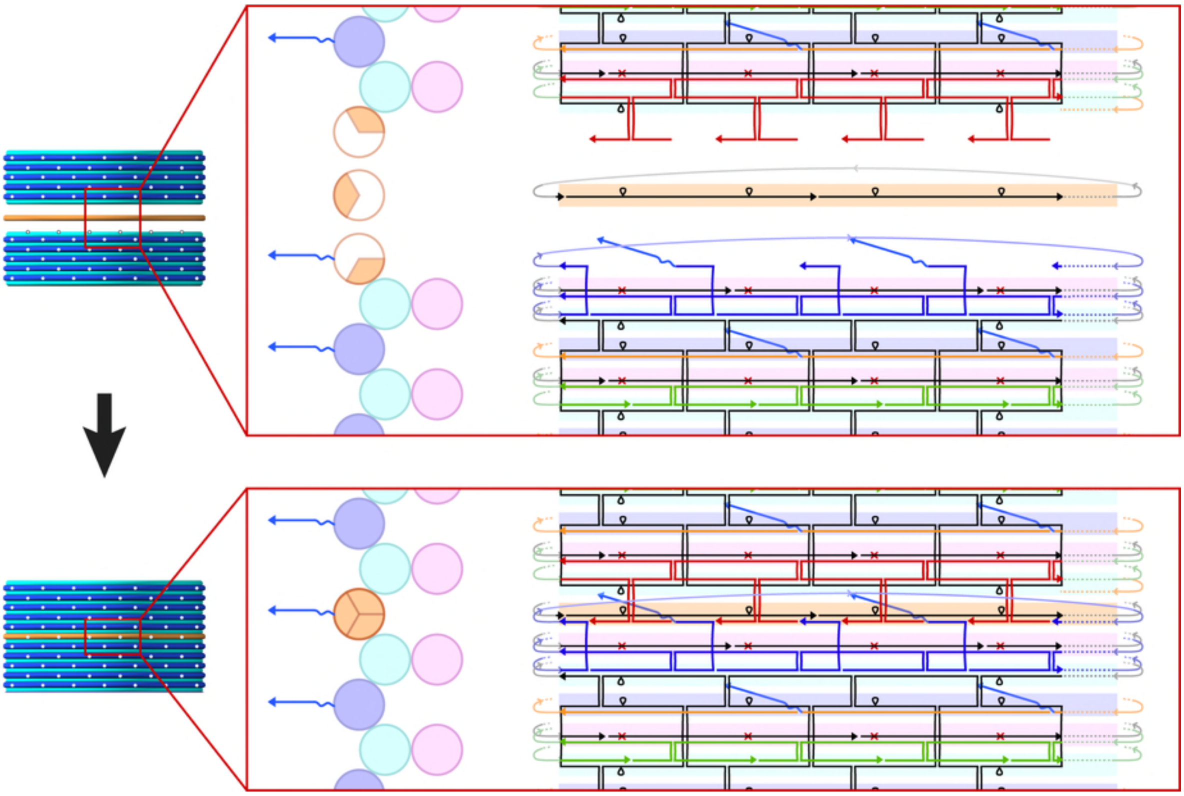

Supplementary Figure 11. Pixel locations for coaxial barrel multimer: 90-23 nm dimer.

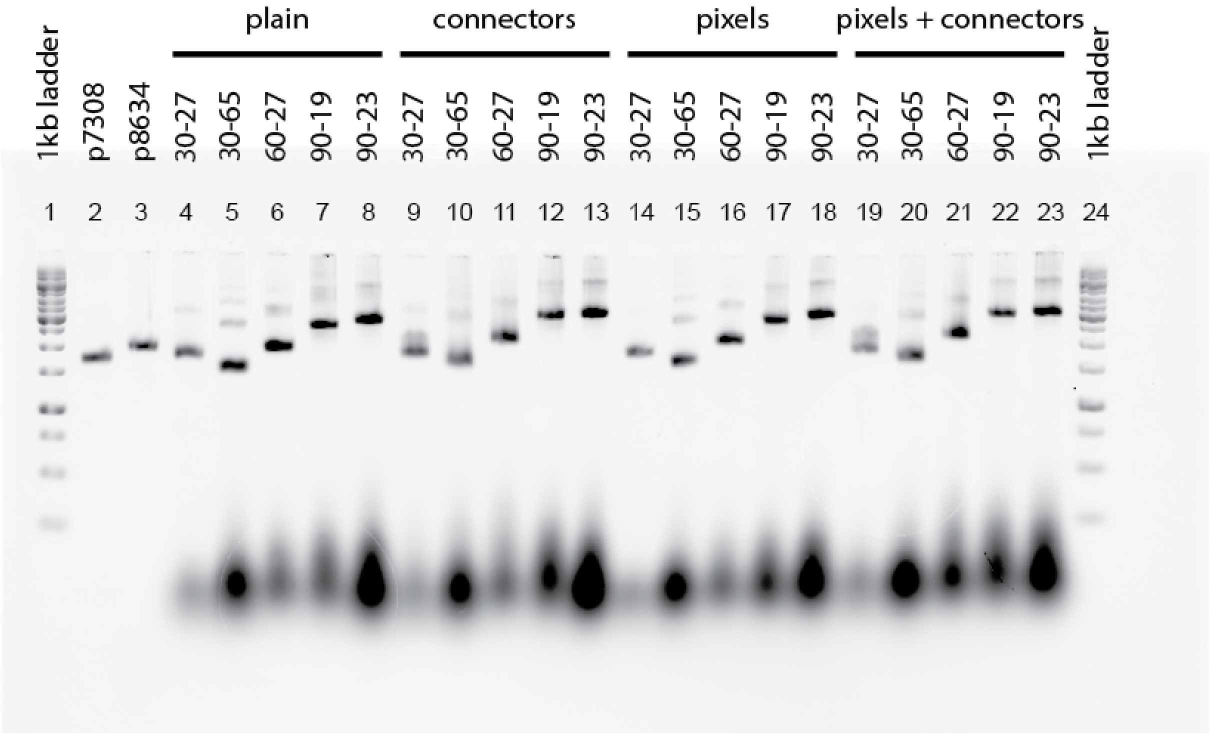

Supplementary Figure 12. Optimised folding yields of barrel monomers.

Agarose gel electrophoresis of five barrel designs (diameter-height in nm: 30-27, 30-65, 60-30, 90-19, 90-23). For each barrel design 4 conditions are shown: plain barrels (lanes 4-8), barrels with coaxial connector strands (lanes 9-13), barrels with outer DNA-PAINT pixel decorations (lanes 14-18), and barrels with both coaxial connectors and outer DNA-PAINT strands (lanes 19-23). Addition of pixel or connector strands to monomer folding reaction increases aggregation and decreases yield, adding both pixels and connectors decreases yield further. Folding conditions for 30-27 barrel were 8mM MgCl2, 65C for 15min, 47C for 18 hours. For all other samples folding conditions were 10mM MgCl2, 65C for 15min, 50-40C for 66 hours.

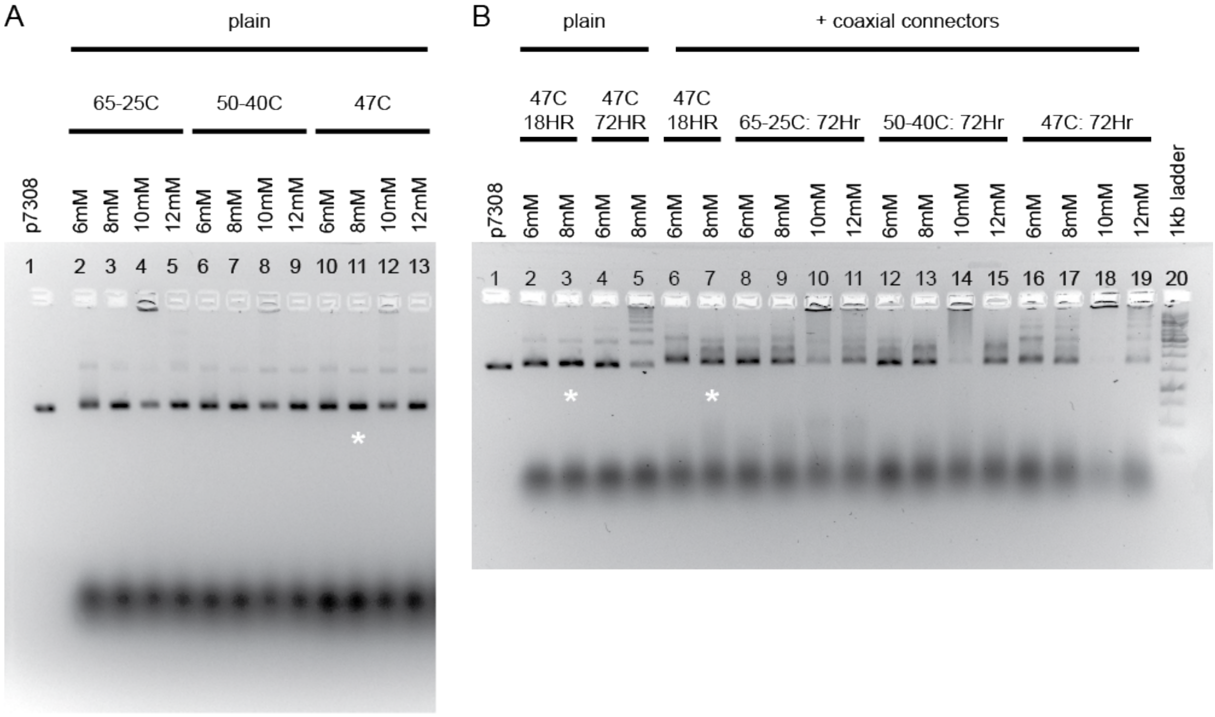

Supplementary Figure 13. 30-27 nm barrel folding optimisation.

Agarose gel electrophoresis of monomer folding optimisation. **A** Comparison of plain barrel with MgCl2 of 6, 8,10,12 mM, and 3 folding ramps for 18 hours: 65-25C, 50-40C and 47C. **B** Comparison of plain barrel (lanes 2-5) to barrels with coaxial connectors (lanes 8-19). MgCl2 concentrations of 6, 8,10,12 mM were compared as well as 4 folding ramps: 47C, 18 hours and 72 hours, 65-25C 72 hours, 65C 15 min + 50-40C 72 hours. Optimised conditions selected were 8 mM MgCl2, 47C 18 hours (* **A**: lane 11, **B**: lane 3 and 7).

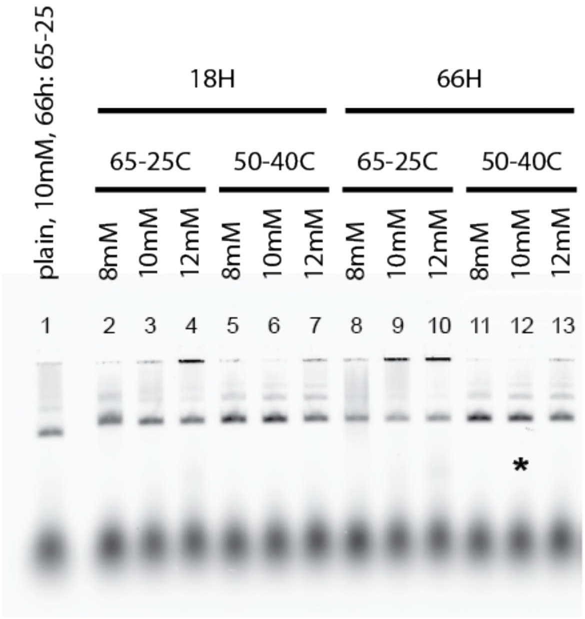

Supplementary Figure 14. 30-65 nm barrel folding optimisation

Agarose gel electrophoresis comparison of plain barrel with no pixels decorated (lane 1) to full set of outer 72 outer pixels (lanes 2-13). MgCl2 concentrations of 8,10,12 mM were compared as well as 4 folding ramps: 65-25C, 18 hours and 66 hours, 65C 15 min + 50-40C 18 hours or 66 hours. Optimised conditions selected were 10mM MgCl2, 65C 15 min + 50-40C 66 hours (* lane 12).

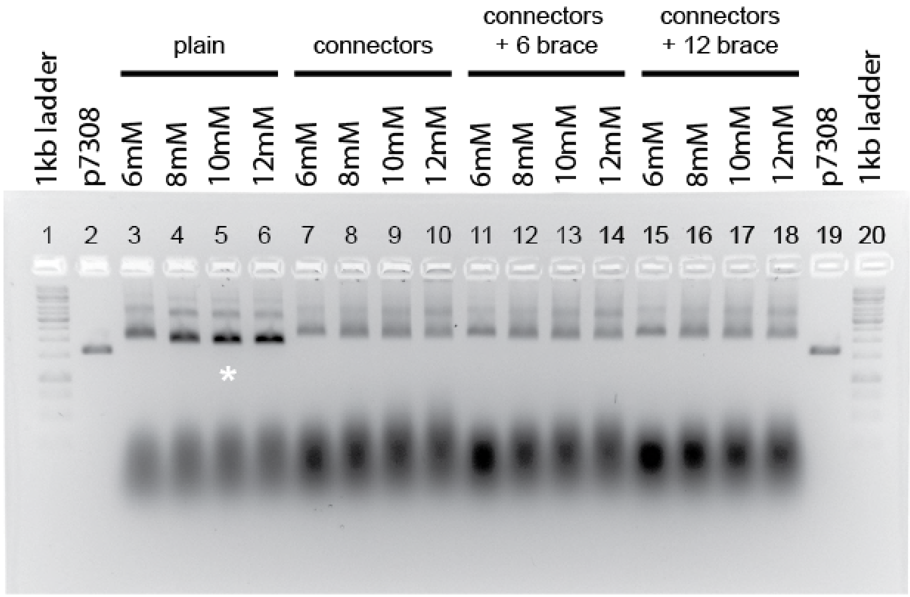

Supplementary Figure 15. 60-27 nm barrel folding optimisation

Agarose gel electrophoresis comparison of plain barrel (lanes 3-6) to barrels with: coaxial connectors (lanes 7-10), coaxial connectors plus 6 coaxial braces (lanes 11-14), or coaxial connectors plus 12 coaxial braces (lanes 15-18). MgCl2 concentrations of 6, 8,10,12 mM were compared for the folding ramp: 65-25C, 18 hours. Optimised condition selected for plain barrels is 10mM MgCl2 (* lane 5). For this connector design, monomers were significantly aggregated during folding (lanes 7-18). Additional connector design optimisation was performed to address this (Supplementary Figure 22).

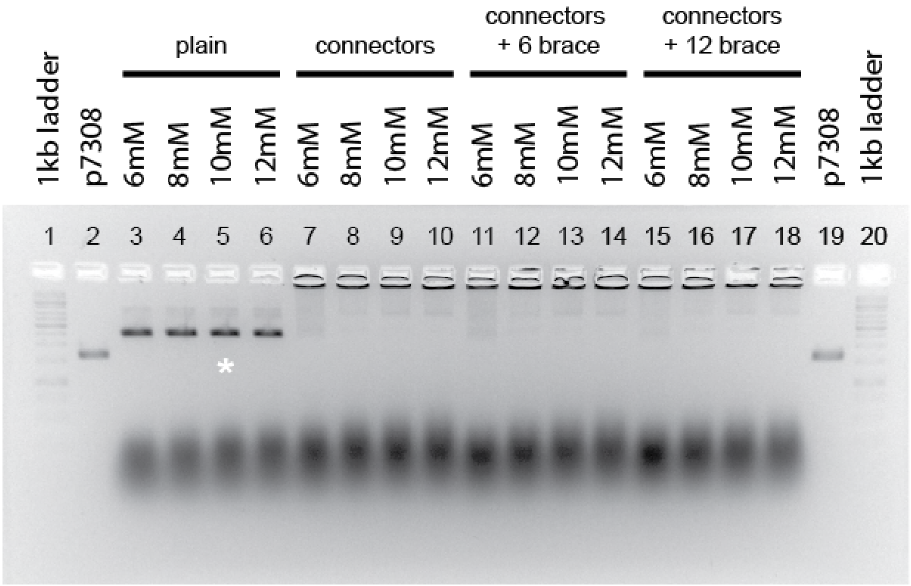

Supplementary Figure 16. 90-19 nm barrel folding optimisation

Agarose gel electrophoresis comparison of plain barrel (lanes 3-6) to barrels with: coaxial connectors (lanes 7-10), coaxial connectors plus 6 coaxial braces (lanes 11-14), or coaxial connectors plus 12 coaxial braces (lanes 15-18). MgCl2 concentrations of 6, 8,10,12 mM were compared for the folding ramp: 65-25C, 18 hours. Optimised conditions selected was 10mM MgCl2 (* lane 5). For this connector design ('plain', Supplementary Figure 22), monomers were almost entirely aggregated during folding. Additional connector design optimisation was performed to address this (Supplementary Figure 22).

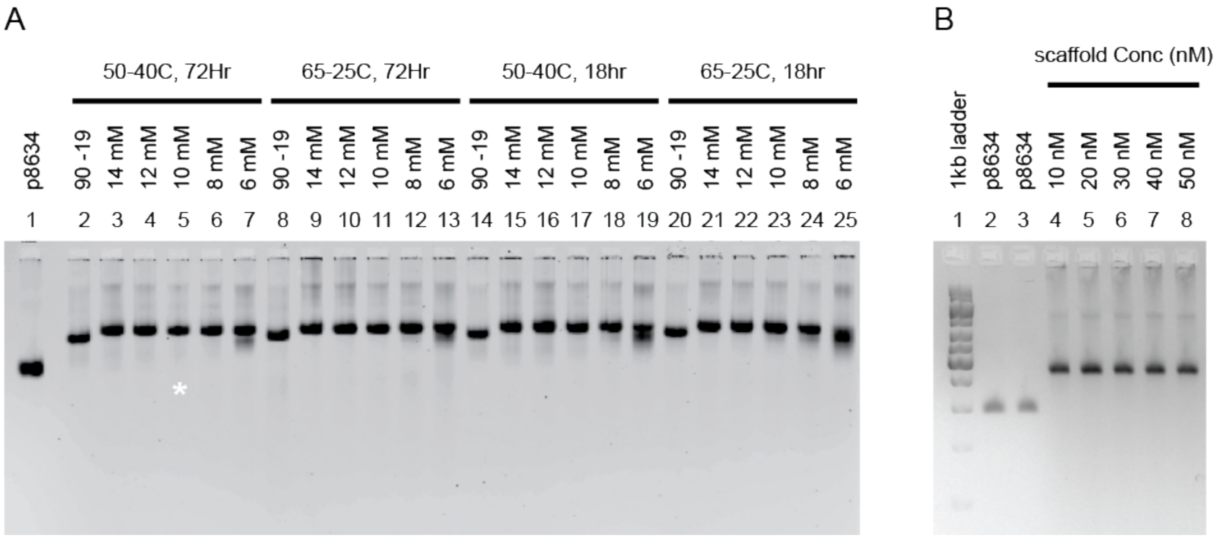

Supplementary Figure 17. 90-23 nm barrel folding optimisation

A Agarose gel electrophoresis comparison of plain barrel with MgCl2 of 6, 8,10,12,14 mM, and 2 folding ramps for 18 hours and 72 hours: 65-25C, 50-40C. Control lanes show 90-19 nm barrel, folded with 10 mM MgCl2 (lanes 1, 8, 14, 20), as shown in gel (iv). **B** Comparison of plain 90-23 nm barrel folded at different scaffold concentrations: 10, 20, 30, 40, 50 nM, with 10mM MgCl2 and 65-25C 18Hr ramp. Scaffold concentrations are normalised to 200 pmol/lane. Yield is independent of scaffold concentration in this range. Optimised conditions selected were 10 mM MgCl2, 50-40C 72 hours (* lane 5).

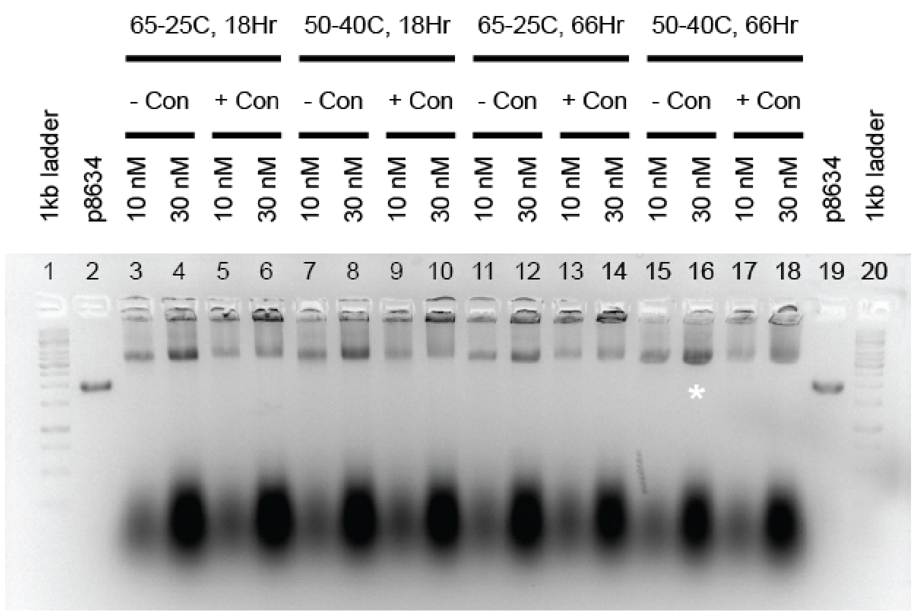

Supplementary Figure 18. 90-23 nm barrel folding optimisation, coaxial

Agarose gel electrophoresis comparison of 90-23 nm barrels with coaxial ‘plugs’, folded at 10 mM MgCl2 with 2 folding ramps: 18 hours and 66 hours: 65-25C, 50-40C. For each ramp, barrels are folded in 1-step with (+ Con) or without (- Con) the interface connector strands, and at two scaffold concentrations (10 nM and 30 nM). For all scaffold and ramp conditions, addition of connector strands (+ Con) into monomer folding resulted in lower yield than samples folded without connector strands (-Con). Optimised conditions selected were 10 mM MgCl2, 5-40C 66 hours, 30 nM scaffold without connector strands (* lane 16). Connector strands were added during assembly of barrel polymers.

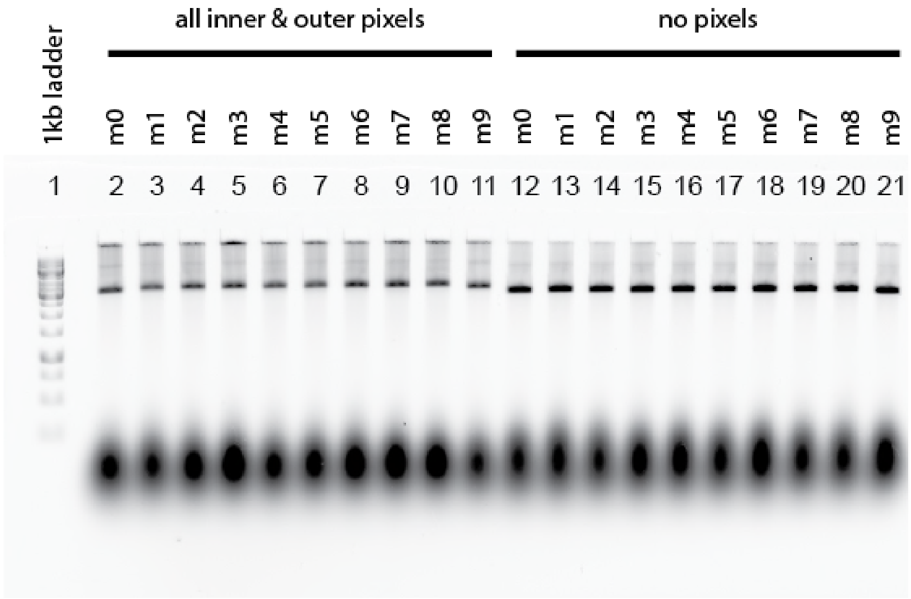

Supplementary Figure 19. 90-23 nm barrel folding optimisation, m0-m9

Agarose gel electrophoresis of 90-23 nm barrel monomers folded with coaxial connectors, for 10 orthogonal connector sets. 10 different coaxial connector sequence sets were designed for orthogonal assembly of the 10 monomers m0-m9 into the 90 nm decamer design (Figure 4). Monomer folding yield for the different connector sets is shown for two pixel designs: no pixels (lanes 12 - 21), and with every inner and outer pixel decorated with a 10-nt DNA PAINT docking site to give a total of 216 docking sites for monomers m1-m8 (lanes 3-10) and 198 for m0/m9 (lanes 2,11). Monomer folding yield for fully decorated monomers is lower than undecorated. Monomer yields are similar across m0-m9 for a given design. Folding conditions are 10mM MgCl2, 50-40C 66 Hrs, 30 nM scaffold, without connector strands.

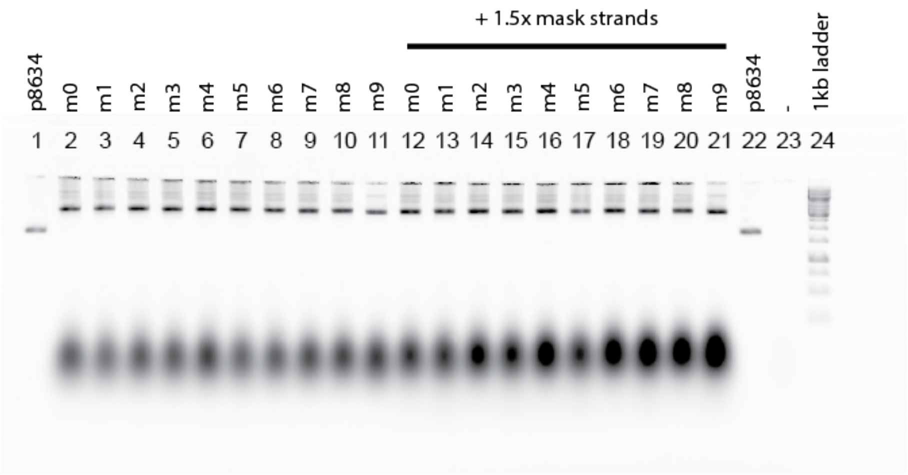

Supplementary Figure 20. 90-23 nm barrel folding optimisation, mask strands
Agarose gel electrophoresis folding yield analysis of the pixel decorated monomers was found to be lower than the undecorated monomer (Supplementary Figure 19). Masking strands (Supplementary Figure 10) were included in monomer folding at 1.5x excess to total pixel staple concentration, to improve folding yield. Monomer folding yield for the different connector sets is shown here for barrels with every inner and outer pixel decorated with a 10-nt DNA PAINT docking site to give a total of 198-216 docking sites per monomer, with (lanes 12-21) and without (lanes 2 - 11) masking strands. Masking strands did not improve yield of monomer folding significantly. Folding conditions are 10mM MgCl2, 50-40C 66 Hrs, 30 nM scaffold without connector strands.

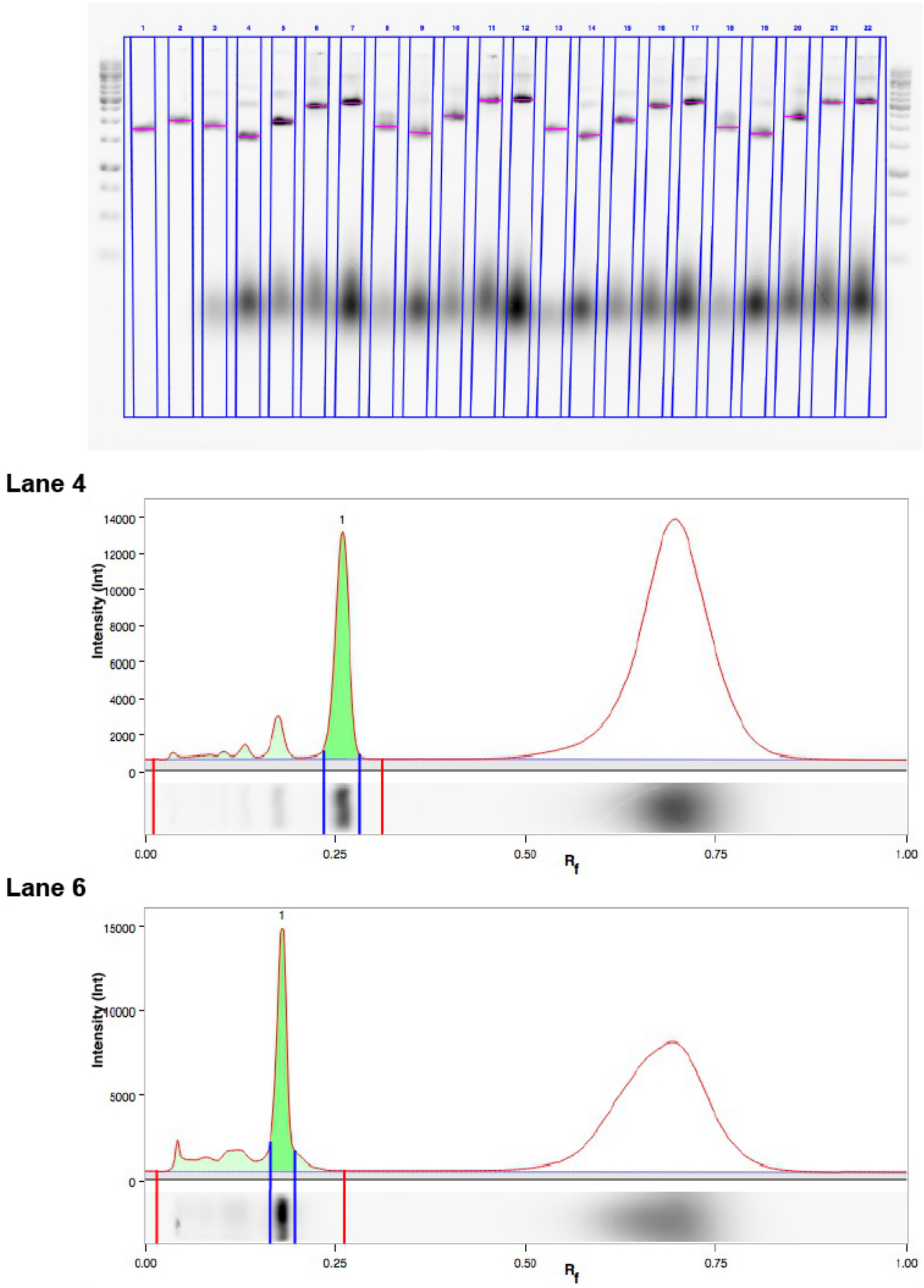

Supplementary Figure 21. Quantification of monomer folding yields

Monomer folding yields were calculated from agarose gels using automated lane detection and background subtraction (top panel). Example lane profiles are shown for Lane 4 (30-65 nm barrel, plain design, middle panel) and Lane 6 (90-19 nm barrel, plain design, bottom panel) For each lane, the integrated monomer band is detected (dark green peak, boundaries indicated by blue vertical lines), and compared to the total integrated signal for all high molecule weight products (light green area, boundaries indicated by red vertical lines). Monomer yield is calculated as the % of the monomer band to total high molecular weight products in Supplementary Table 1.

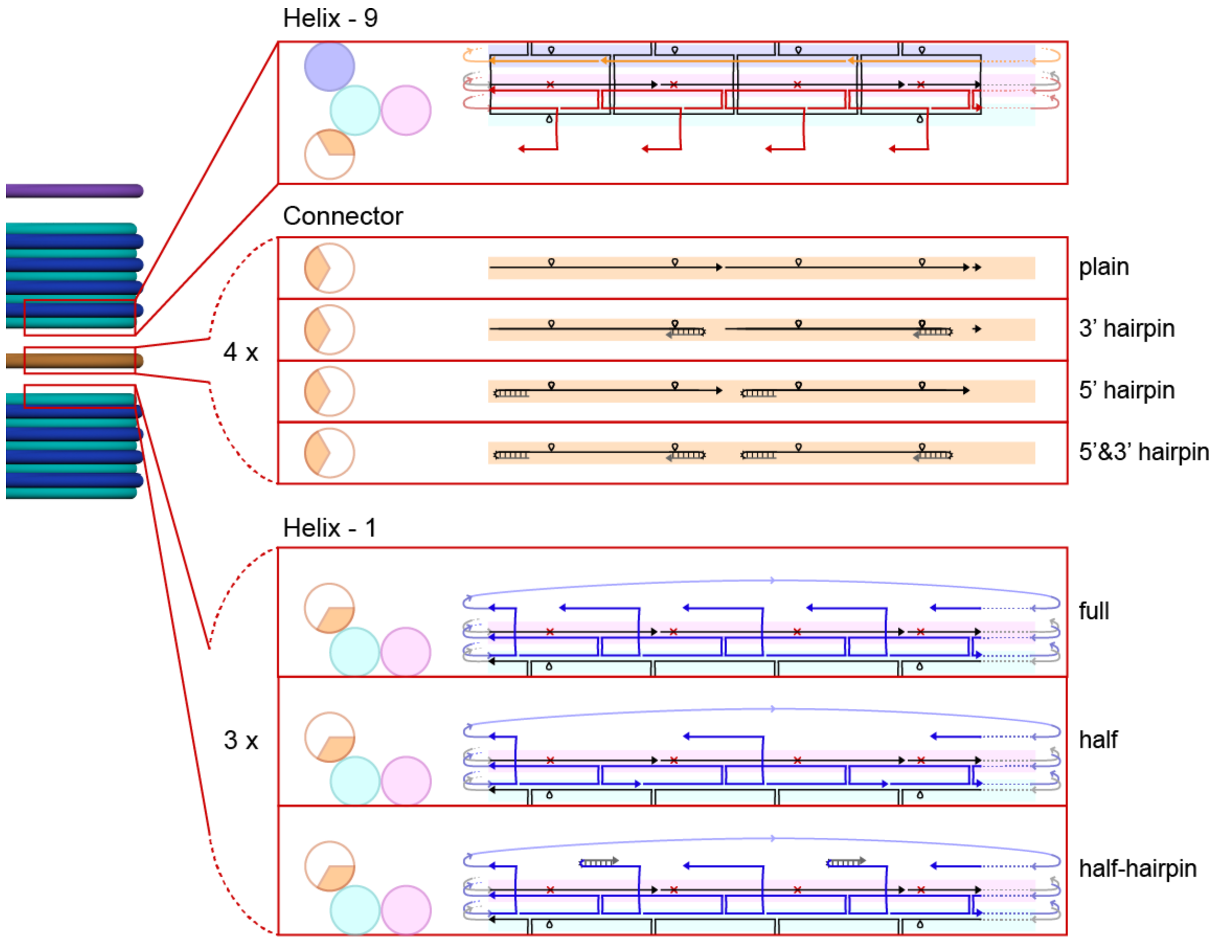

Supplementary Figure 22. Schematic of coaxial interface variants

Each interface consists of plugs found on the bottom of the upper monomer (Helix - 9) and on the top of the lower monomer (Helix - 1), as well as short scaffold-parity strands to link them (Connector). For the 90-19 nm barrel 4 different designs of the connector strands were compared: 1) plain, 2) with 6-nt 3' hairpin domains, 3) with 6-nt 5' hairpin domains, and 4) with both 6-nt 5' and 3' hairpin domains. 3 different designs of helix-1 were compared: 1) full 36 plug set, 2) half alternating 18 plug set, and 3) half-hairpin a plug set with alternating plain and 6-nt 3' hairpin domains. For each design motif, 4 different sequence compositions were tested, all with 50% GC content. These sets varied in the ratio of G:C, 7:3 ('plugs' are G-rich), 3:7 'connectors are G-rich), 8:2 and 2:8. Two types of assembly process were also compared, monomers folded directly with the connector strands (1-step), and monomers folded without connector strands and then incubated with after (2-ste'). In total, this gave 96 possible combinations. A summary of key results is shown in Supplementary Figures 23-25.

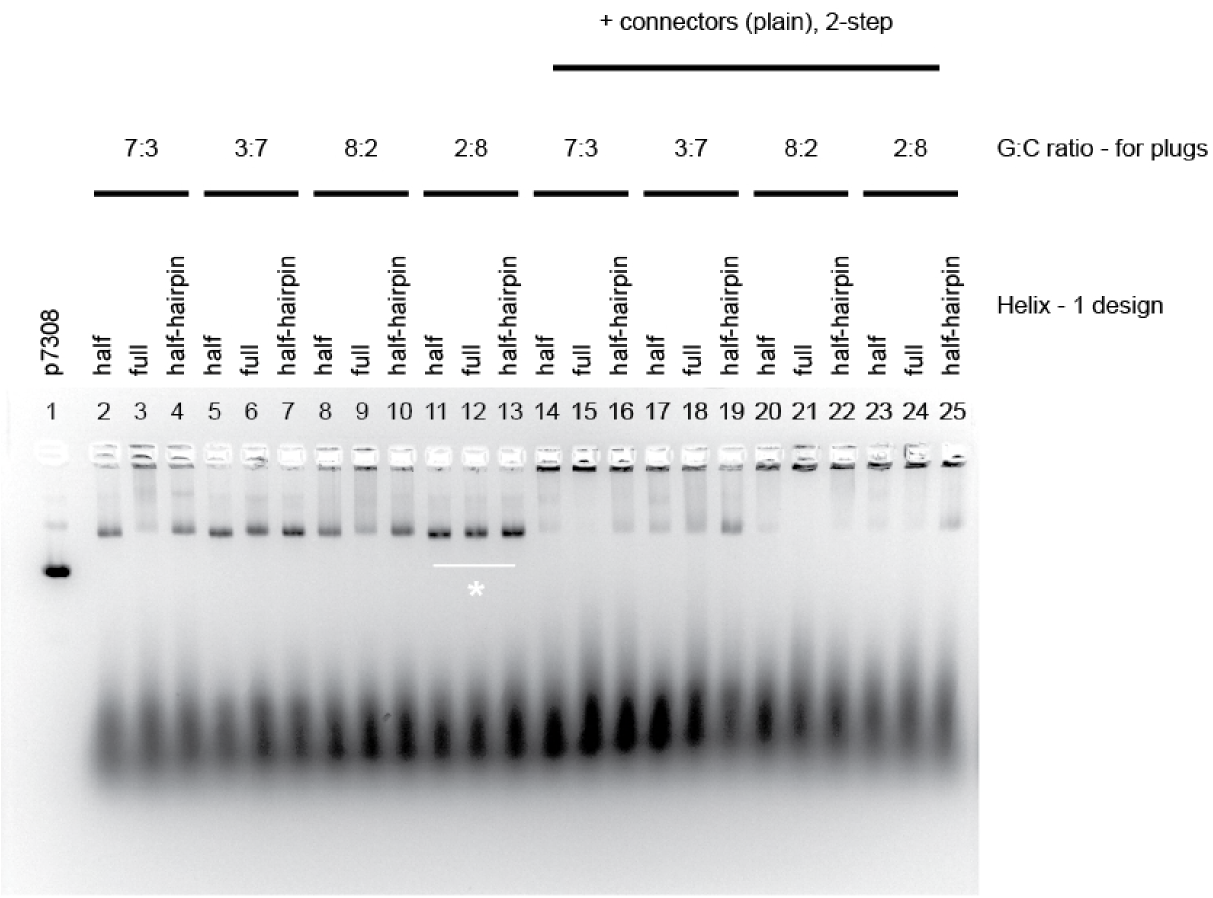

Supplementary Figure 23. 90-19 nm coaxial optimisation, sequence variants

Agarose gel electrophoresis of barrel monomers folded without the connector strands for 3 different plug designs: half, full, and half-hairpin. Four different sequence variants were compared, all with GC 50%, and with G:C ratios of 7:3 (plugs are G-rich), 3:7 (connectors are G-rich), 8:2 and 2:8. Connector strands were added in a two-step assembly process, by incubating with pre-folded monomers at 10x excess for 16 hours at 25C. Monomers with fewer plugs (lanes, 2,5,8,11) or with hairpin plugs (lanes 4,7,10,13) were less prone to aggregation than the full sets (lanes 3,6,9,12). G-rich connector sets (lanes 5-7 or lanes 11-13) were less prone to aggregation than G-rich plugs (lanes 2-4 or lanes 8-10). A G:C ratio for plugs of 2:8 was found to give the highest monomer yield for monomer folding without connectors (* lanes 11-13). However, on incubation with plain connectors, all monomers aggregated (lanes 14-25). All monomers were folded at 10mM MgCl2, from 65-25C over 18 hours.

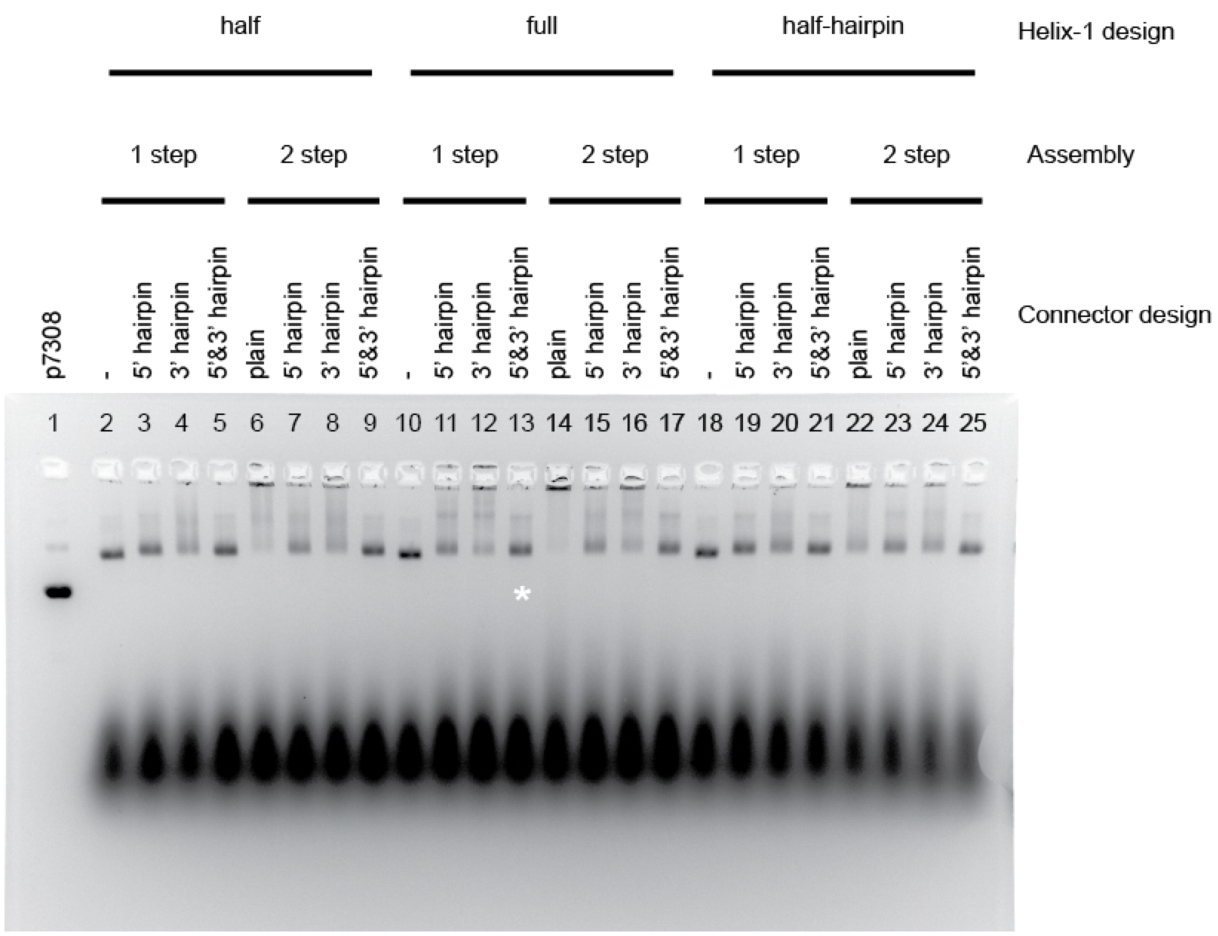

Supplementary Figure 24. 90-19 nm coaxial optimisation, connection design variants

For the optimal sequence design of 50% GC, G:C ratio of 2:8 for plugs (G-rich connectors), a comparison was made of different connector and plug designs, as depicted in schematic in (i). For all 3 Helix-1 designs, monomers fold well without connectors (lanes 2,10,18), but aggregate almost entirely on addition of plain connectors (lanes 6,14,22), this is in agreement with results in gel (ii) above. For all helix-1 designs, addition of hairpin domains to the connector strands decreases aggregation and improves yield. Addition of both 5' and 3' hairpin domains to connectors results in the highest yields for monomers after addition of connectors for either assembly process (lanes 5,9,13,17,21,25). For polymerisation experiments, conditions used were: helix-1 full, 1-step assembly, connector helix 5' & 3' hairpin (* lane 13). All monomers were folded at 10mM MgCl2, from 65-25C over 18 hours. For two step assembly connector strands were incubated with pre-folded monomers at 10x excess for 16 hours at 25C.

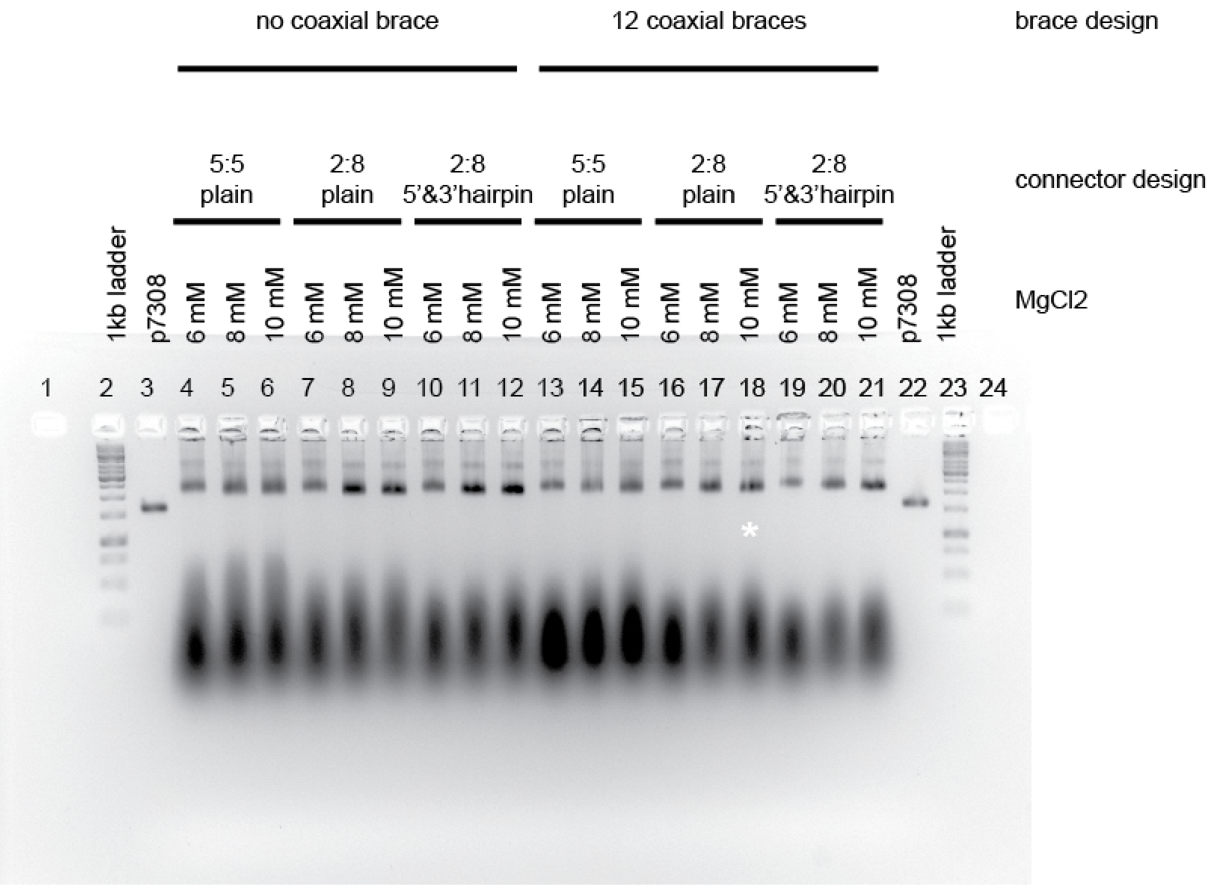

Supplementary Figure 25. 60-27 nm coaxial optimisation, connection and sequence variants

Using the same optimal helix-1 design as for the 90-19 nm barrel, a comparison was made of different connector designs and sequence variants for the 60-27 barrel. Monomers were folded in 1-step with connector strands, at varying MgCl2 (6,8,10 mM) from 65-25C over 18 hours. Two different sequence sets were compared with GC 50% and G:C ratio of 1:1 and G:C ratio of 2:8 (G-rich connectors). Two connector designs were compared, plain and with both 5' & 3' hairpin domains. Similarly to the 90-19 nm barrel (iii), 10mM MgCl2 and G:C of 2:8 was optimal (lanes 9,12,18,21). In this case little difference was seen between the connector designs of plain (lanes 9 and 18) and 5'&3' hairpin (lanes 12 and 21). Plain connectors, 2:8 G:C, with 12 coaxial braces were selected as the optimal condition (* lane 18).

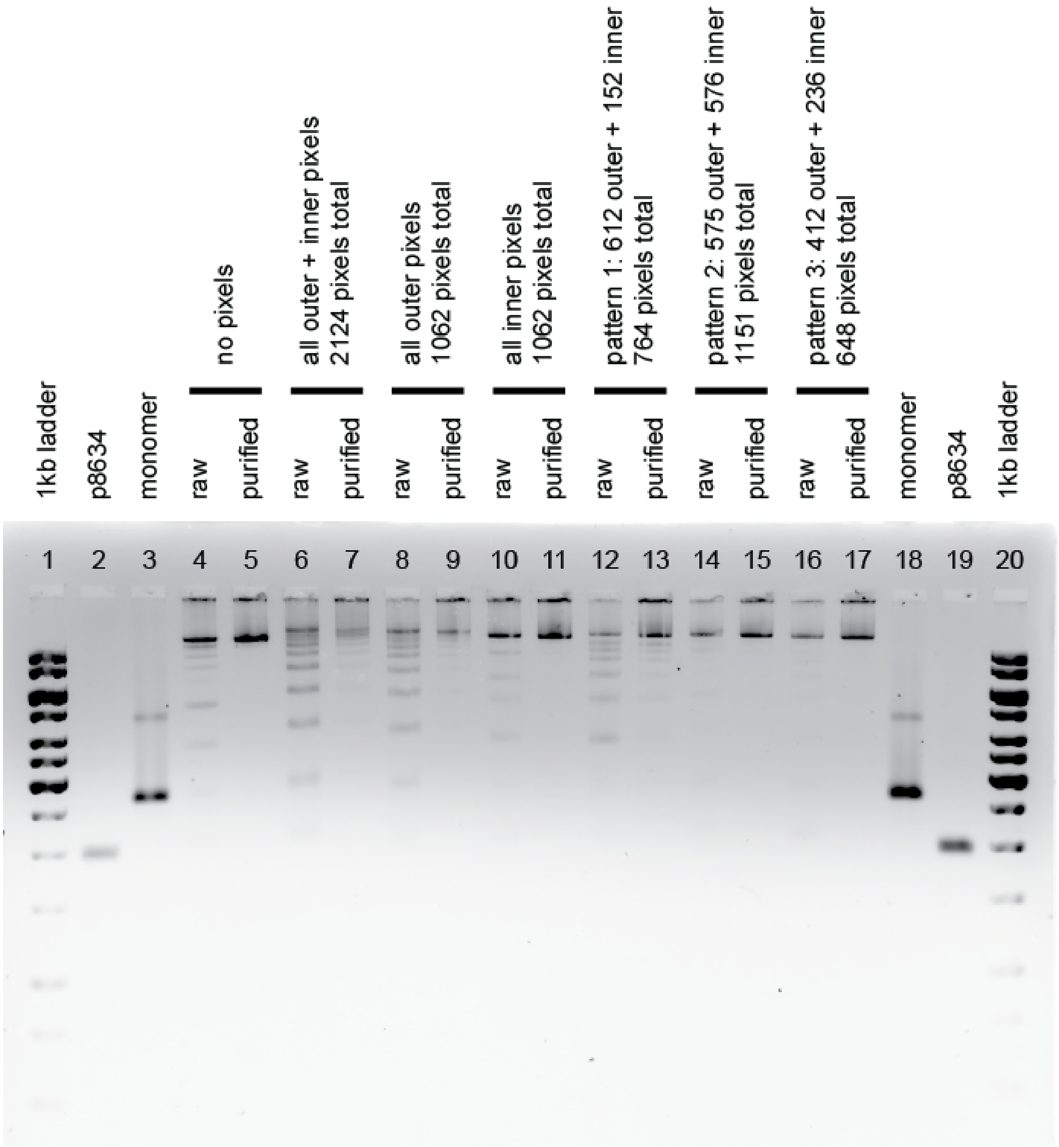

Supplementary Figure 26. 90-23 nm decamer gel yields for different pixel designs

Comparison of 90-23 nm decamer assembly yields for different pixel designs, showing both raw and purified decamers. All designs have inner pixels of the bottom helix of m9 decorated with 18 5'-biotin modified staples. All decamer samples are normalised to 0.05 pmol per lane (lanes 4-17). Pattern 1 is used for DNA-PAINT imaging in Figure 4 (lane 13). All samples with outer pixels are assembled with 1.5x mask strands present (lanes 6-9 and 12-17). (1.5 % Agarose gel)

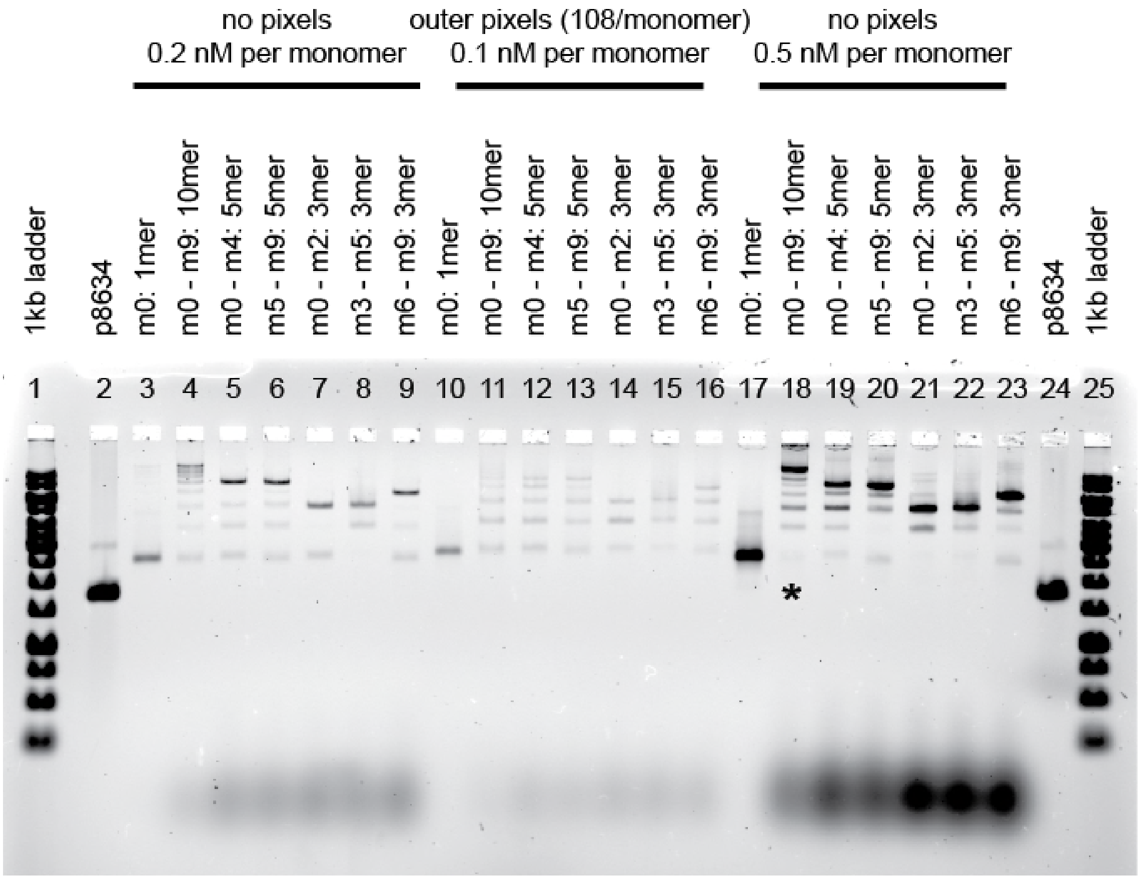

Supplementary Figure 27. 90-23 nm barrel 3/5/10-mer assembly

Monomers (lanes 3, 10, 17), 3-mers (lanes 7-9, 14-16, 21-23), 5-mers (lanes 5-6, 12-13, 19-20), and 10-mers (lanes 4, 11, 18). Assembly conditions are equimolar concentrations of monomers, 100x excess of connectors strands, 20mM MgCl2, and annealing from 40-20C over 12 hours. Comparison was made of barrels with no pixels decorated (lanes 3-9) to barrels with all outer pixels decorated with 108 pixels per monomer (lanes 10-16). Barrels with outer pixel decorations show lower assembly yields. For barrels with no pixels, comparison is made of assembly monomer concentrations of 0.2 nM (lanes 2-9) and 0.5 nM (lanes 17-23). Yield of larger assemblies increases with higher monomer concentration. Optimal decamer yield is obtained for no pixels 0.5 nM monomers (* lane 18). (1.5 % Agarose gel)

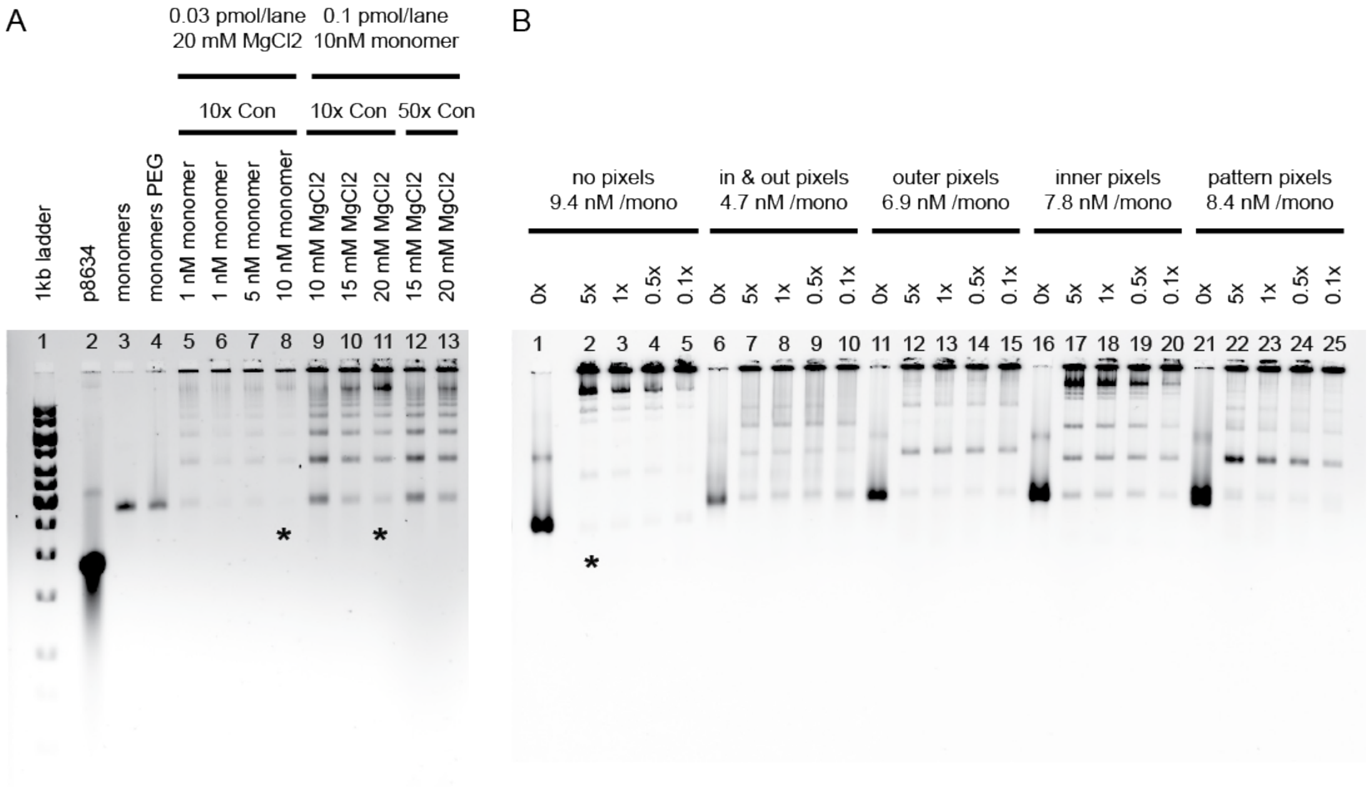

Supplementary Figure 28. 90-23 nm decamer optimisation of assembly conditions

A range of conditions were optimised for decamer assembly including monomer concentration, excess of connector strands, and MgCl2 concentration. **A** All 10 monomers were pooled (lane 3), and PEG precipitated (lane 4). Comparison is made between monomer concentrations of 1, 5, 10 nM, with 10x connector excess and 20 mM MgCl2 (lanes 5-8), and of 10, 15, 20 mM MgCl2 for 10 nM monomer concentration and either 10x connector excess (lanes 9-11) or 50x connector excess (lanes 12-13). Optimal conditions are 10x connector excess, 10nM monomer concentration, and 20 mM MgCl2 (* lanes 8 and 11). **B** Decamer assembly was compared for different pixel designs: no pixels (lanes 1-5), all inner and outer pixels (lanes 6-10), all outer pixels (lanes 11-15), all inner pixels (lanes 16-20), and for pixel pattern for design in Figure 4 (lanes 21-25). Connector strand excess was varied from 0.1x to 5x, with 20 mM MgCl2. Decamer yield is lower for designs with outer pixels (lanes 6-10, 11-15 and 21-25), which are significantly aggregated. Decamer yield decreases with connector excess below 5x. Best conditions for B are for ‘no pixel’ design, 5x connector excess (lane 2). All decamers were annealed 40-20C 12hrs. Overall optimal conditions for decamer assembly are 10 nM monomer concentration, 10x connector excess, 20 mM MgCl2, annealing 40-20C 12hrs. (1.5 % Agarose gel)

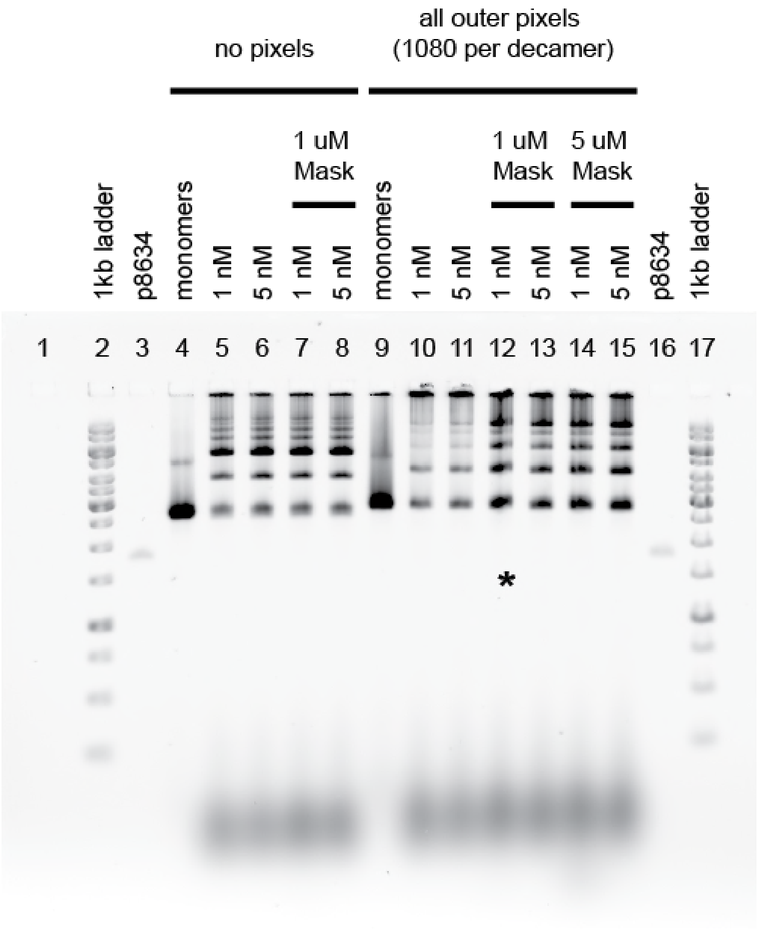

Supplementary Figure 29. 90-23 nm decamer optimisation of pixel mask strands

Mask strands are designed to be complementary to DNA PAINT docking site 'pixels' (Supplementary Figure 10).The addition of mask strands at 1 uM and 5 uM to decamer assembly was compared for 'no pixel' and 'all outer pixel designs (1062 pixels per decamer), for monomer concentrations of 1 nM and 5 nM. MgCl2 is 20 mM, and all decamers re annealed 40-20C 12 hrs. As expected, mask strands have no effect on assembly of 'no pixel' design (lanes 5-8). For 'all outer pixels' design, decamer assembly leads to aggregation (lanes 10-11). Addition of mask strands (lanes 12 - 15) reduces aggregation and increases yield of decamer. There is no significant different between addition of Mask at 1 uM (lanes 12-13) and 5uM (lanes 14-15), so mask strands were incorporated at 1-1.5x excess (* lane 12) for future assembly reactions. (1.5 % Agarose gel)

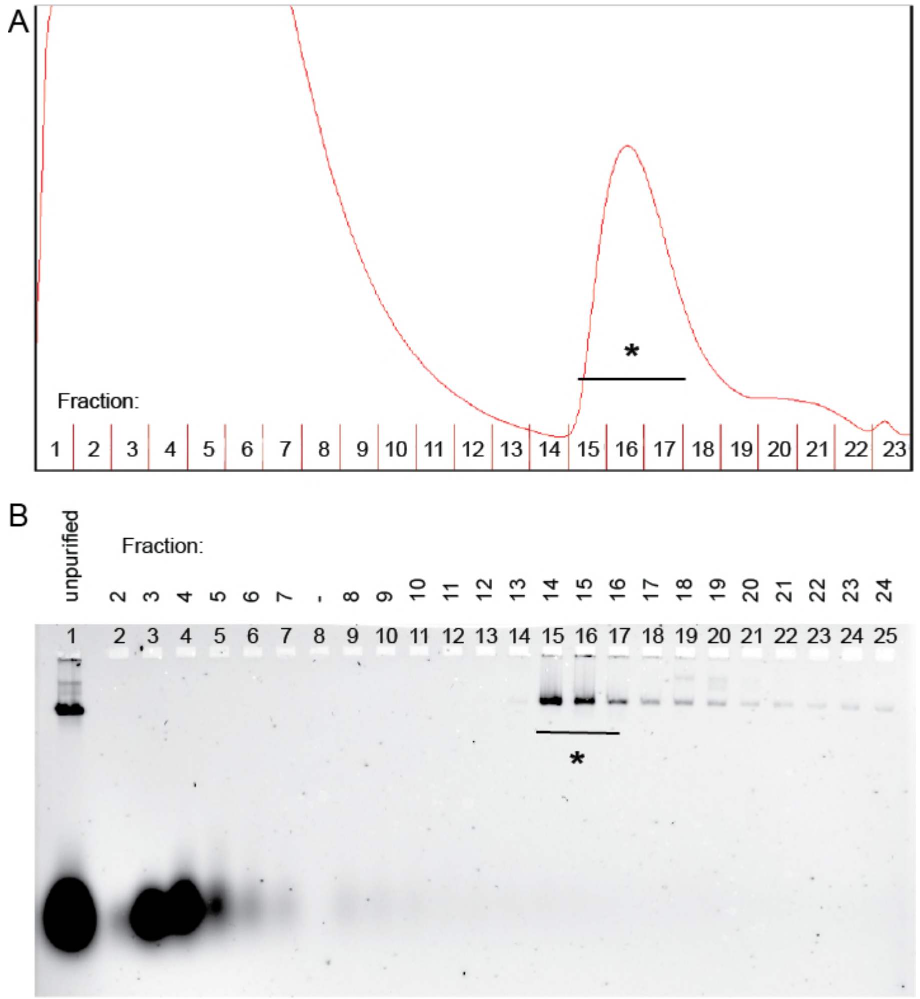

Supplementary Figure 30. 90-23 nm monomer glycerol gradient purification

A representative UV fractionation trace (A) and agarose gel of resulting fractions (B) are shown for m5 of no-pixel design. Fractions 14-16 (*lanes 15-17) were combined as purified monomer.

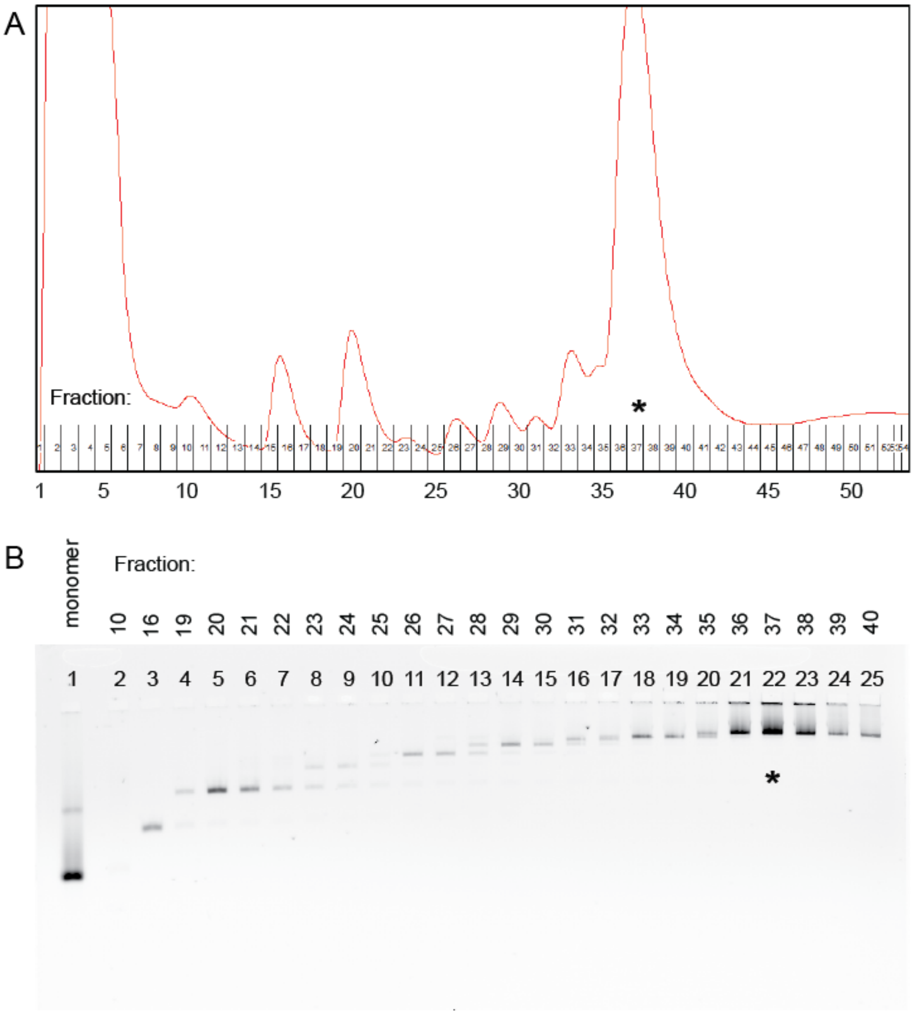

Supplementary Figure 31. 90-23 nm decamer glycerol gradient purification

Assembly of monomers into decamers was incomplete (Supplementary Figures 26-29), and glycerol gradient ultracentrifugation was used to purify complete decamers for DNA-PAINT experiments. A representative UV fractionation trace (A) and agarose gel of resulting fractions (B) are shown for no-pixel decamer design. Fractions 37 (*lane 22) was selected as purified decamer for TEM and DNA-PAINT imaging experiments and gel analysis in Supplementary Figure 26.

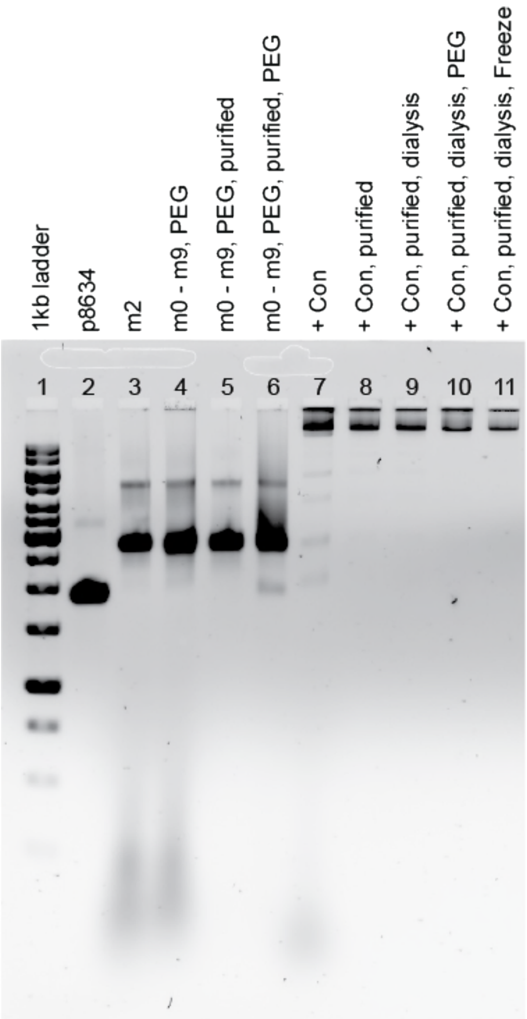

Supplementary Figure 32. 90-23 nm decamer assembly process

Sequential steps of the full 90-23 nm decamer assembly process are shown for a representative design, ‘10 stripe’, which consists of 1 helix of 18 inner pixels per monomer, to give a total of 180 inner pixels. Monomers m0-m9 were folded in separate reactions (lane 3), then pooled and PEG precipitated (lane 4), followed by purification using glycerol gradient ultracentrifugation (lane 5) and a second PEG precipitation (lane 6). Decamers were assembled from monomer pool by addition of connector strands at 10x excess to total monomer concentration, 20 mM MgCl2, and annealing 40-20C over 12 hours (lane 7). Decamers were glycerol gradient purified (lane 8), followed by dialysis to remove glycerol from purified sample (lane 9). Optional steps are a final PEG precipitation to increase decamer concentration (lane 10), or freezing of samples at -20C (lane 11).

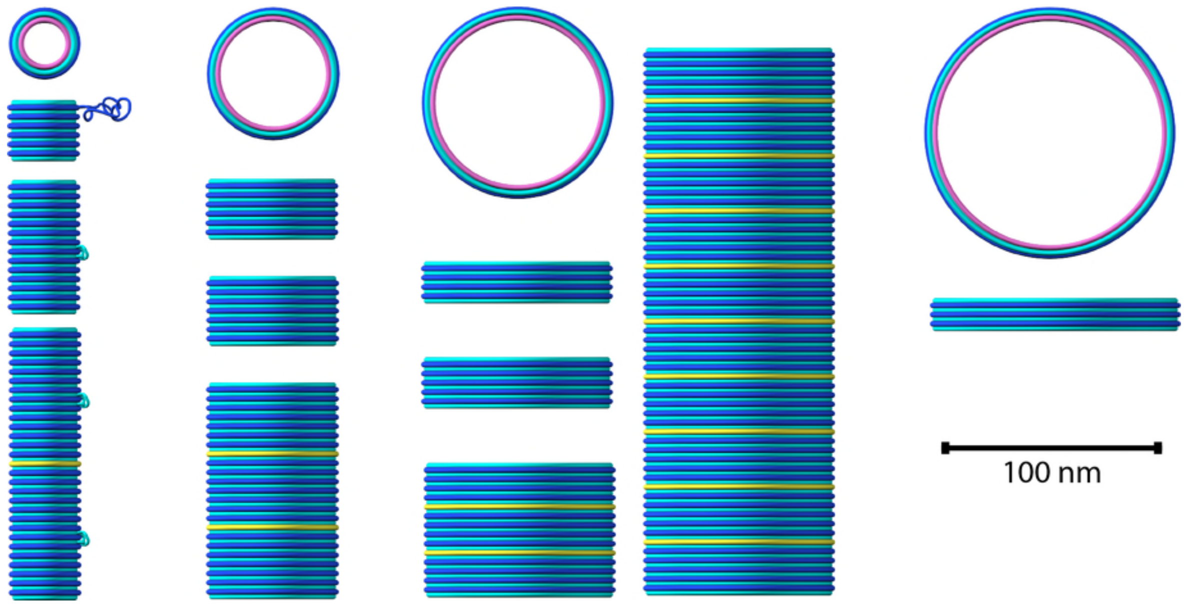

Supplementary Figure 33. 3D models of DNA barrel monomers and controlled multimers

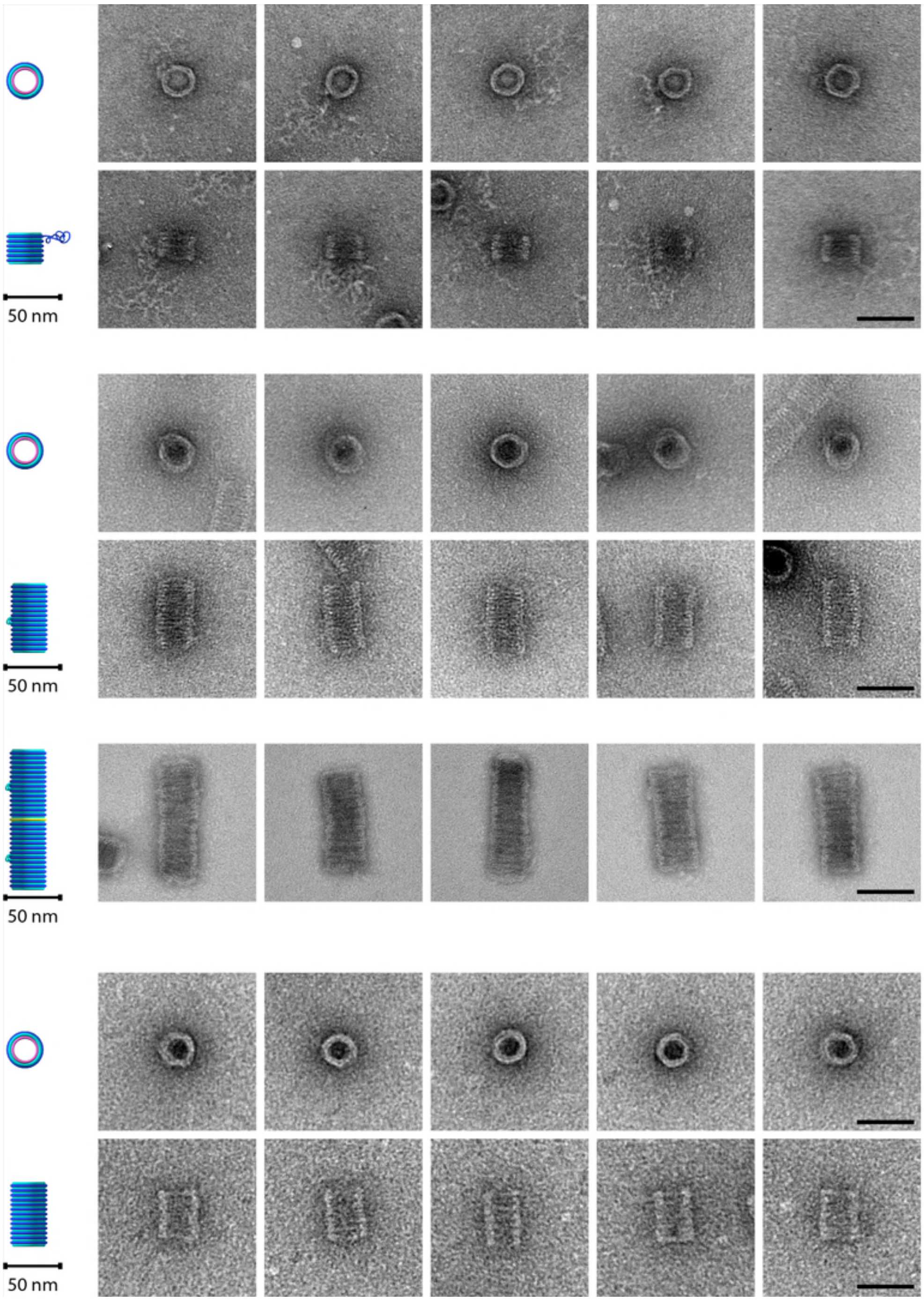

Supplementary Figure 34. Cropped TEMs, 30-27, 30-65, 30-131, and 30-61 nm.

Diameter = 30 nm, height = 27 nm, 65 nm, 131 nm (dimer), 61 nm. All same scale. Scale bars 50 nm

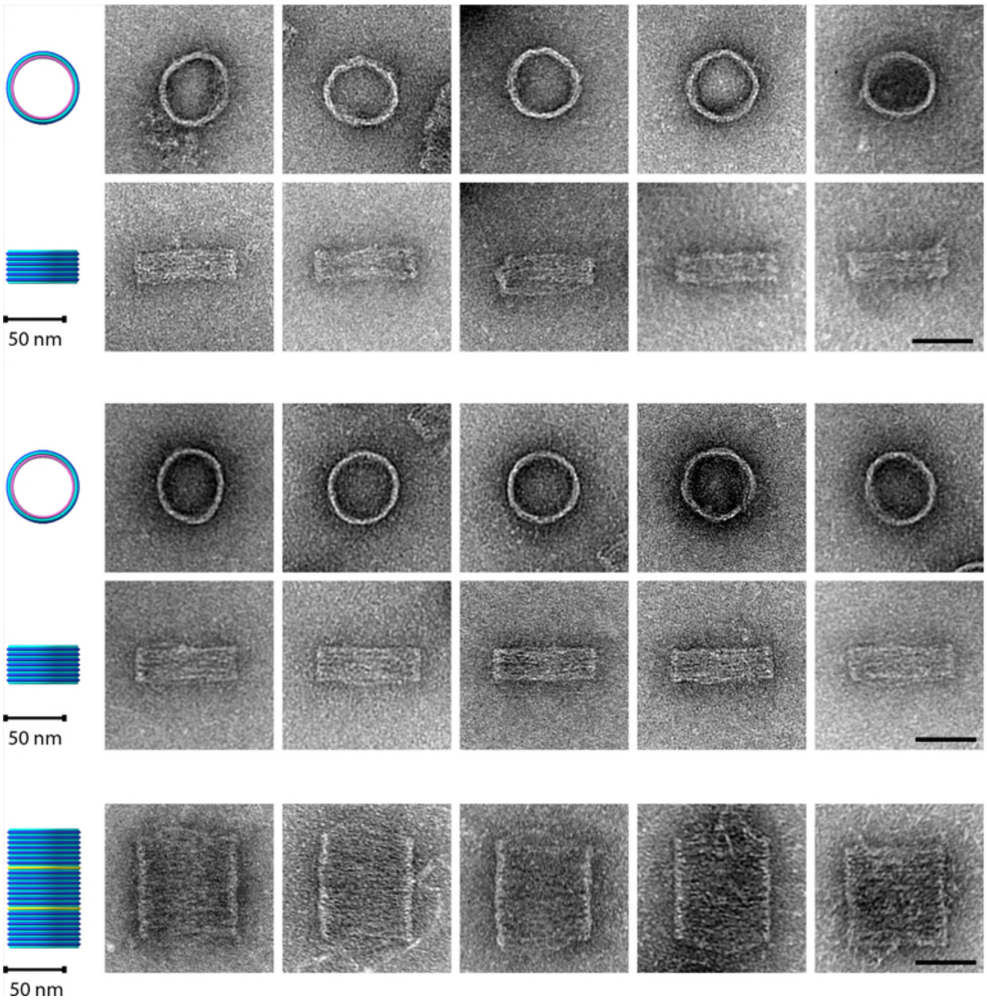

Supplementary Figure 35. Cropped TEMs, 60-27, 60-32, and 60-98 nm.
Diameter = 60 nm, height = 27 nm, 32 nm, 98 nm (trimer). All same scale. Scale bars 50 nm

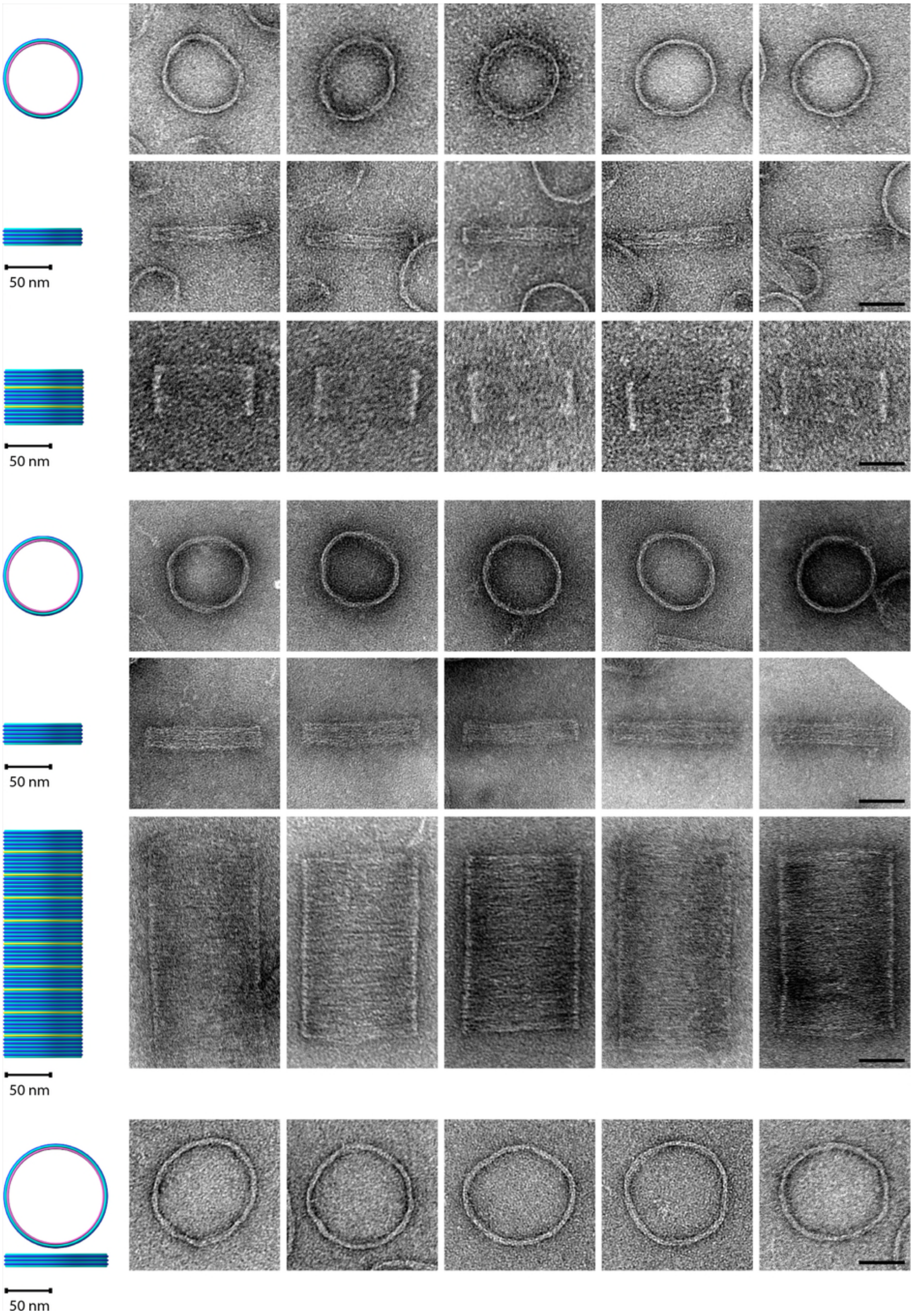

Supplementary Figure 36. Cropped TEMs, 90-19, 90-61 (3-mer), 90-23, 90-248 (10-mer), 120-15 nm

Diameter = 90 nm, height = 19 nm, 61 nm (trimer), 23 nm, 248 nm (10-mer)

Diameter = 120 nm, height = 15 nm

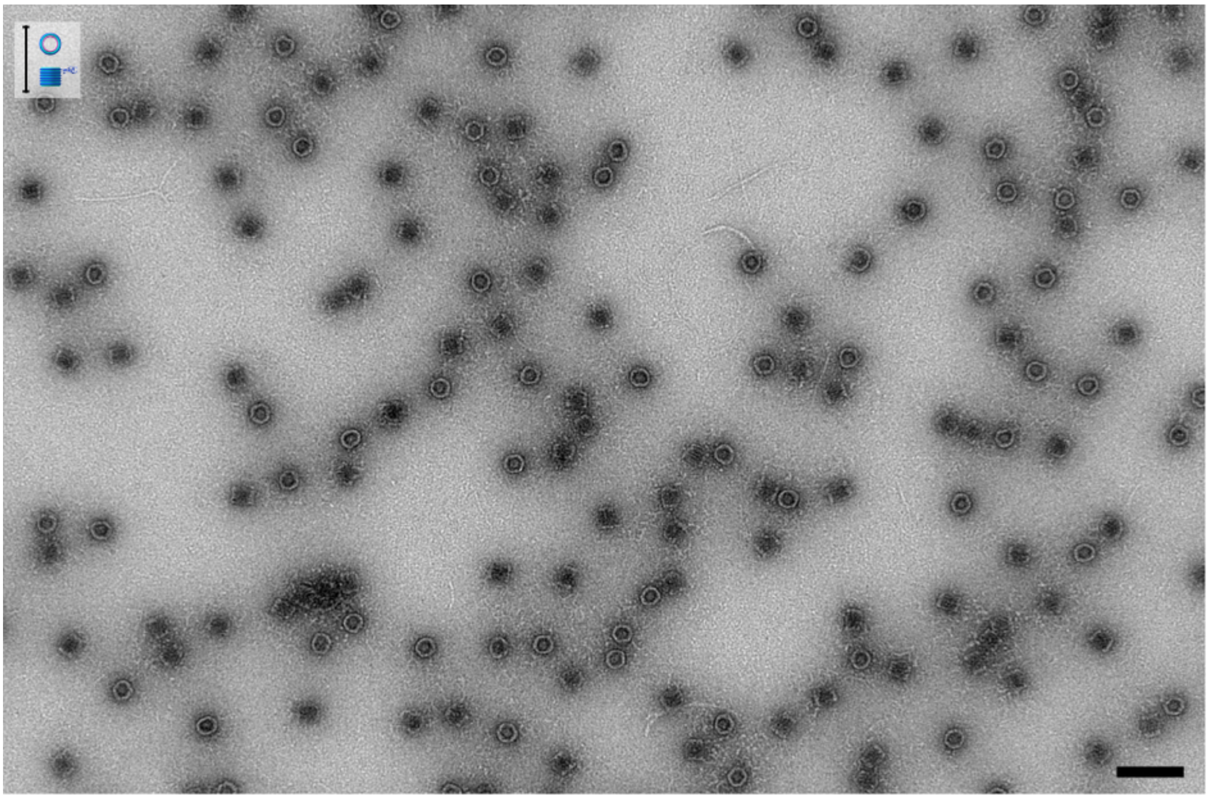

Supplementary Figure 37. Field-of-view TEM 30-27 nm barrel
Scale 100 nm.

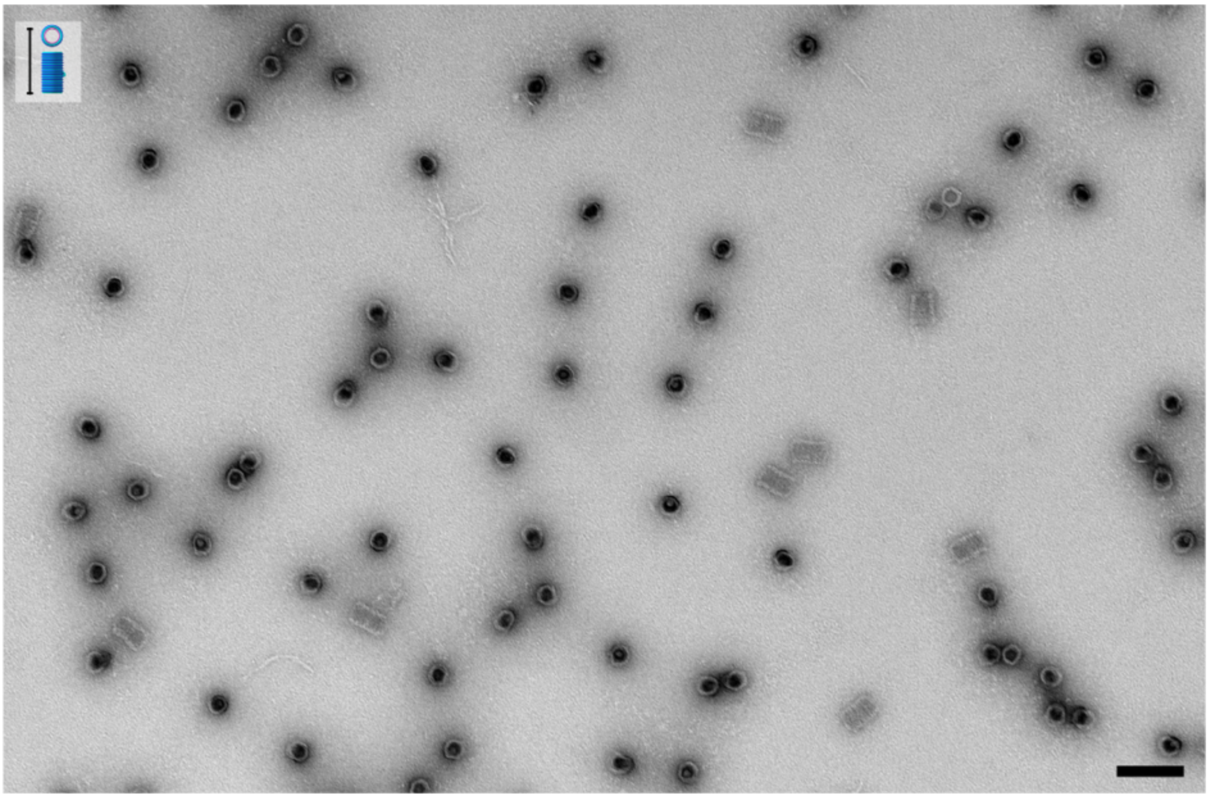

Supplementary Figure 38. Field-of-view TEM 30-65 nm barrel
Scale 100 nm.

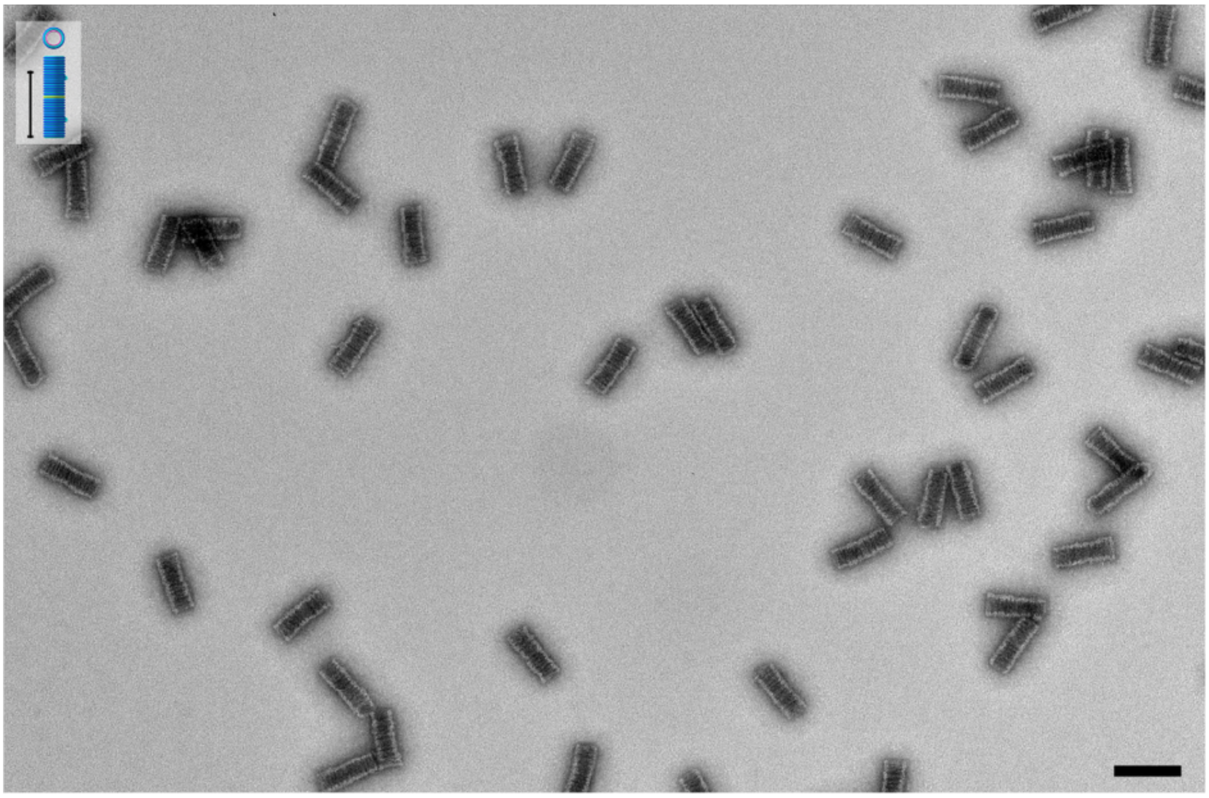

Supplementary Figure 39. Field-of-view TEM 30-65 nm barrel dimer (height = 131 nm)
Scale 100 nm.

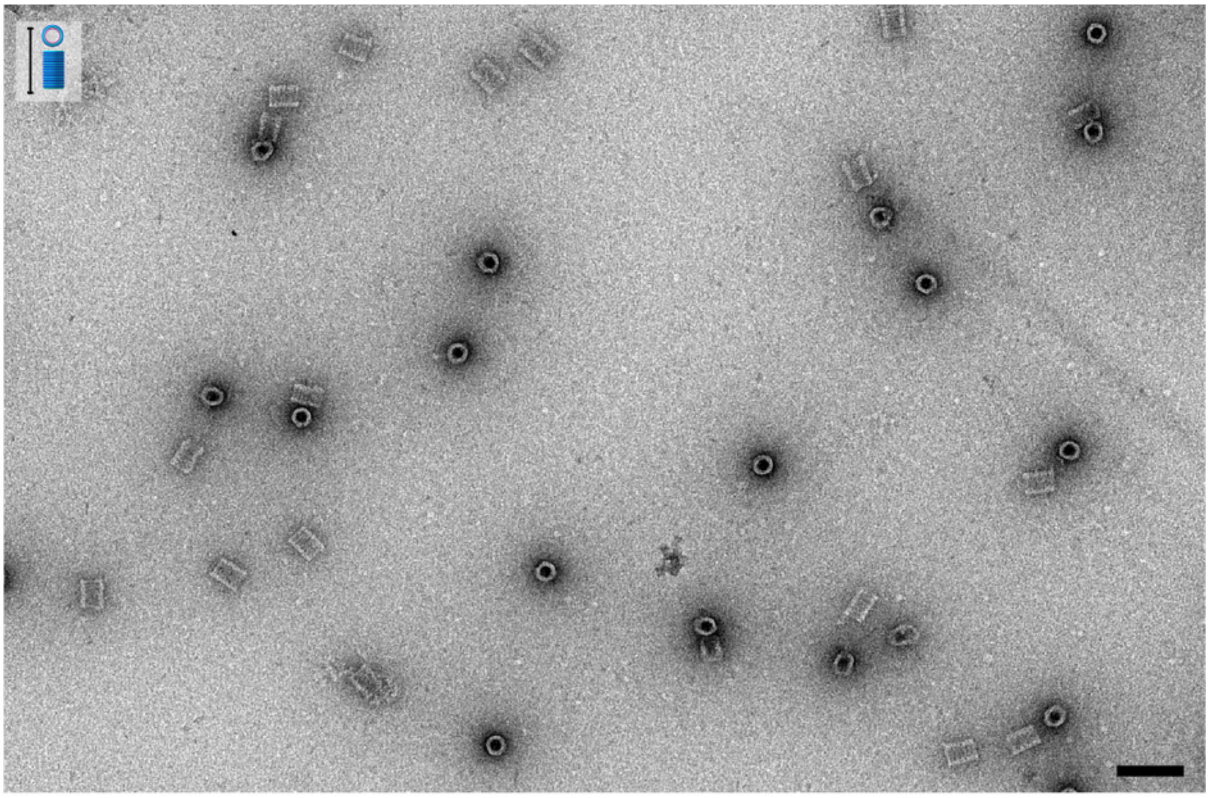

Supplementary Figure 40. Field-of-view TEM 30-61 nm barrel
Scale 100 nm.

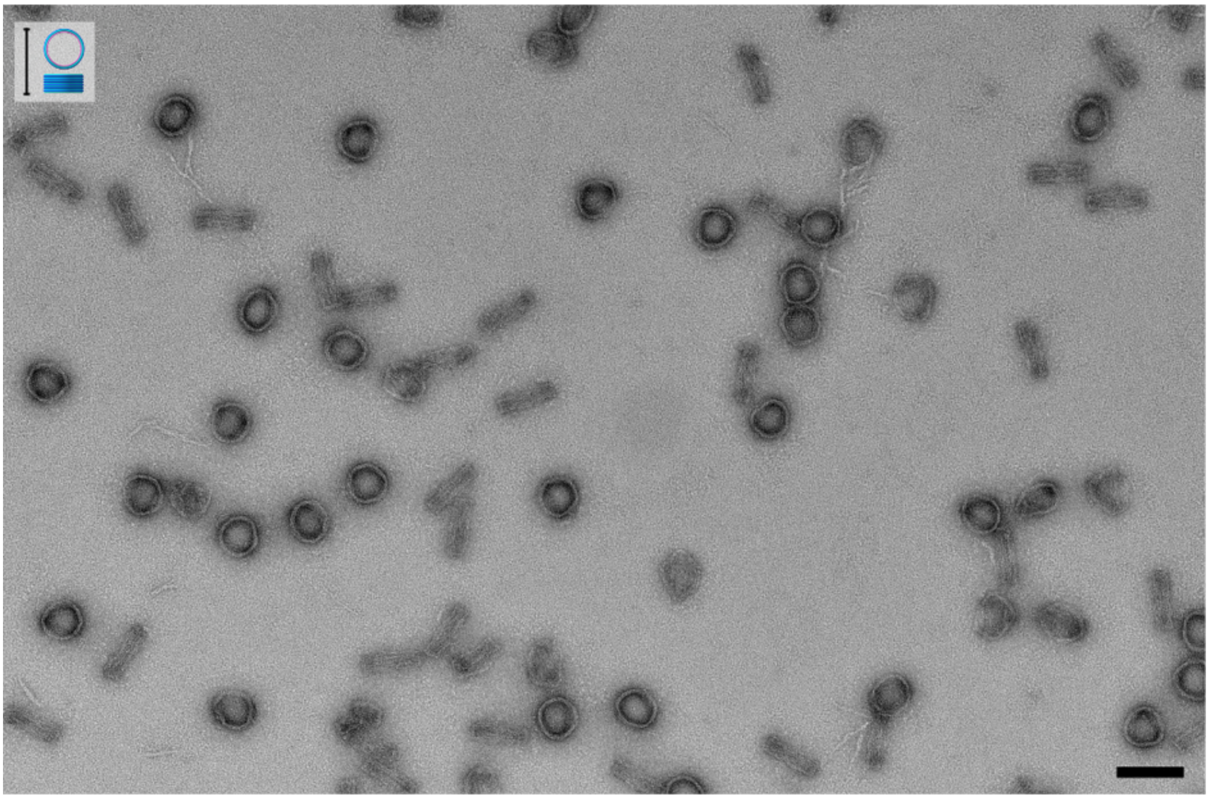

Supplementary Figure 41. Field-of-view TEM 60-27 nm barrel
Scale 100 nm.

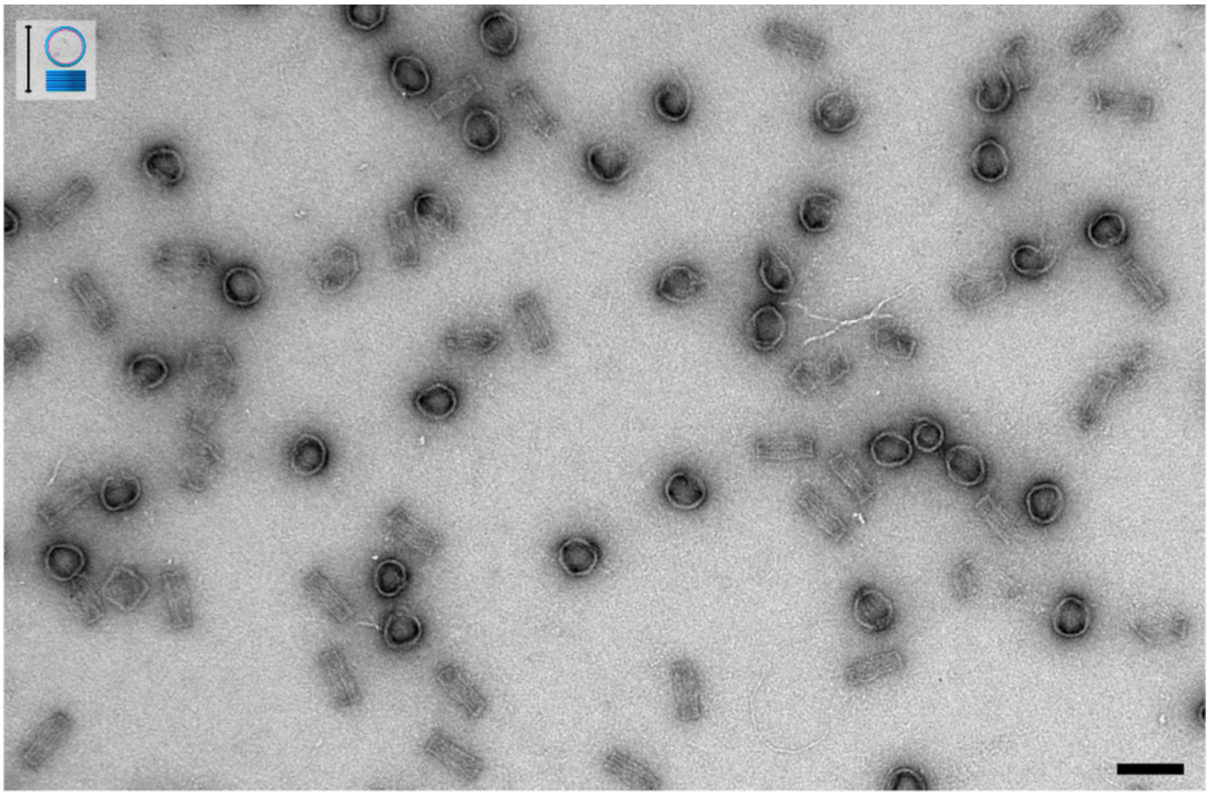

Supplementary Figure 42. Field-of-view TEM 60-32 nm barrel
Scale 100 nm.

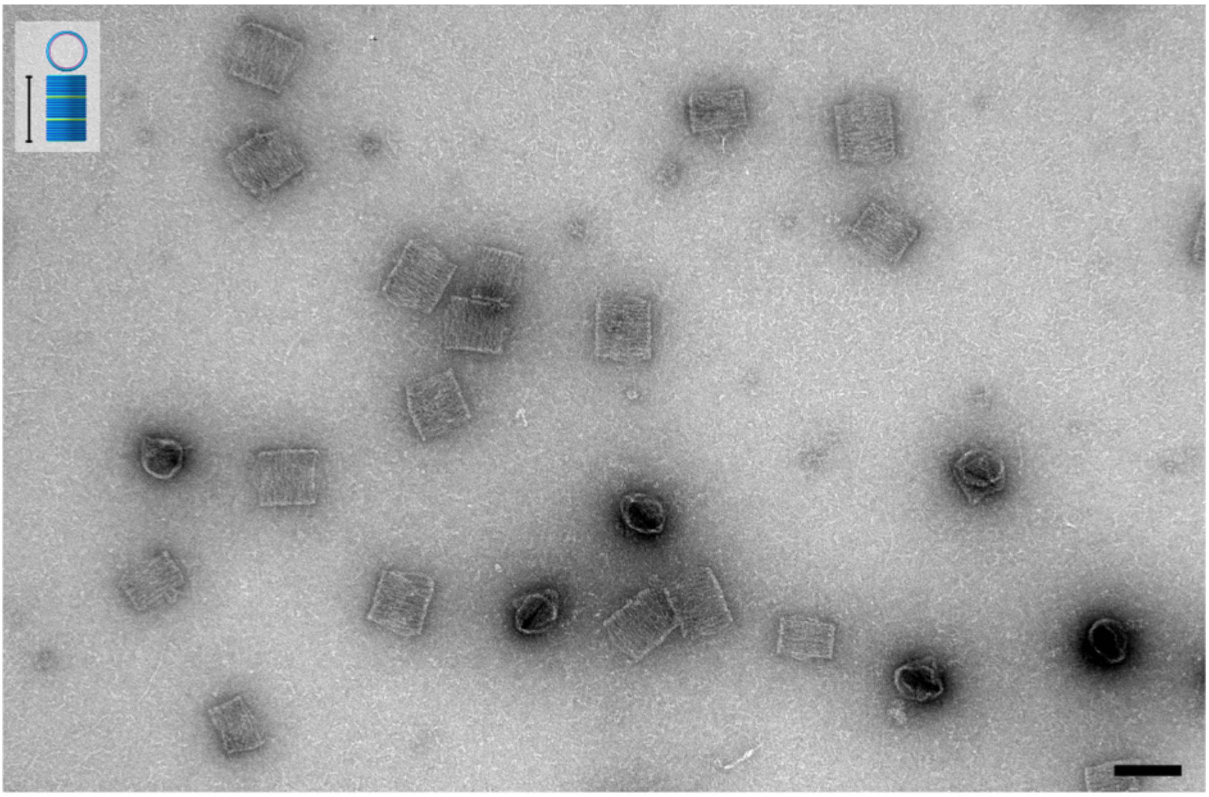

Supplementary Figure 43. Field-of-view TEM 60-27 nm barrel trimer (height = 98 nm)
Scale 100 nm.

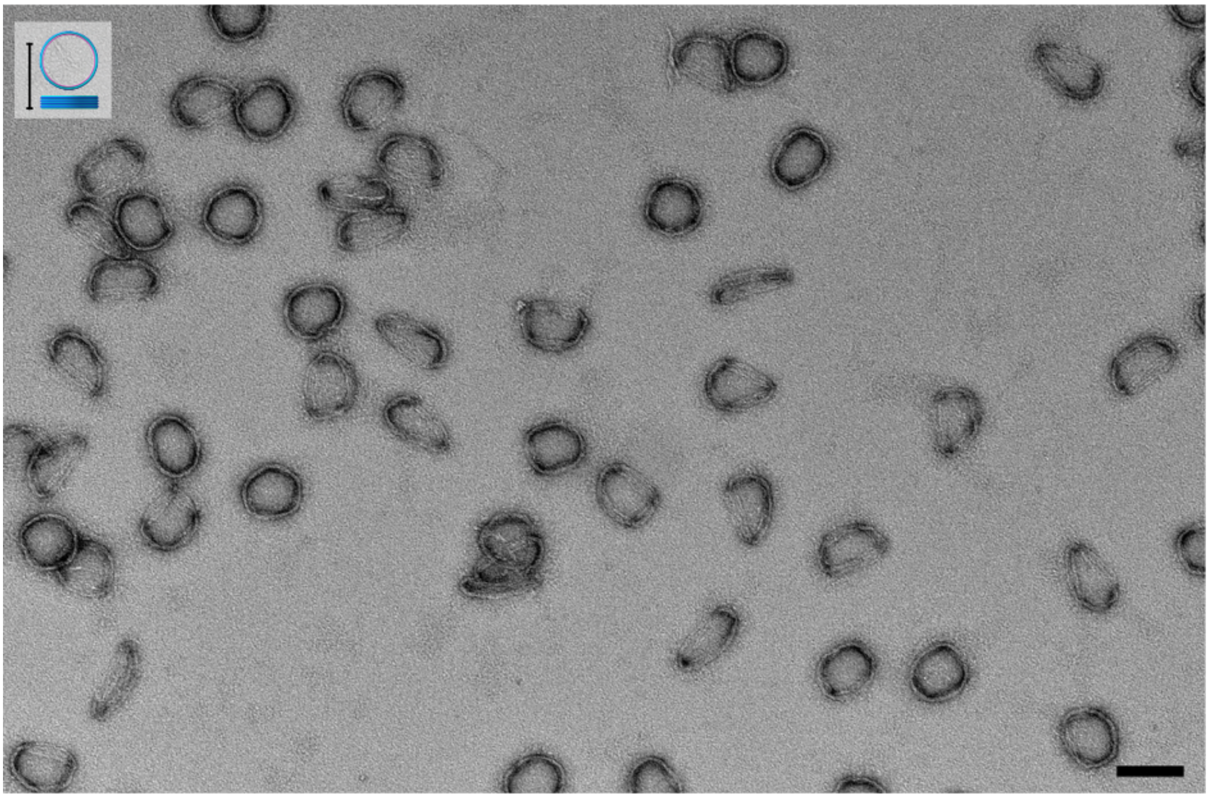

Supplementary Figure 44. Field-of-view TEM 90-19 nm barrel
Scale 100 nm.

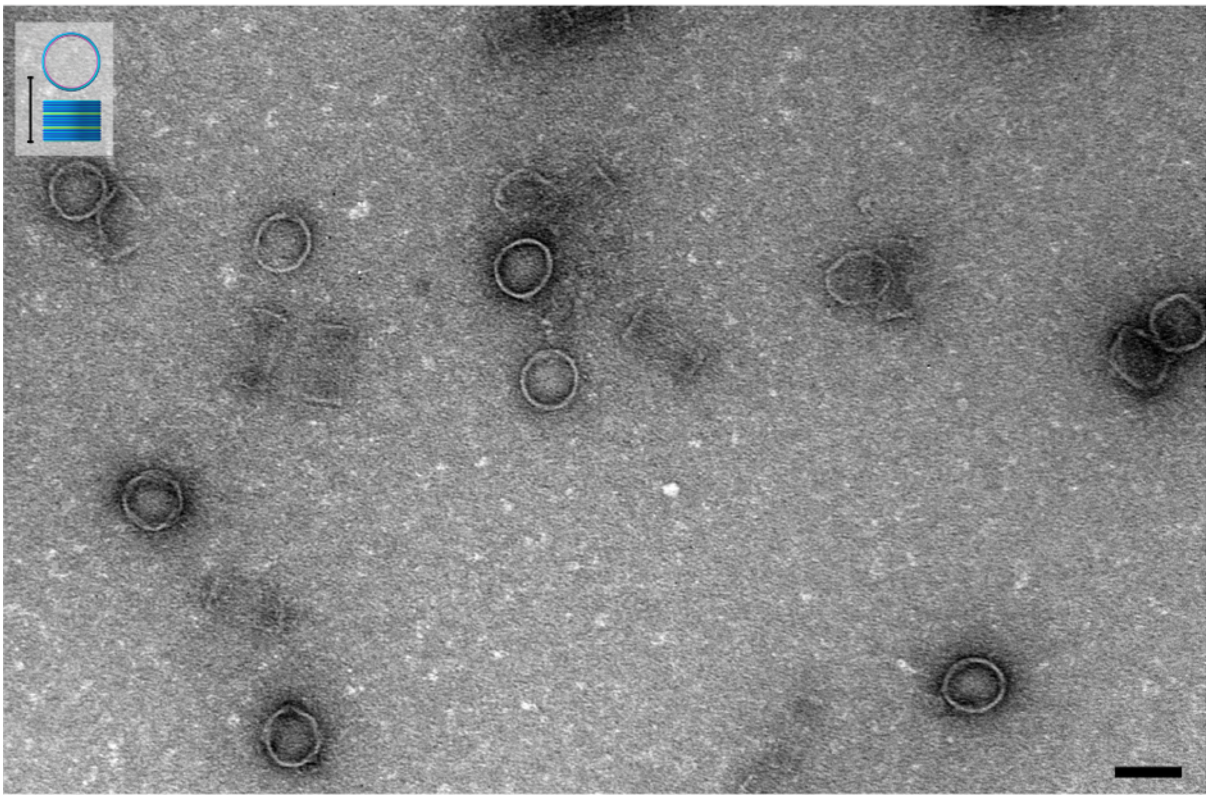

Supplementary Figure 45. Field-of-view TEM 90-19 nm barrel trimer (height = 61 nm), unpurified
Scale 100 nm.

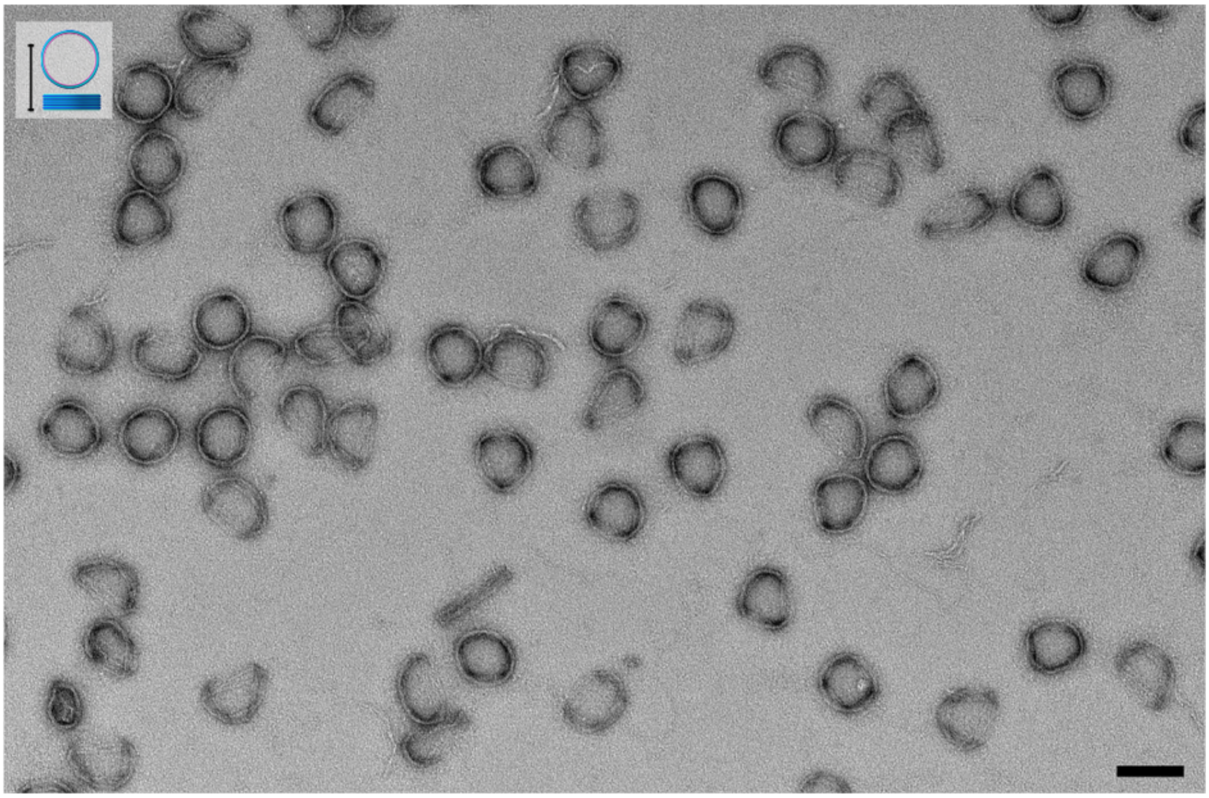

Supplementary Figure 46. Field-of-view TEM 90-23 nm barrel
Scale 100 nm.

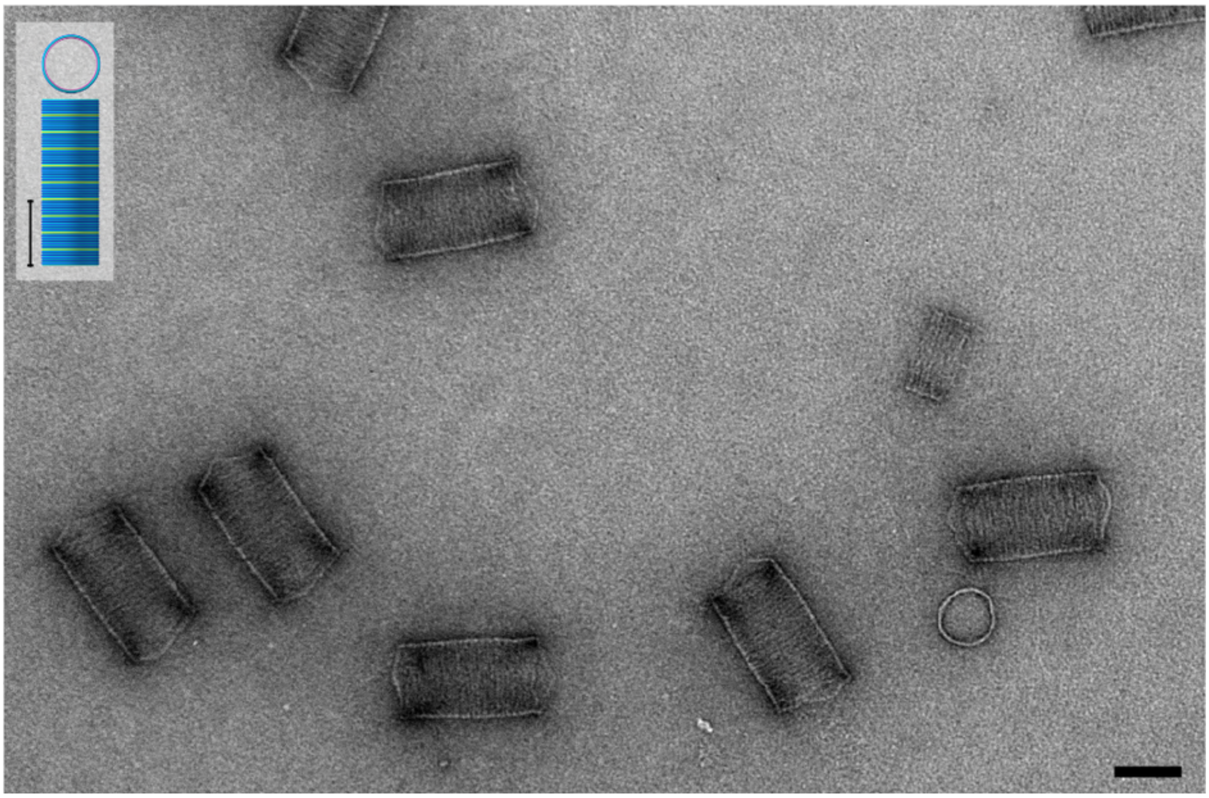

Supplementary Figure 47. Field-of-view TEM 90-23 nm barrel decamer, purified Scale 100 nm.

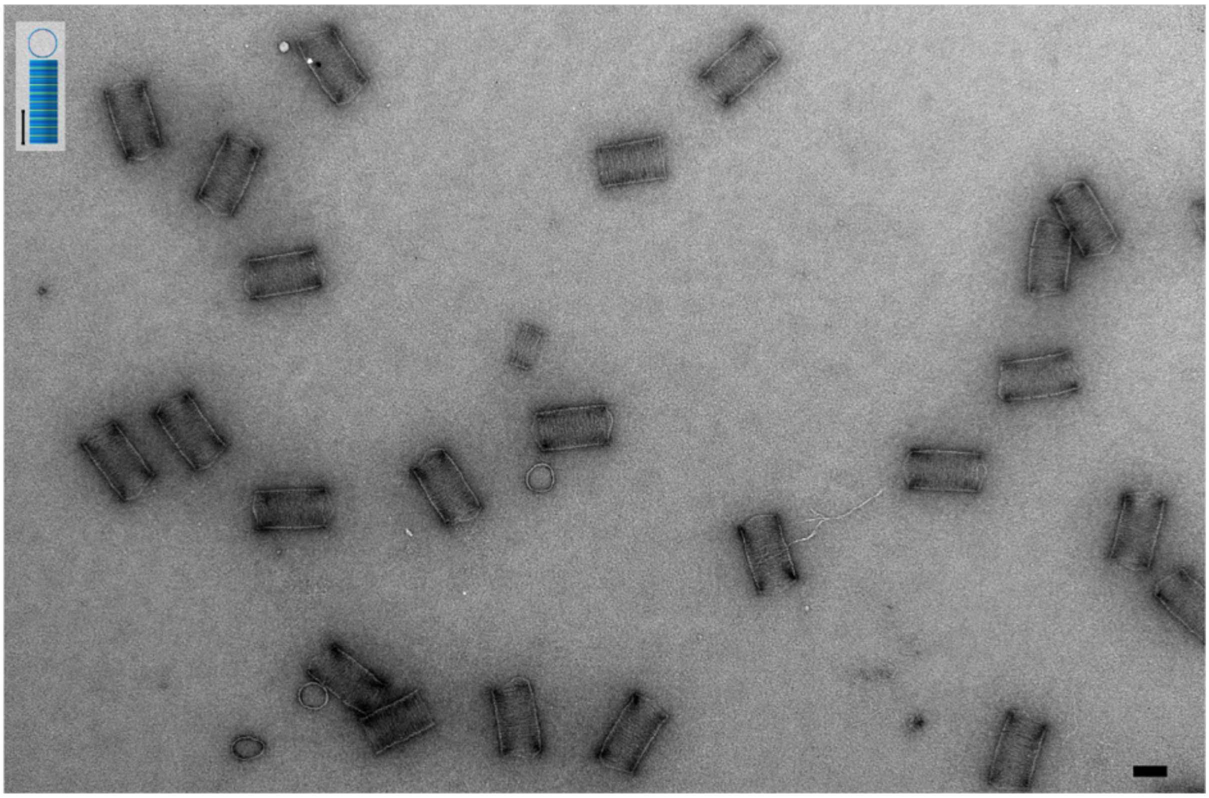

Supplementary Figure 48. Field-of-view TEM 90-23 nm barrel decamer, purified Scale 100 nm.

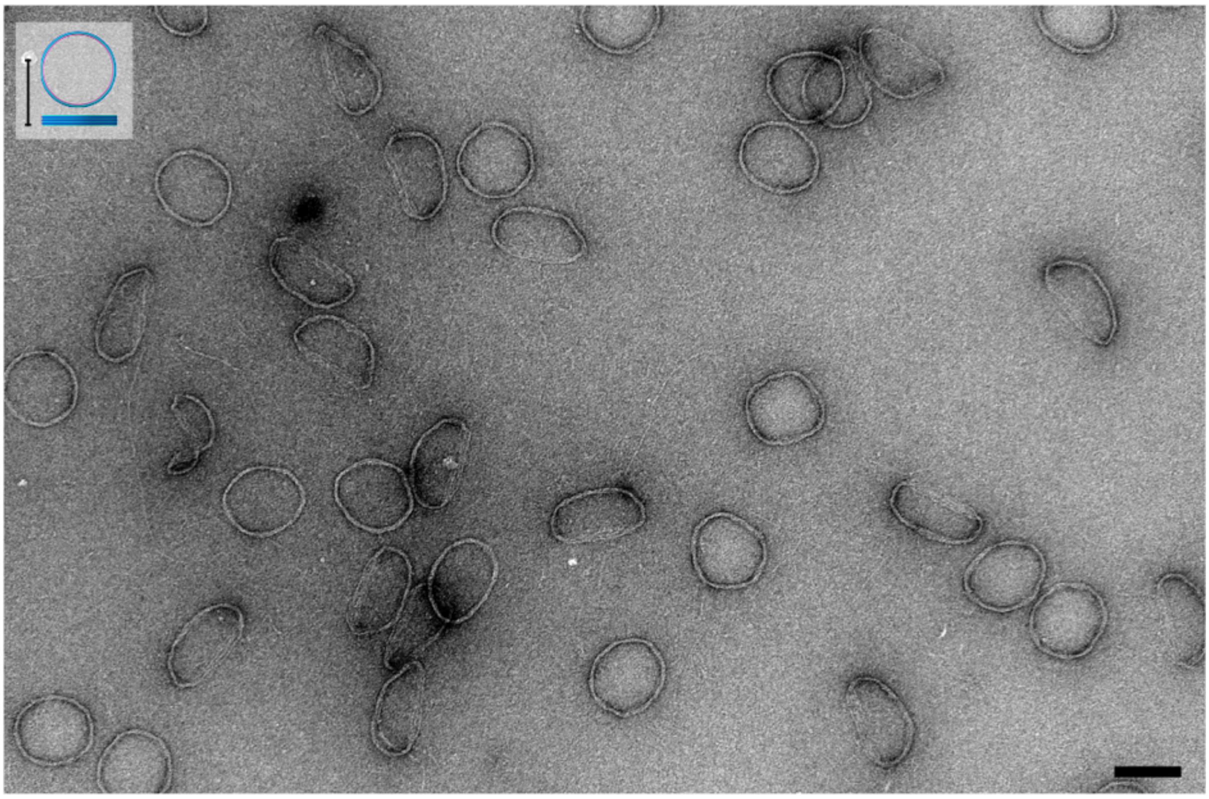

Supplementary Figure 49. Field-of-view TEM 120-15 nm barrel

Scale 100 nm.

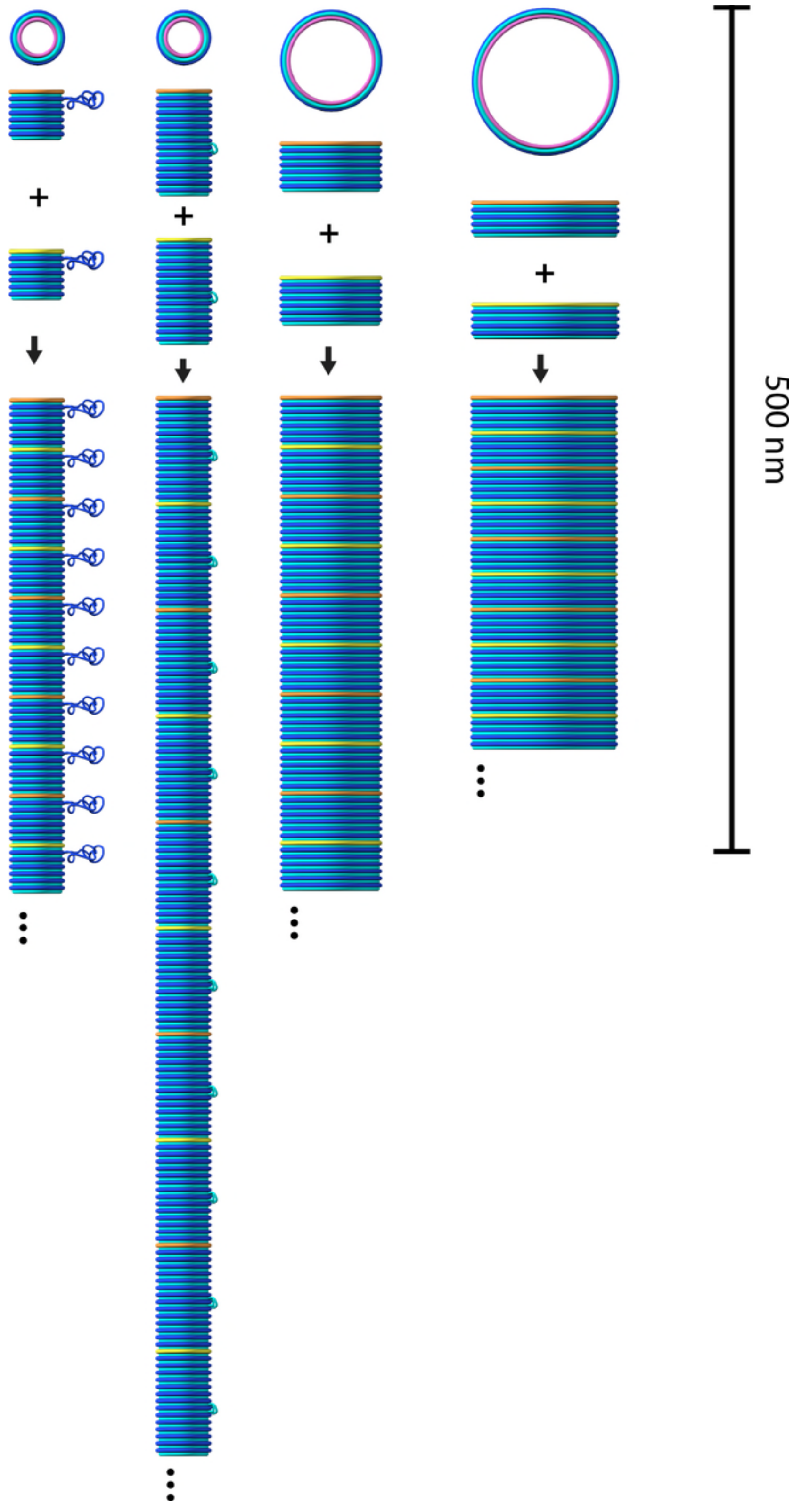

Supplementary Figure 50. 3D models of DNA barrel monomers and polymers

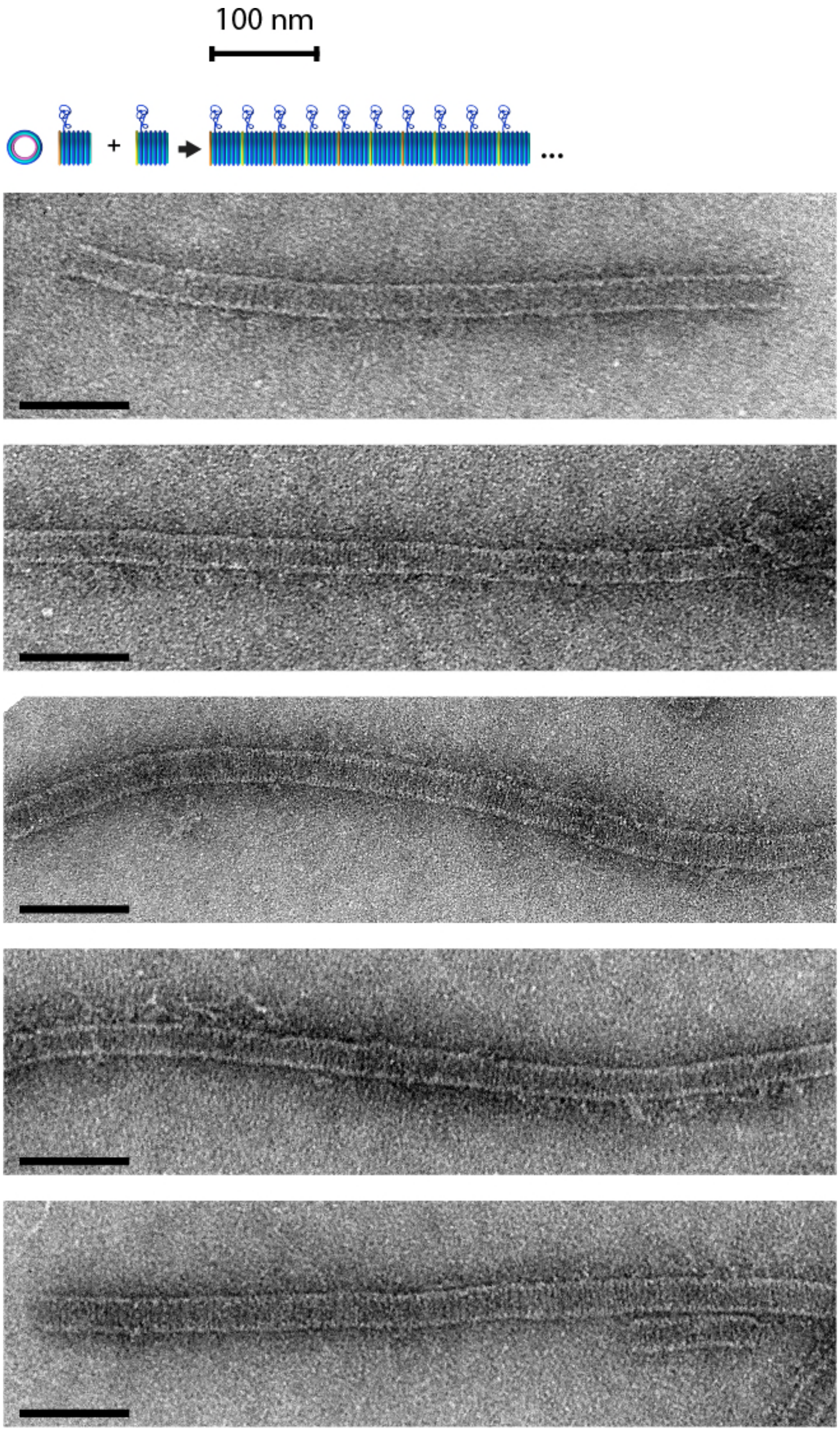

Supplementary Figure 51. Cropped TEMs, 30-27 nm polymer
Scale 100 nm.

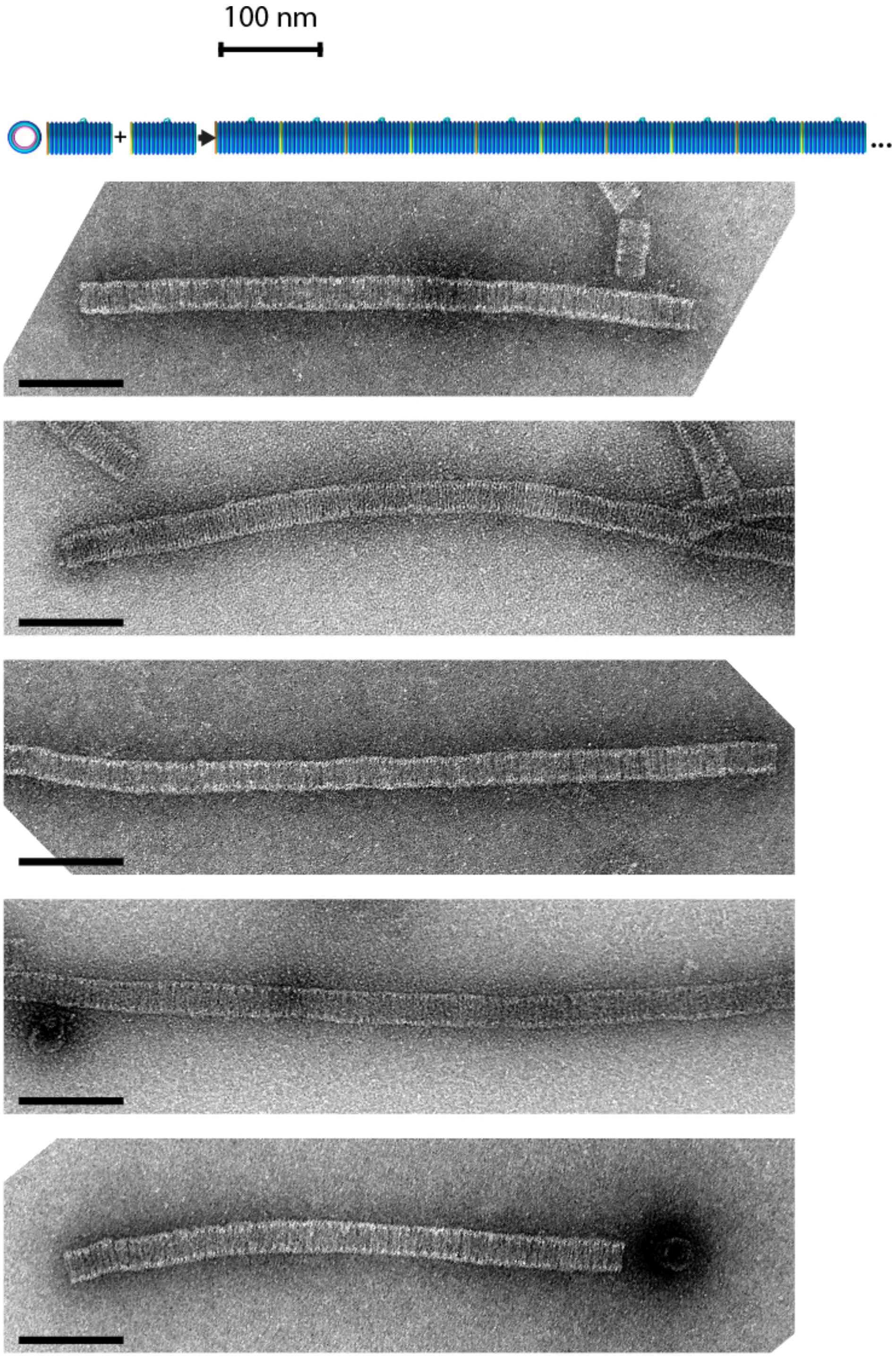

Supplementary Figure 52. Cropped TEMs, 30-65 nm polymer
Scale 100 nm.

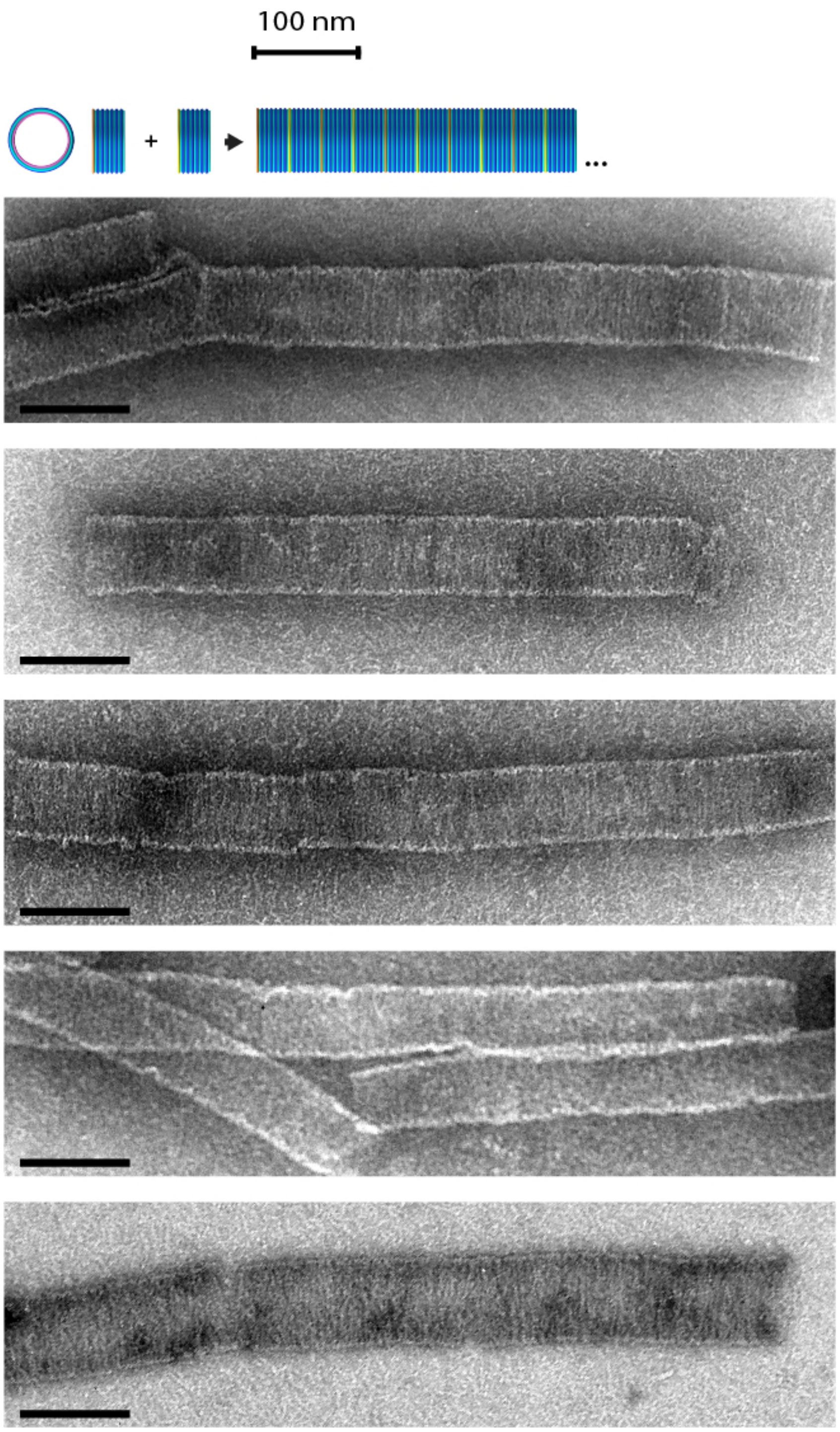

Supplementary Figure 53. Cropped TEMs, 60-27 nm polymer
Scale 100 nm.

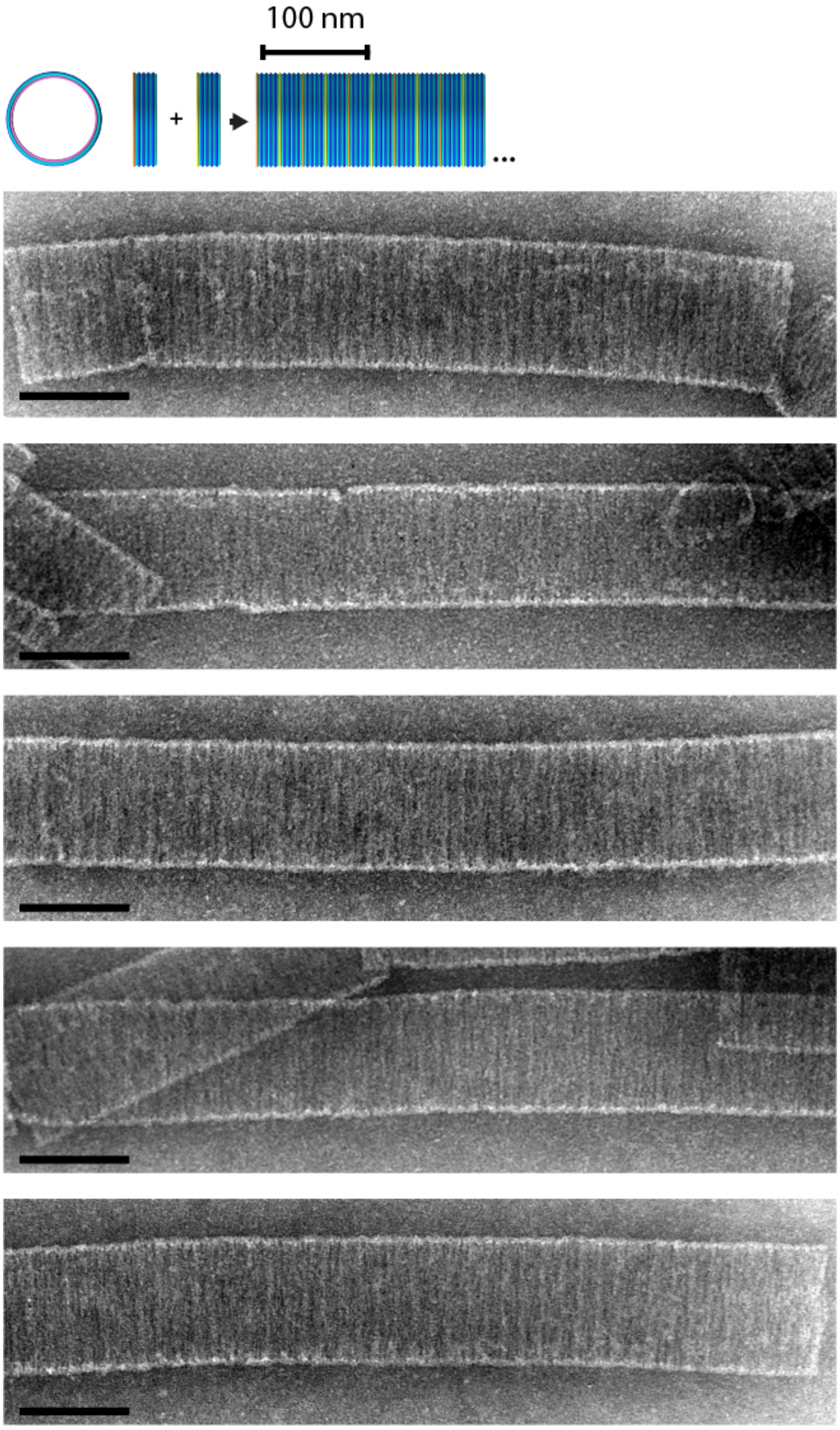

Supplementary Figure 54. Cropped TEMs, 90-19 nm polymer

Scale 100 nm.

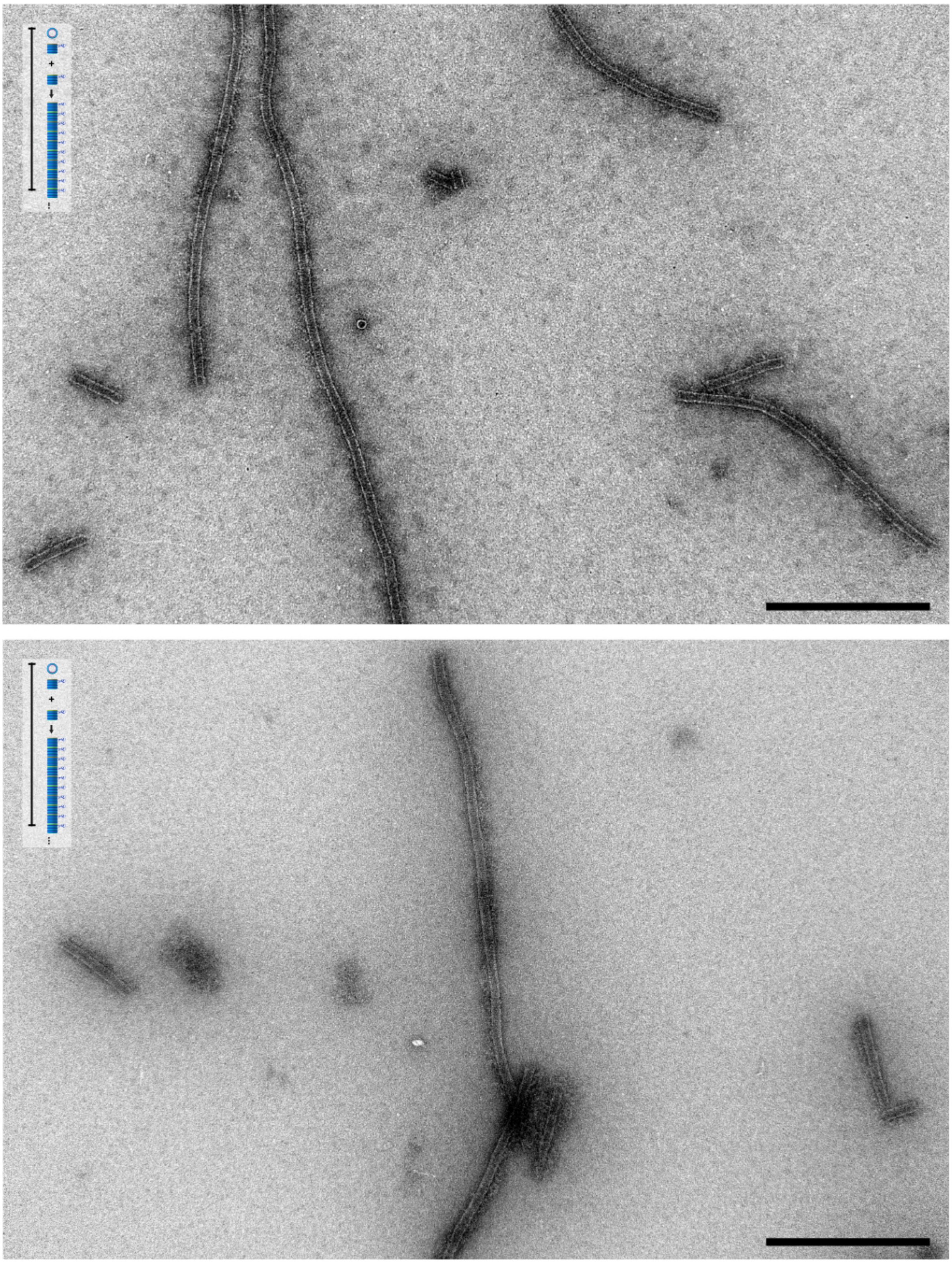

Supplementary Figure 55. Field-of-view TEM 30-27 nm barrel polymer

Scale 500 nm.

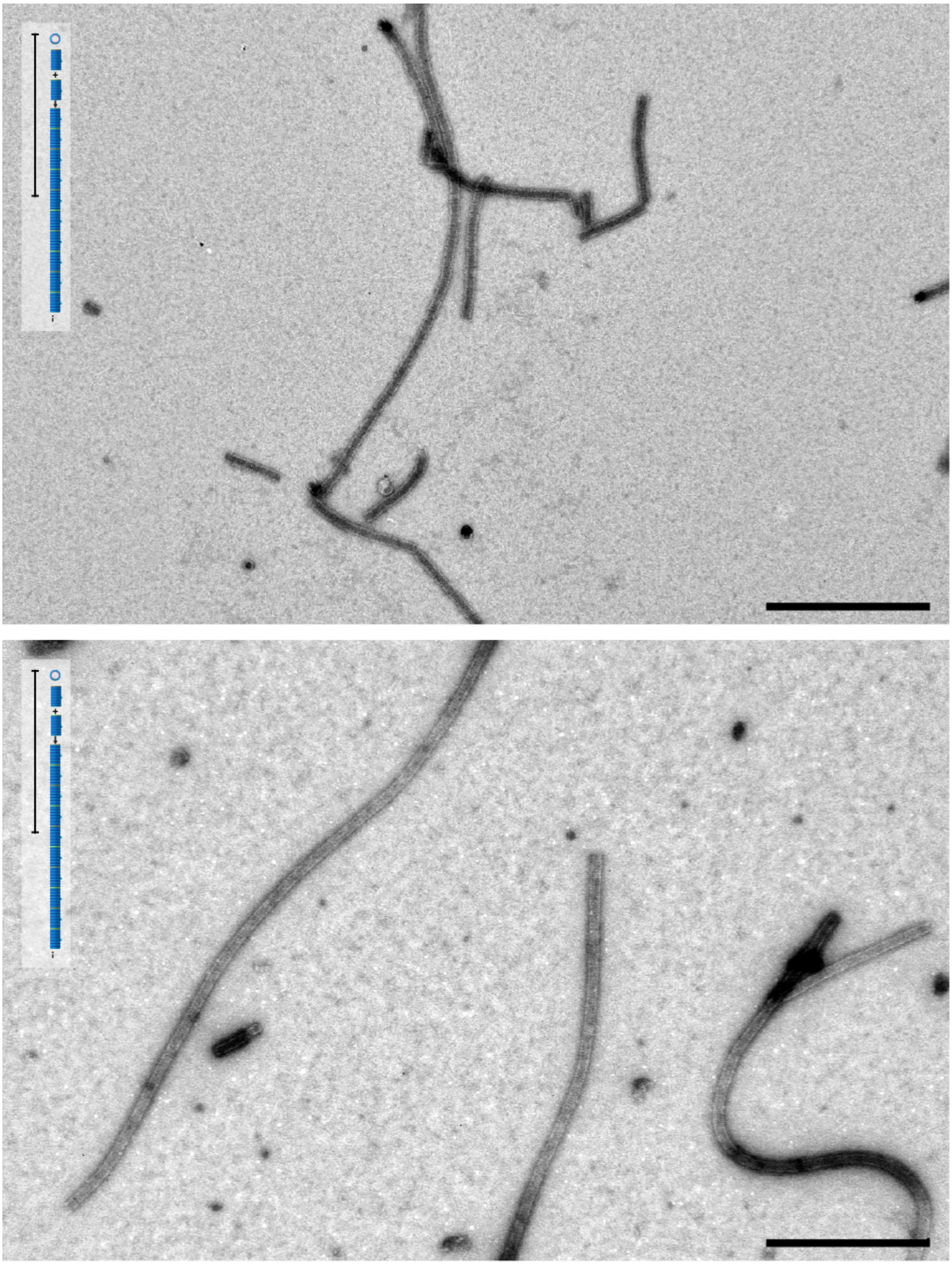

Supplementary Figure 56. Field-of-view TEM 30-65 nm barrel polymer

Scale 500 nm.

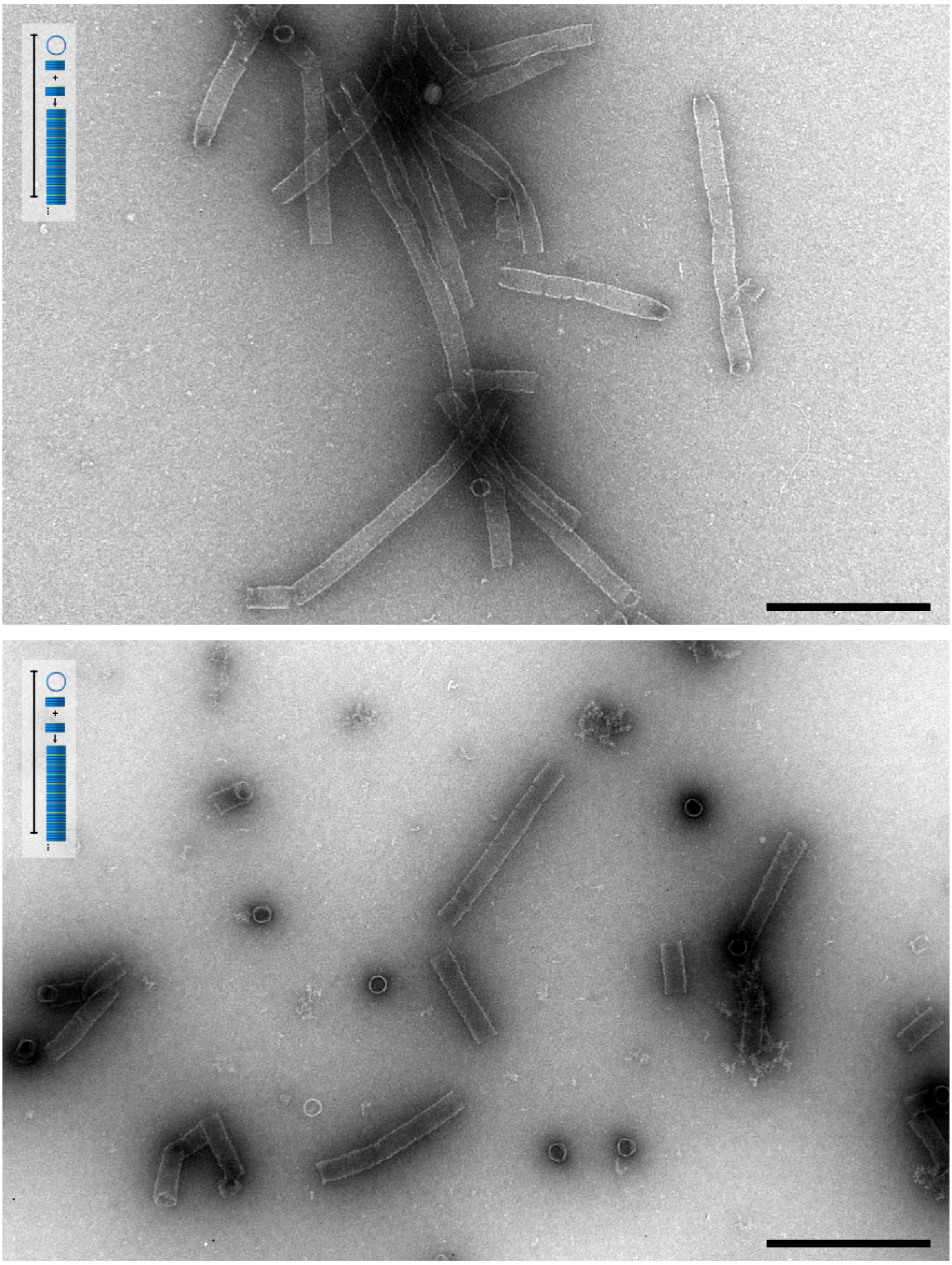

Supplementary Figure 57. Field-of-view TEM 60-27 nm barrel polymer

Scale 500 nm.

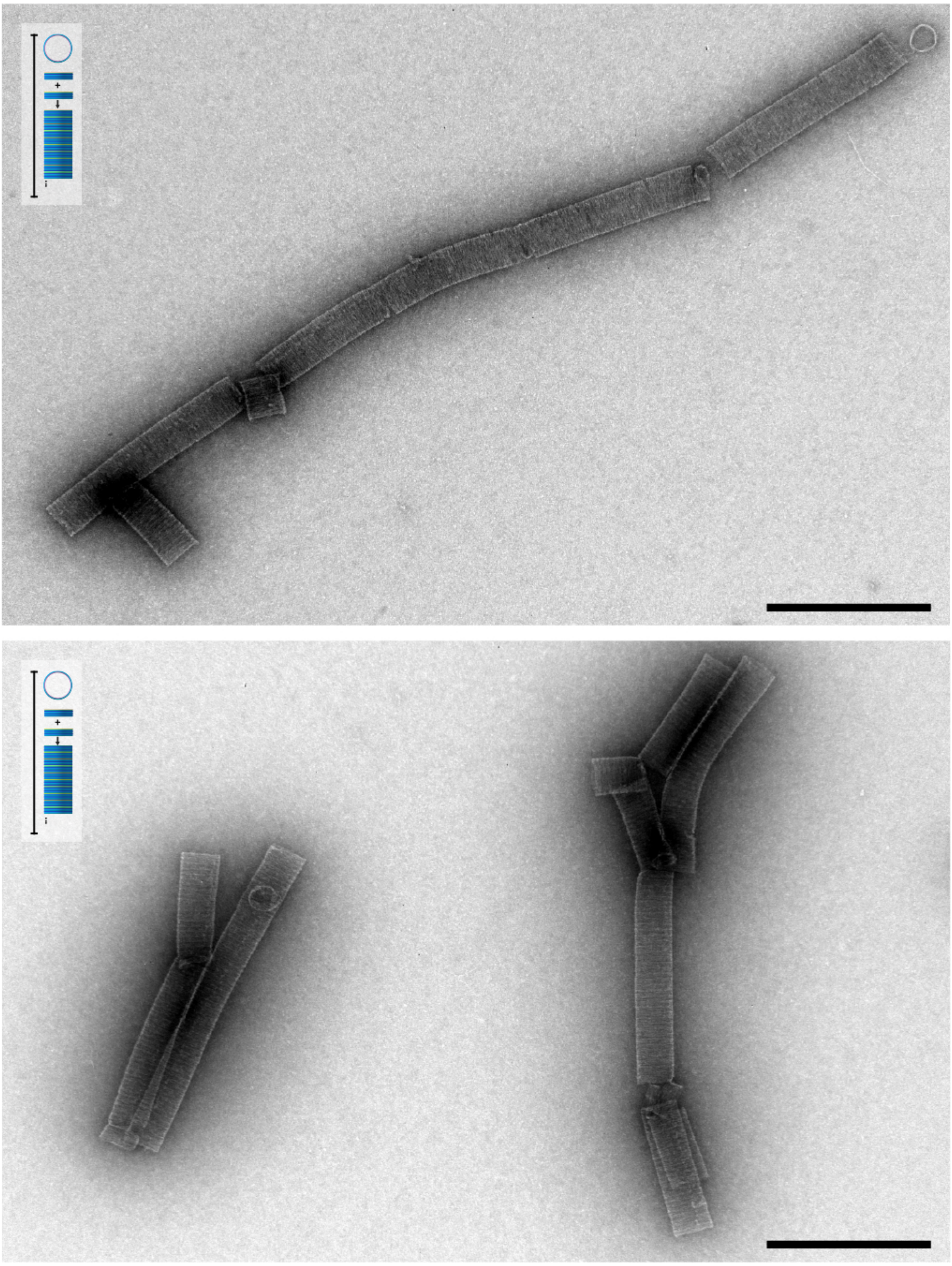

Supplementary Figure 58. Field-of-view TEM 90-19 nm barrel polymer

Scale 500 nm.

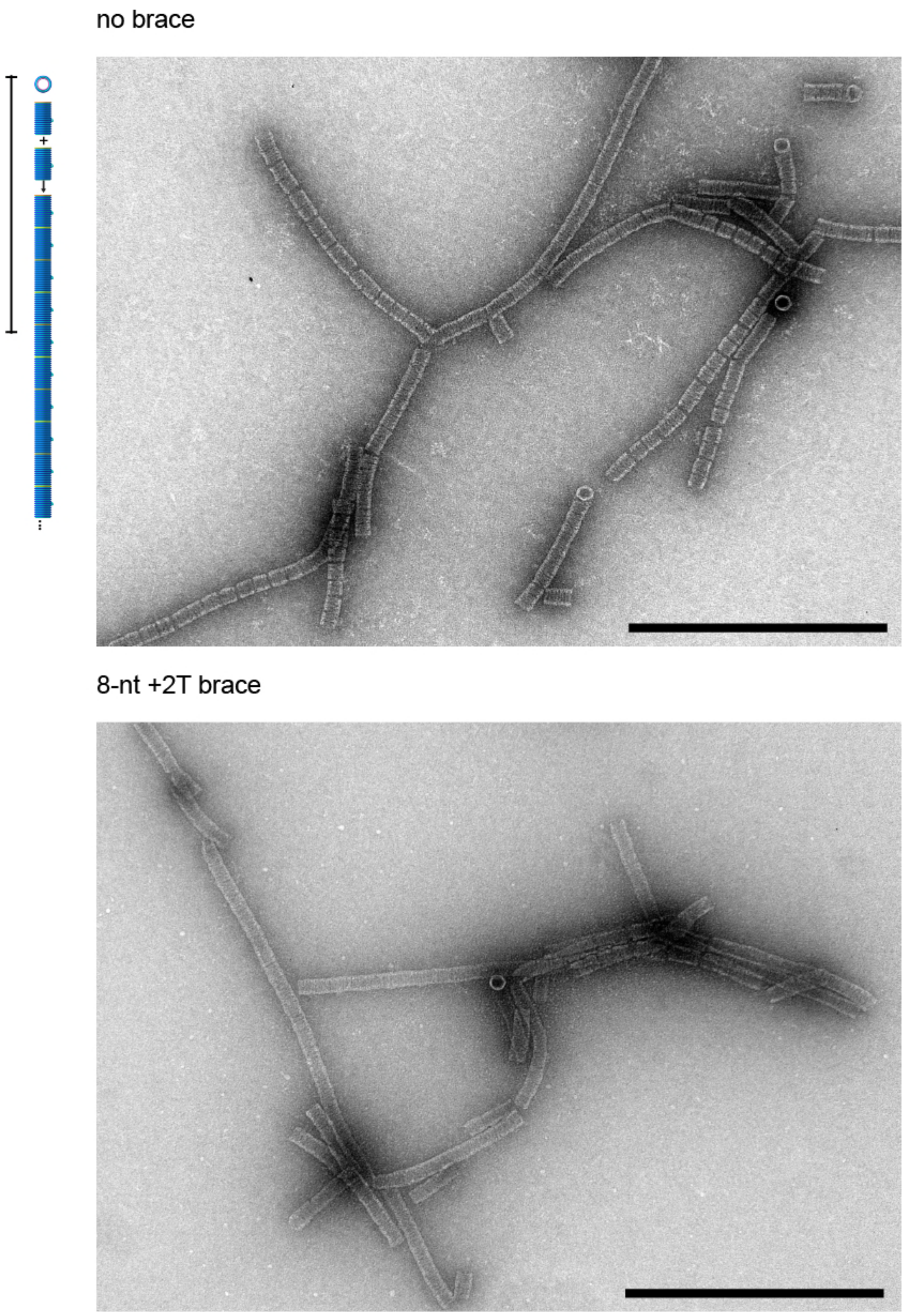

Supplementary Figure 59. 30-65 nm barrel polymer, with and without coaxial brace strands
Scale 500 nm.

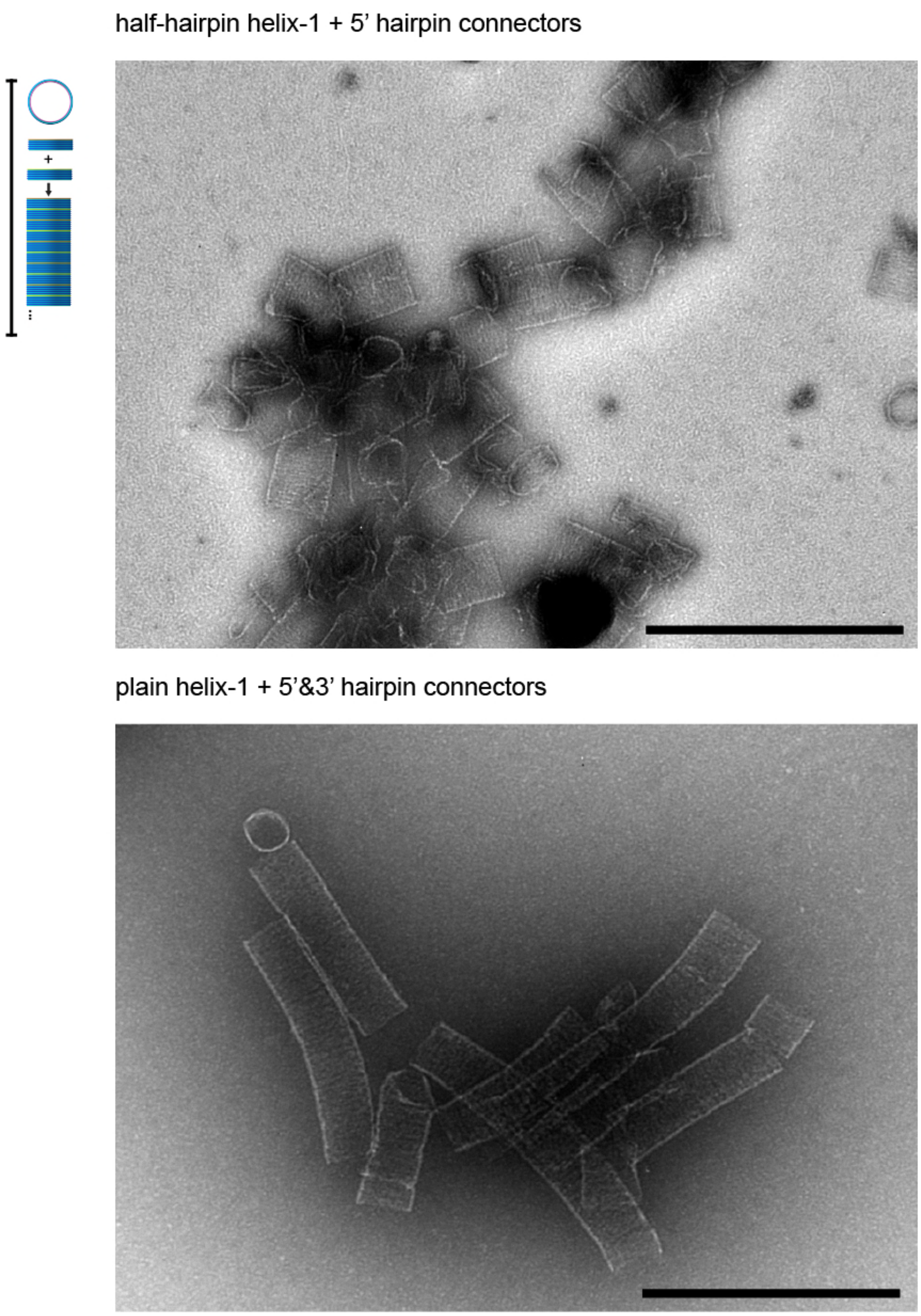

Supplementary Figure 60. 90-19 nm barrel polymer, before and after interface design optimisation Scale 500 nm.

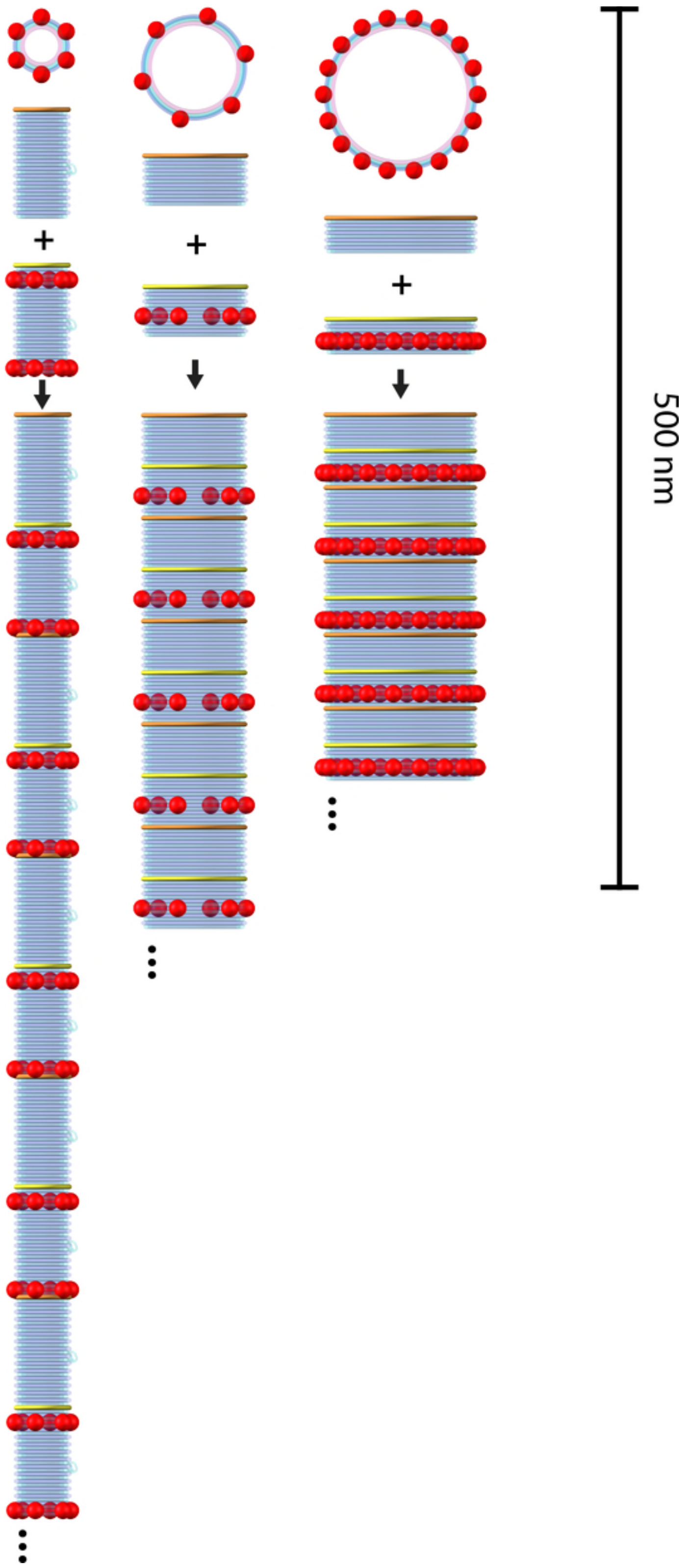

Supplementary Figure 61. 3D models of DNA-PAINT barrel monomers and polymers.

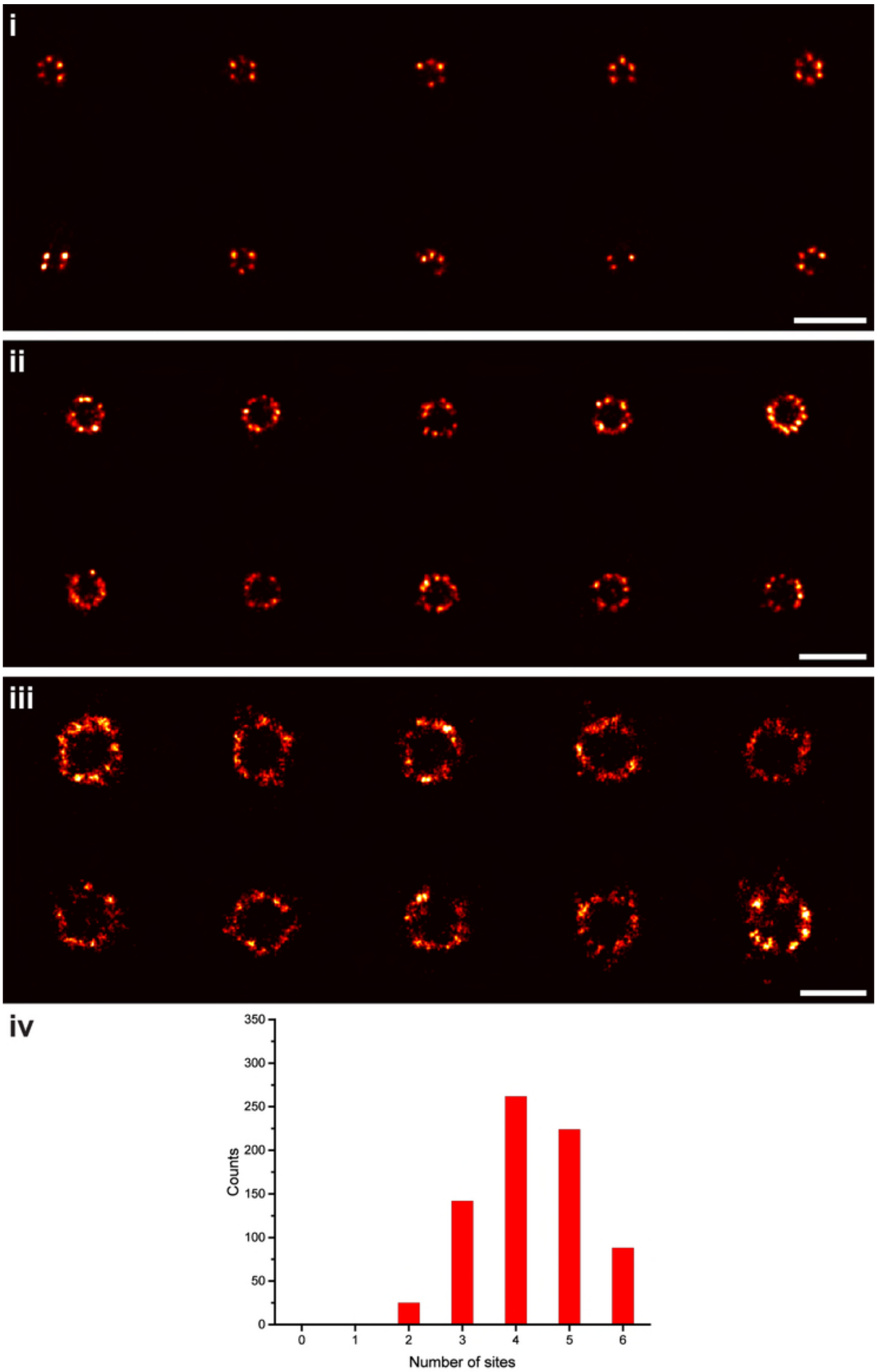

Supplementary Figure 62 Cropped DNA-PAINT monomers

(i) 30-65 nm, (ii) 60-27 nm, (iii) 90-19 nm. (iv) Histogram of number of observed DNA-PAINT localisation clusters for 30-65 nm data set (N = 741). Average number of sites is 4.3 out of a total 6, and average site occupancy is 71%. Scale 100 nm.

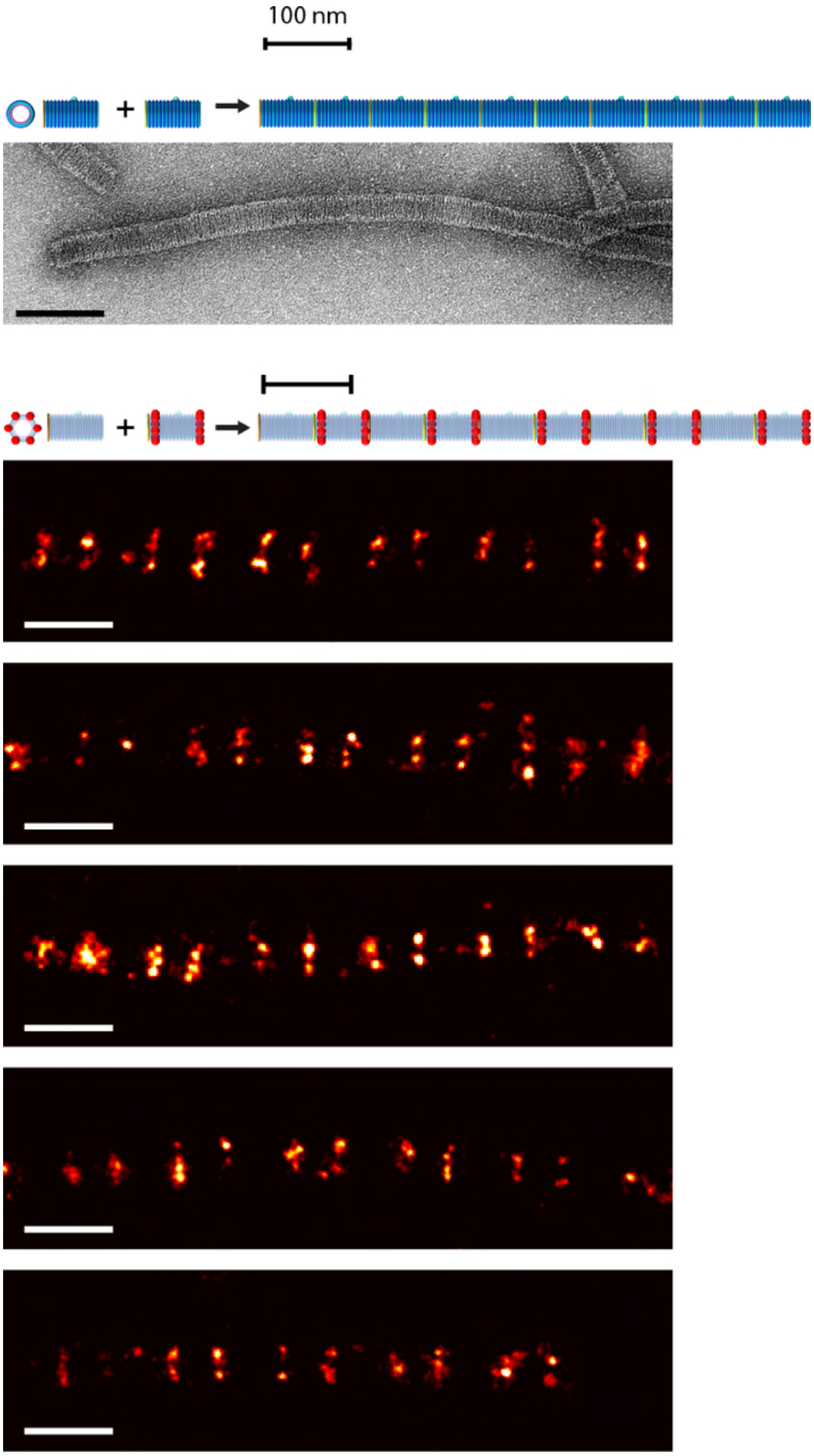

Supplementary Figure 63 Cropped DNA-PAINT polymers, 30-65 nm, scale 100 nm

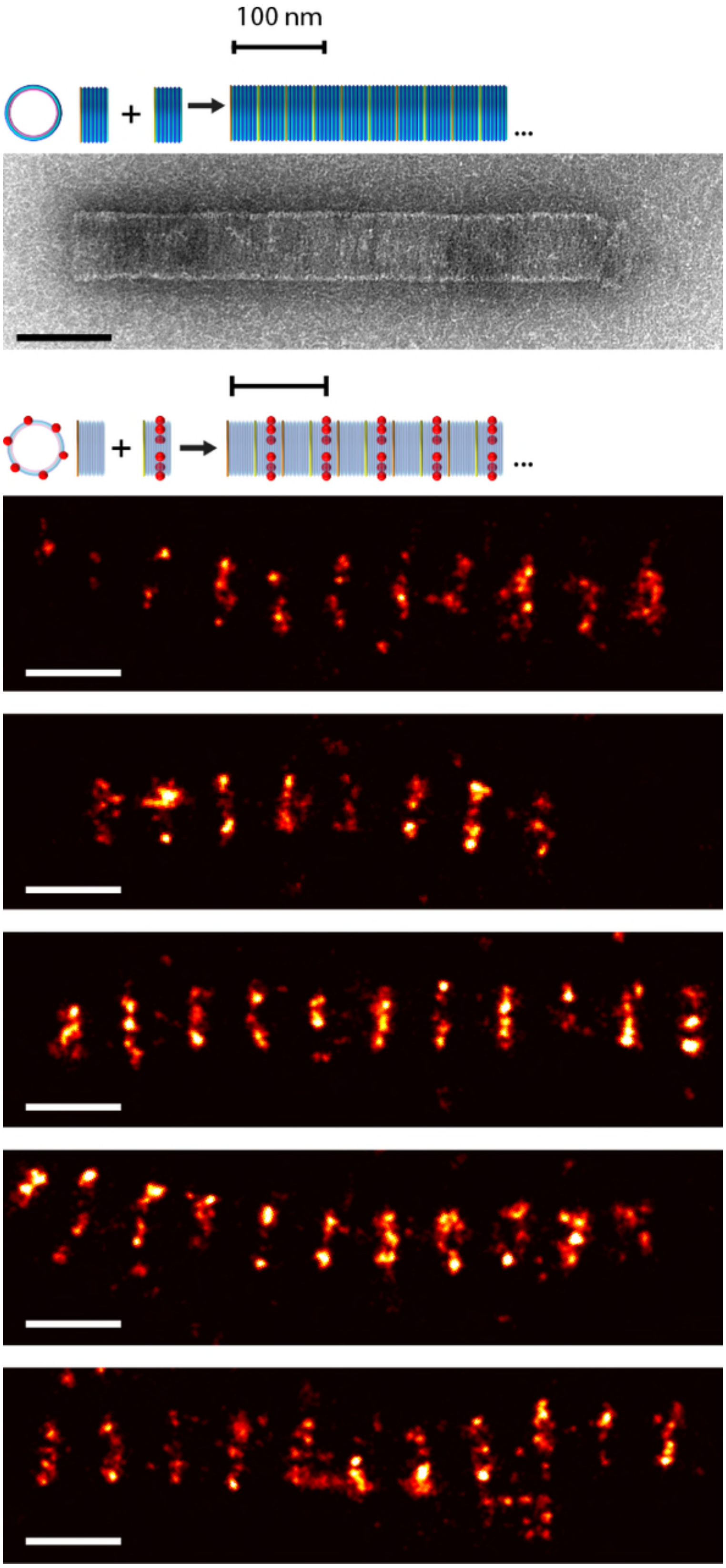

Supplementary Figure 64 Cropped DNA-PAINT polymers, 60-27 nm, scale 100 nm

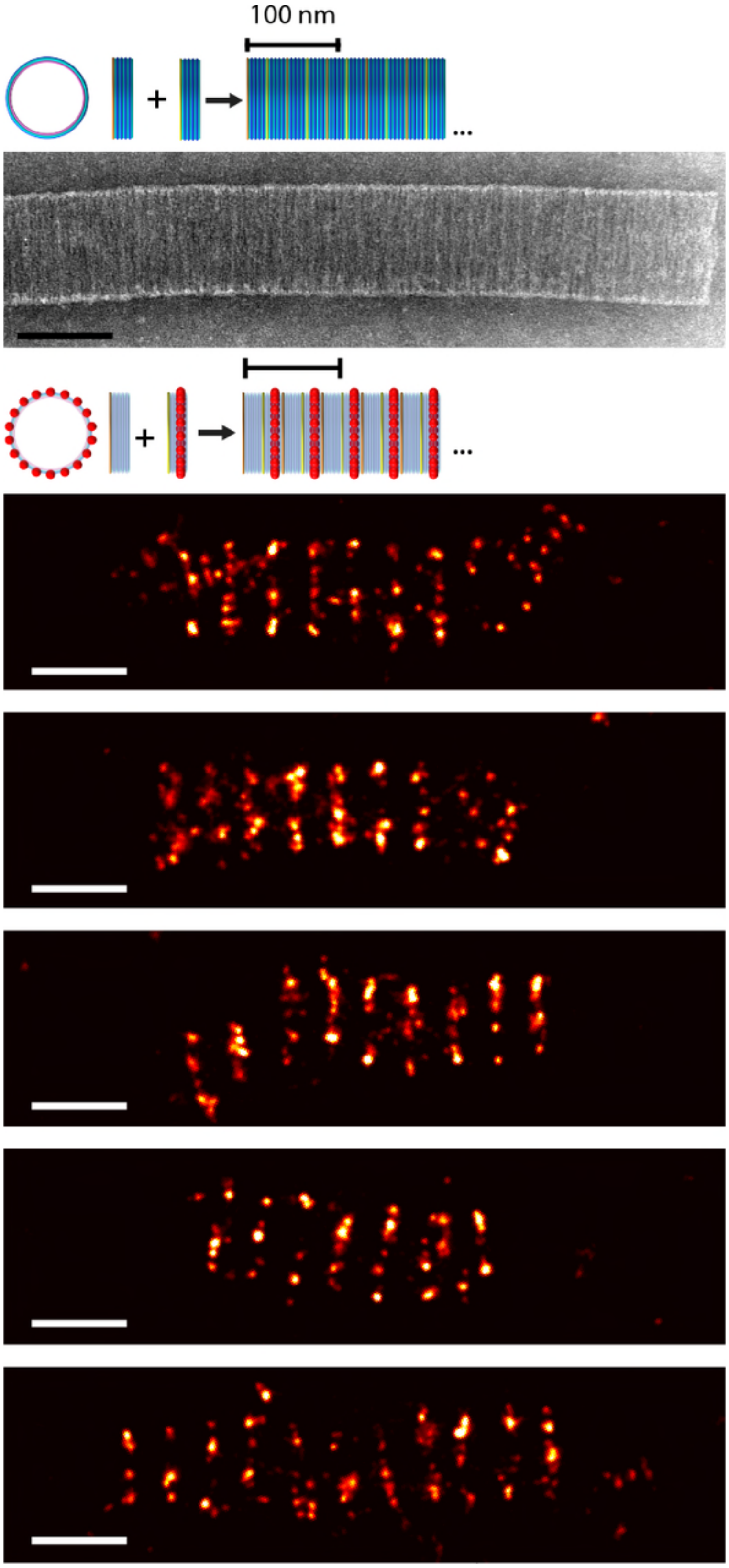

Supplementary Figure 65 Cropped DNA-PAINT polymers, 90-19 nm, scale 100 nm

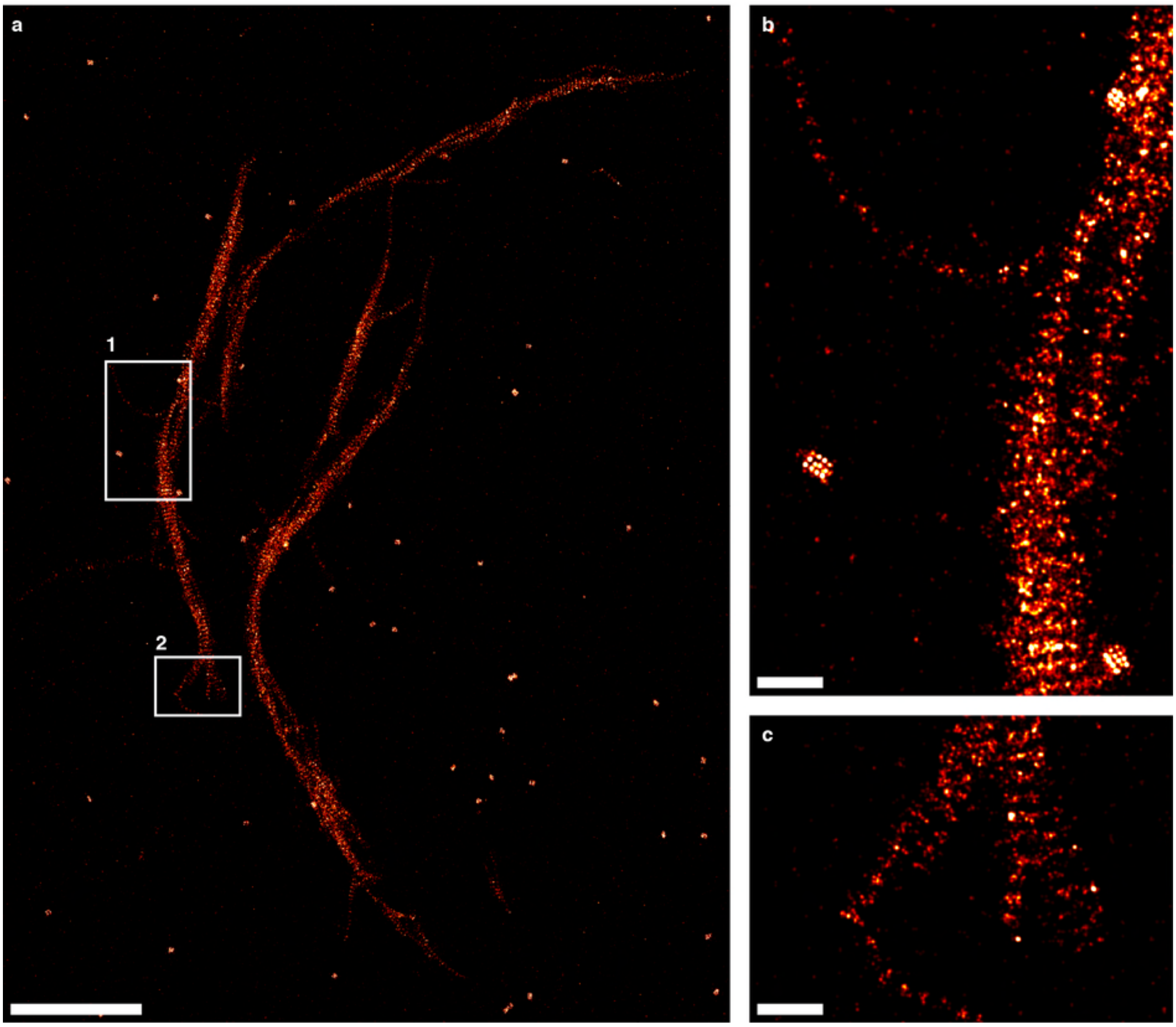

Supplementary Figure 66. Field-of-view DNA-PAINT polymers, 90-19 nm, extended polymers

Scale bars 2 um (a) and 200 nm (b,c)

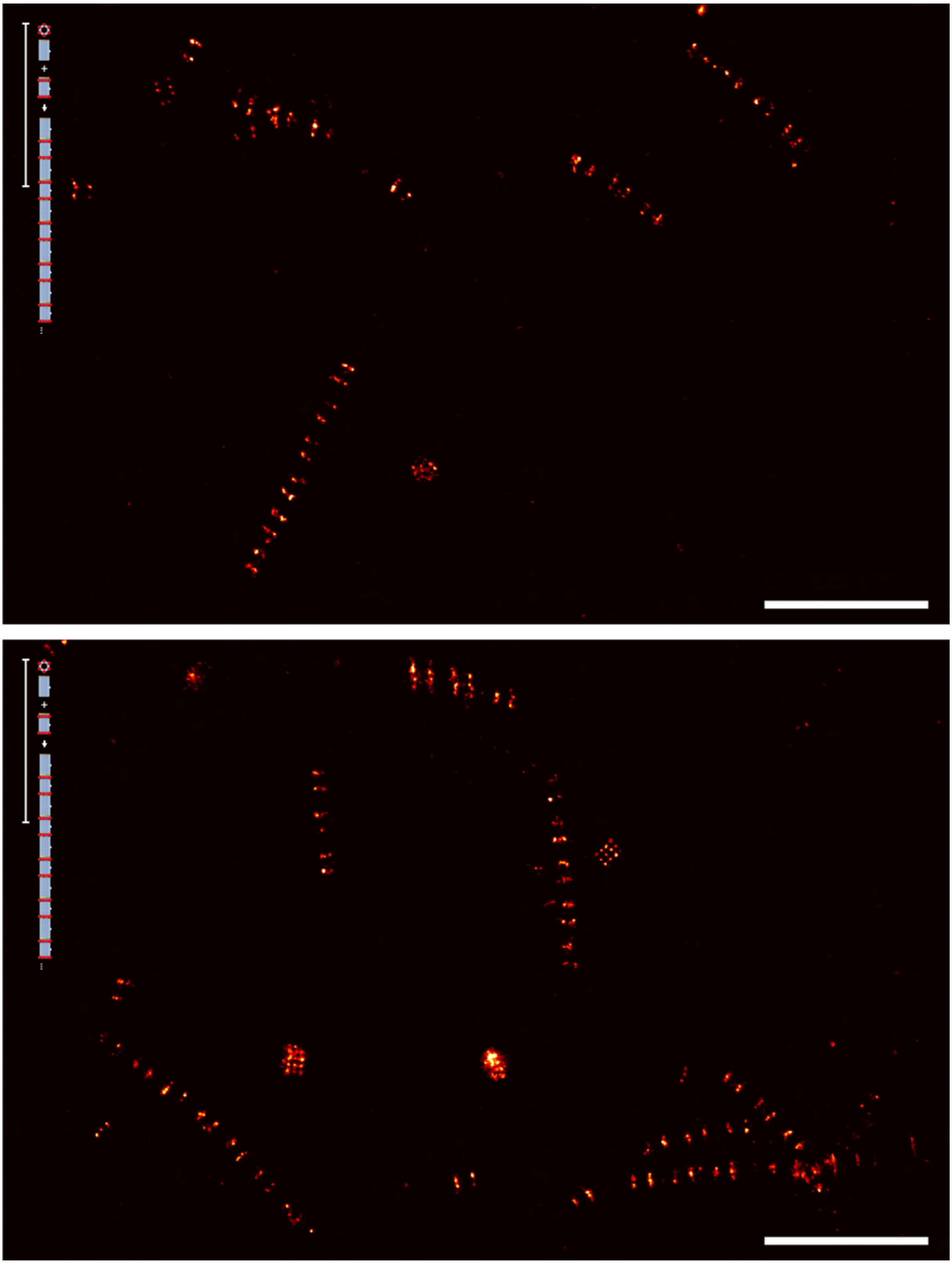

Supplementary Figure 67. Field-of-view DNA-PAINT polymers, 30-65 nm, truncated

Scale 500 nm

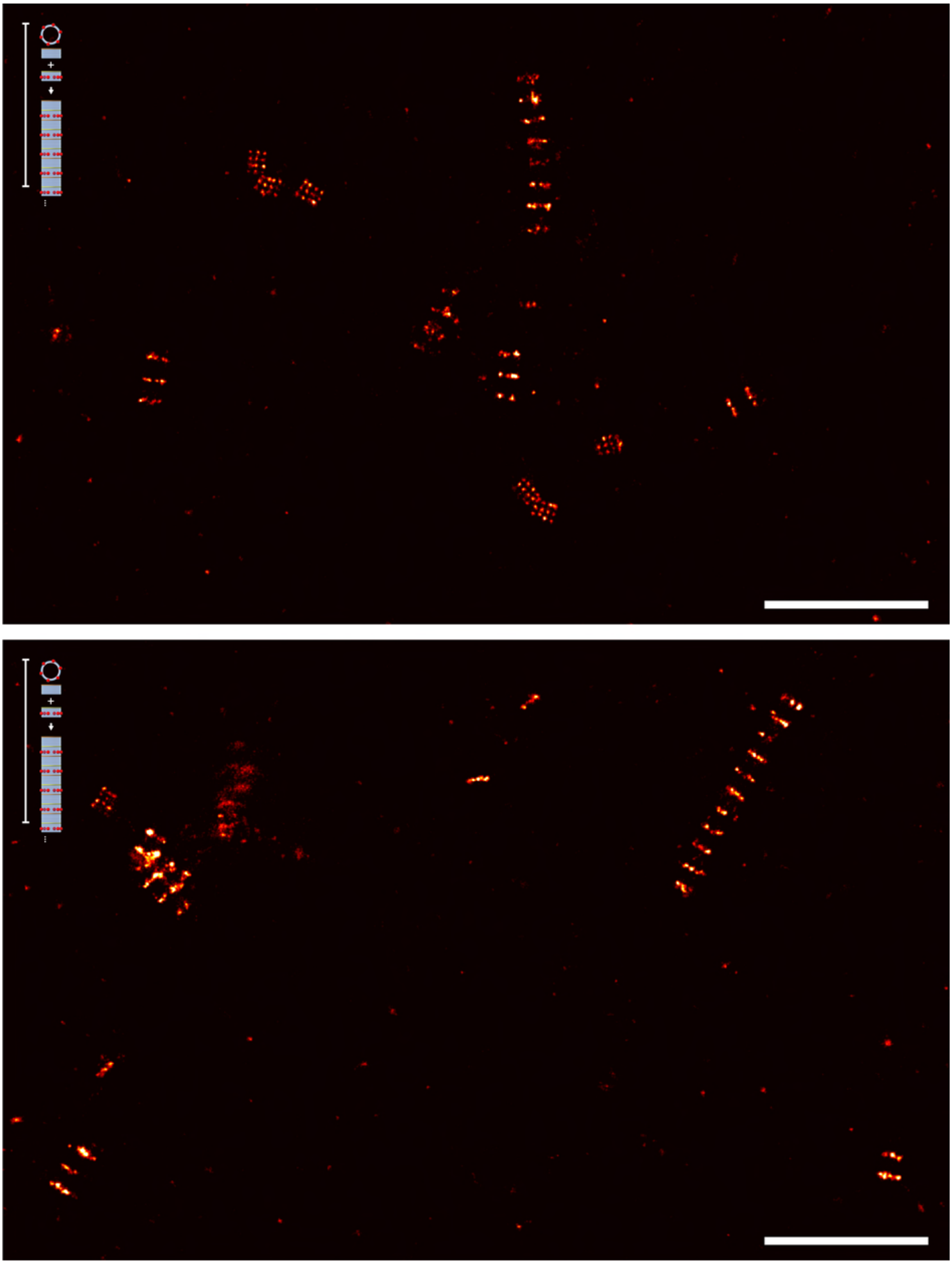

Supplementary Figure 68. Field-of-view DNA-PAINT polymers, 60-27 nm, truncated
Scale 500 nm.

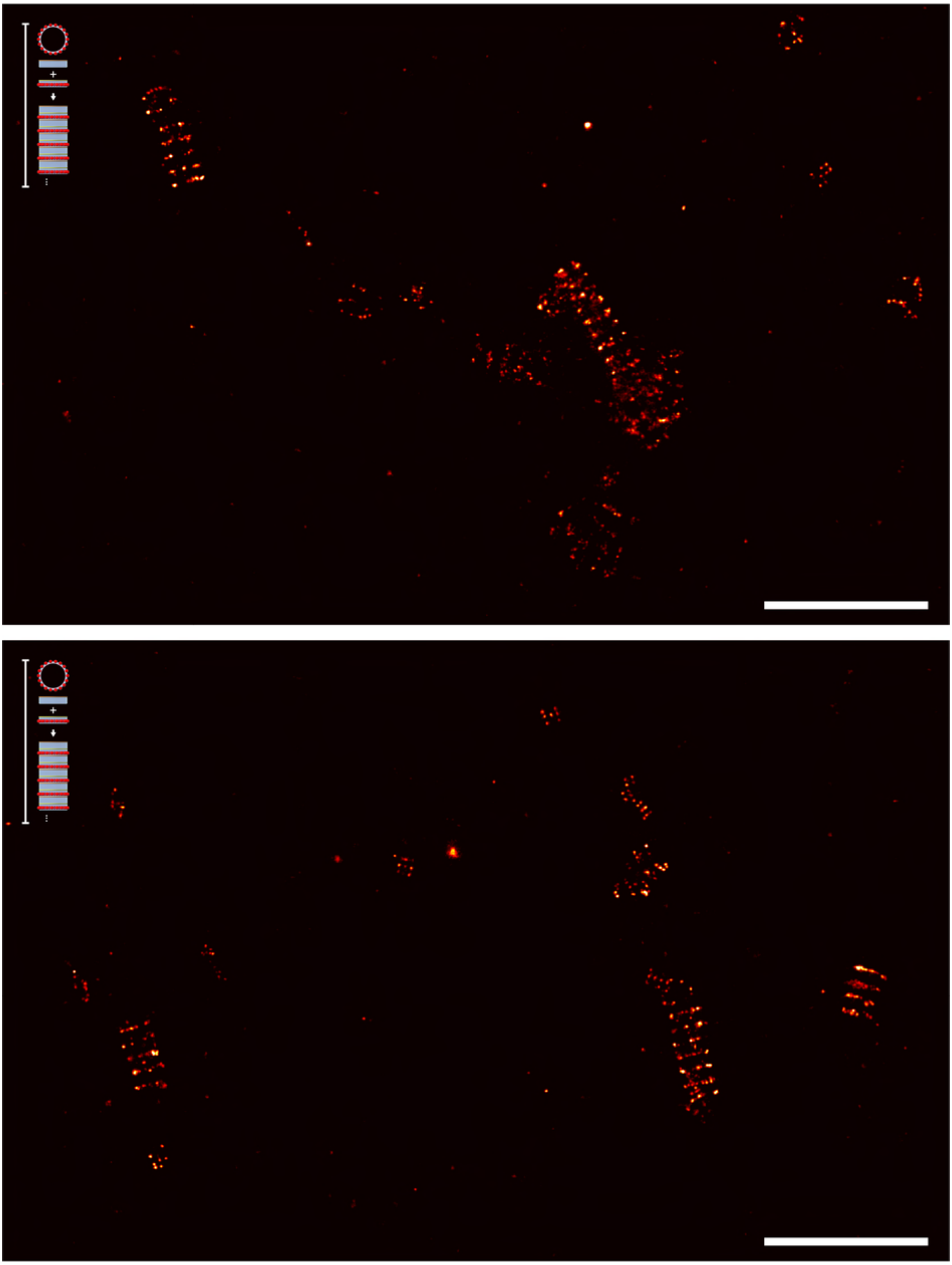

Supplementary Figure 69. Field-of-view DNA-PAINT polymers, 90-19 nm, truncated
Scale 500 nm.

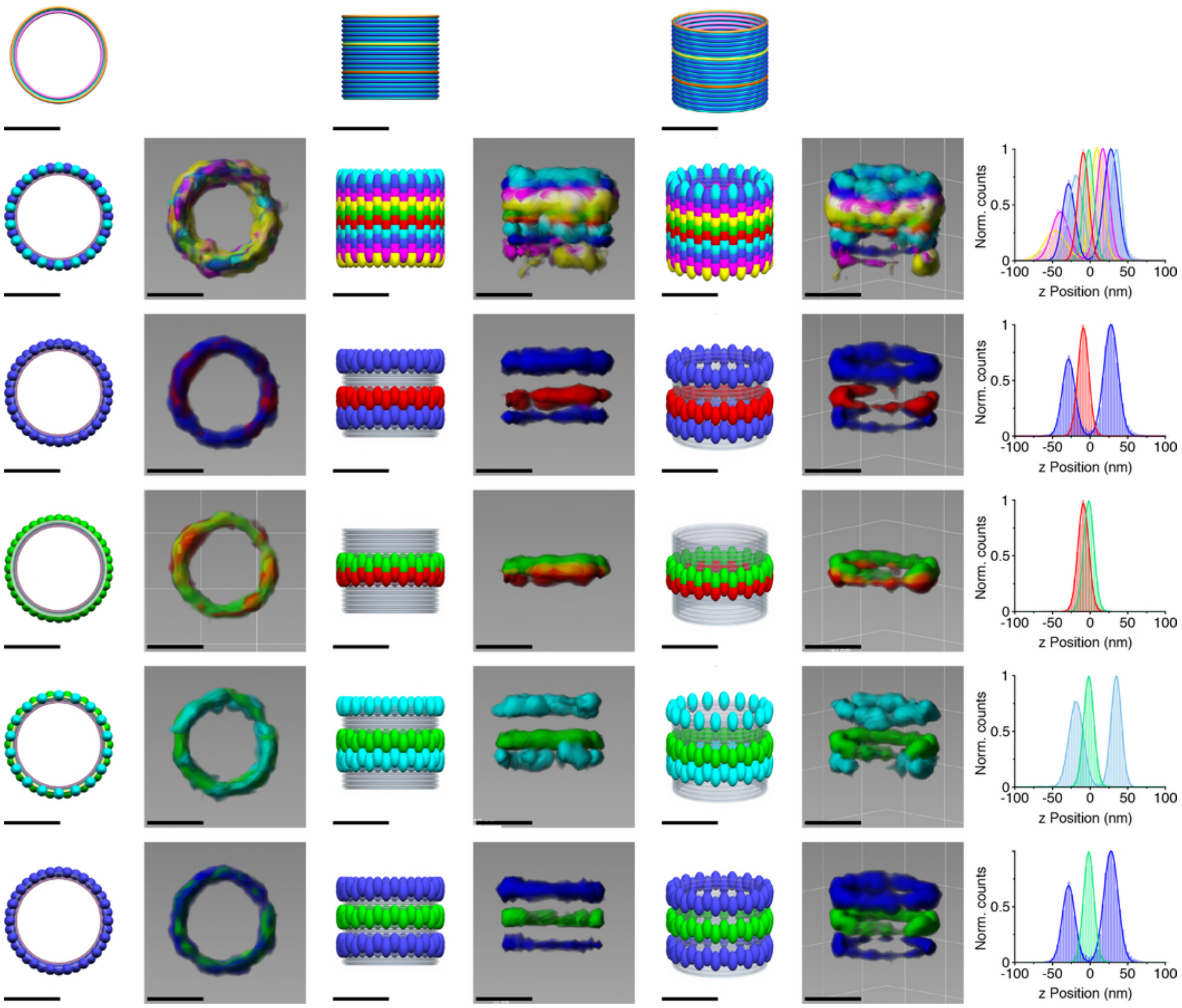

Supplementary Figure 70. DNA-PAINT data for 90-23 nm trimer (Supplement to Figure 3)

Scale bars 50 nm

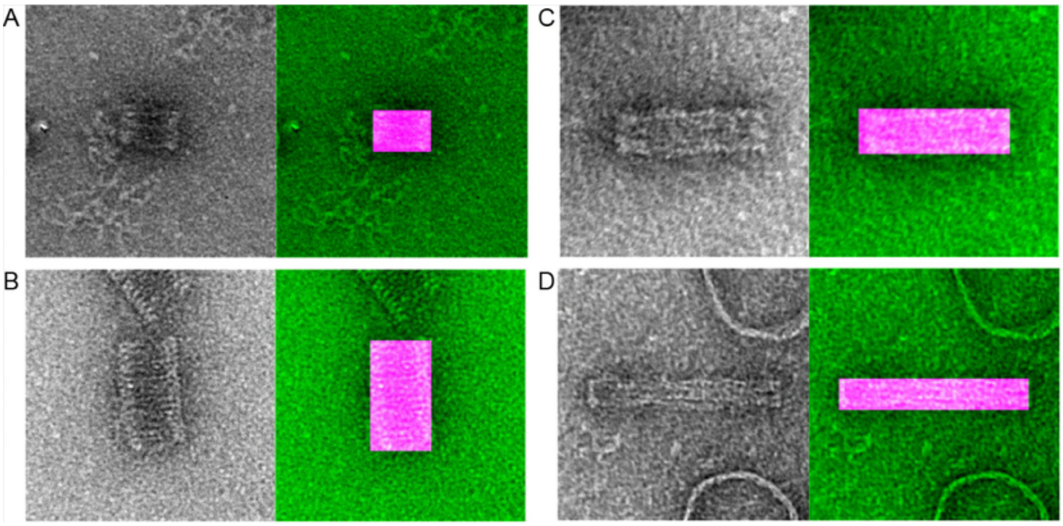

Supplementary Figure 71. Quantification of barrel height from TEM images

Side-by-side comparison of isolated DNA barrel images next to an overlay of the same image with a rectangular object with the highest 2D cross-correlation score with the barrel image. Top left - 30-27 nm barrel. Top right - 60-30 nm barrel. Bottom left - 30-65 nm barrel. Bottom right - 90-19 nm barrel.

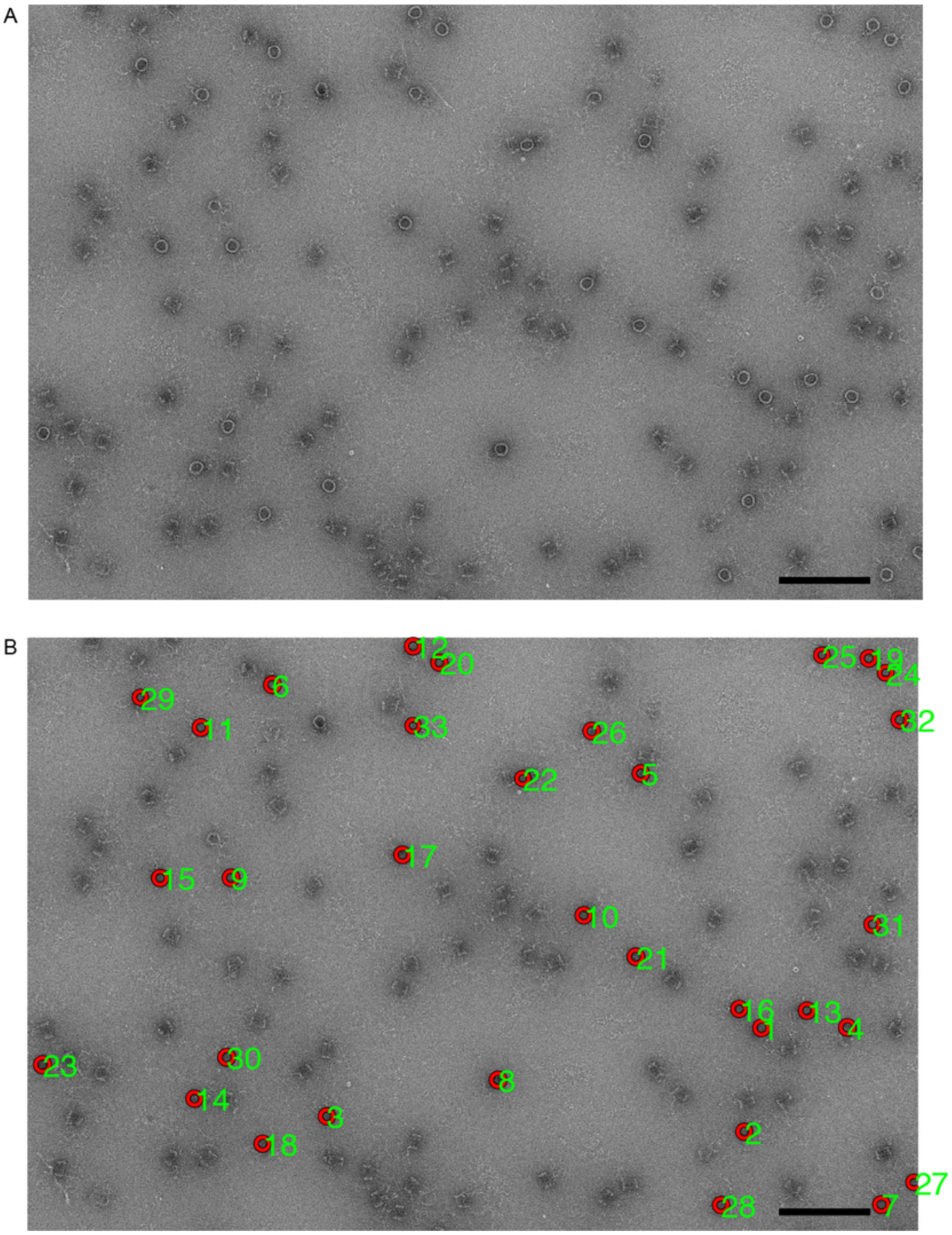

Supplementary Figure 72. Quantification of barrel diameter from TEM images

Example of automated identification of circular DNA barrel objects, in this case 30-27 nm barrel. Barrel diameters are estimated using best-fit parameters for a circular Hough transform to each object.

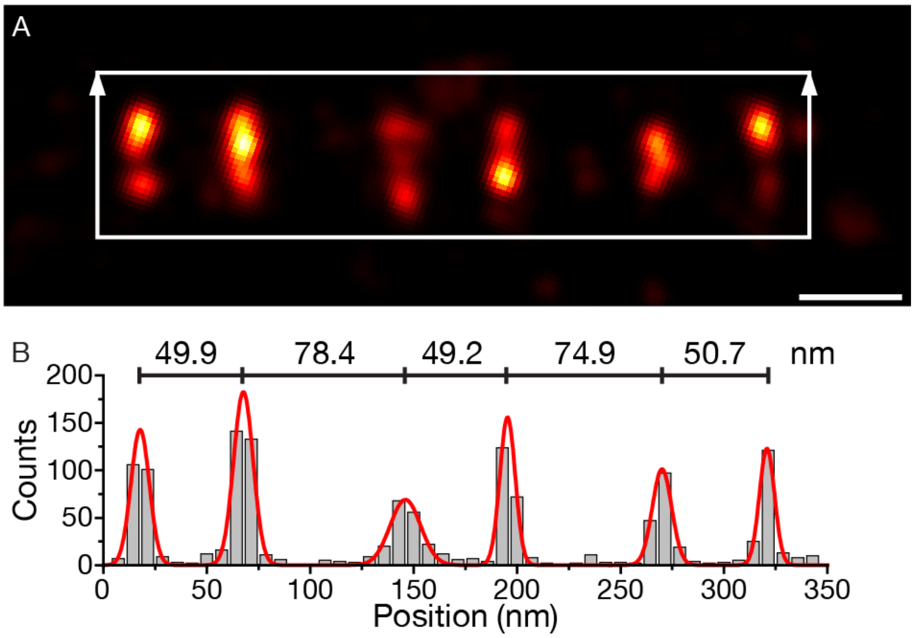

Supplementary Figure 73. Example quantification of barrel height from DNA-PAINT images DNA-PAINT measurement of height of 30-65 nm barrel polymer (Scale bar 50 nm). To quantify height of DNA barrel monomers by DNA-PAINT histograms were plotted along the long axis of polymers. Peaks were fitted to estimate the spacing between adjacent rings of docking sites on the barrel monomers, full results shown in Supplementary Table 5.

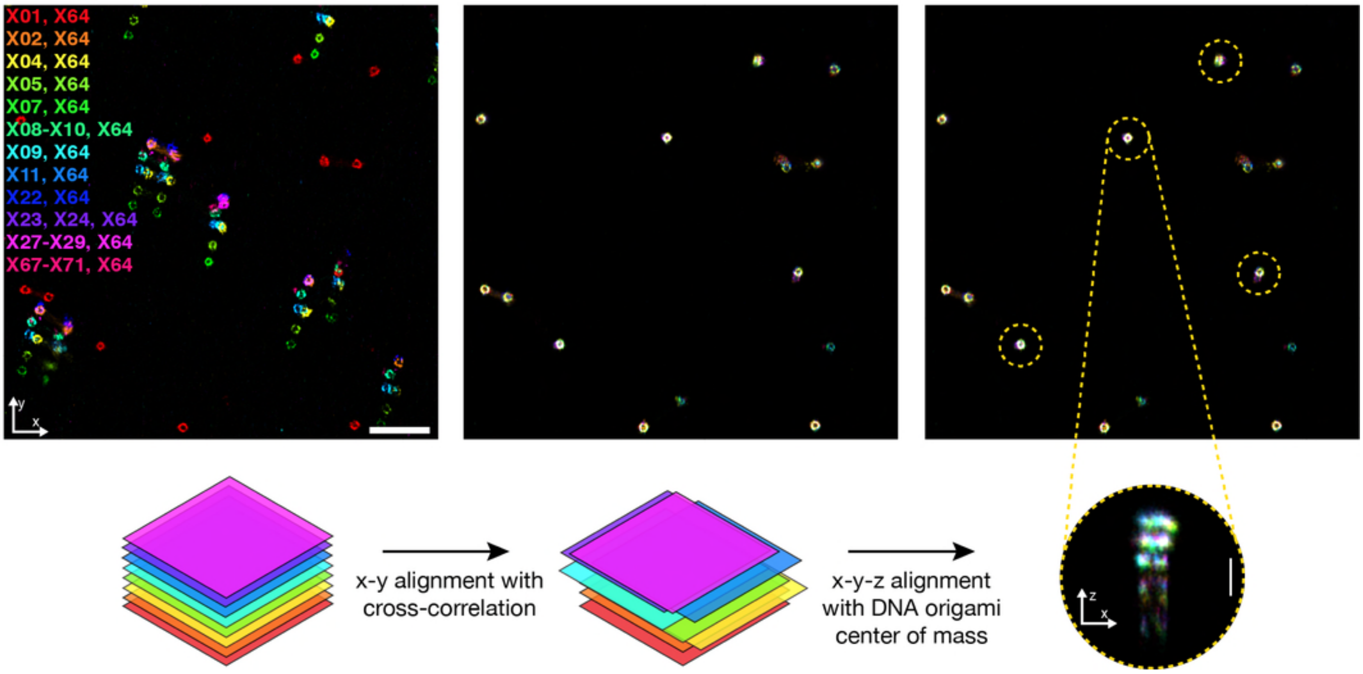

Supplementary Figure 74. Particle alignment process for DNA-PAINT images

Super-resolution DNA-PAINT reconstruction, drift correction, alignment and averaging was carried out using the software package Picasso, see methods for more detail. Scale 1 um (x-y axis), 100 nm (z axis).

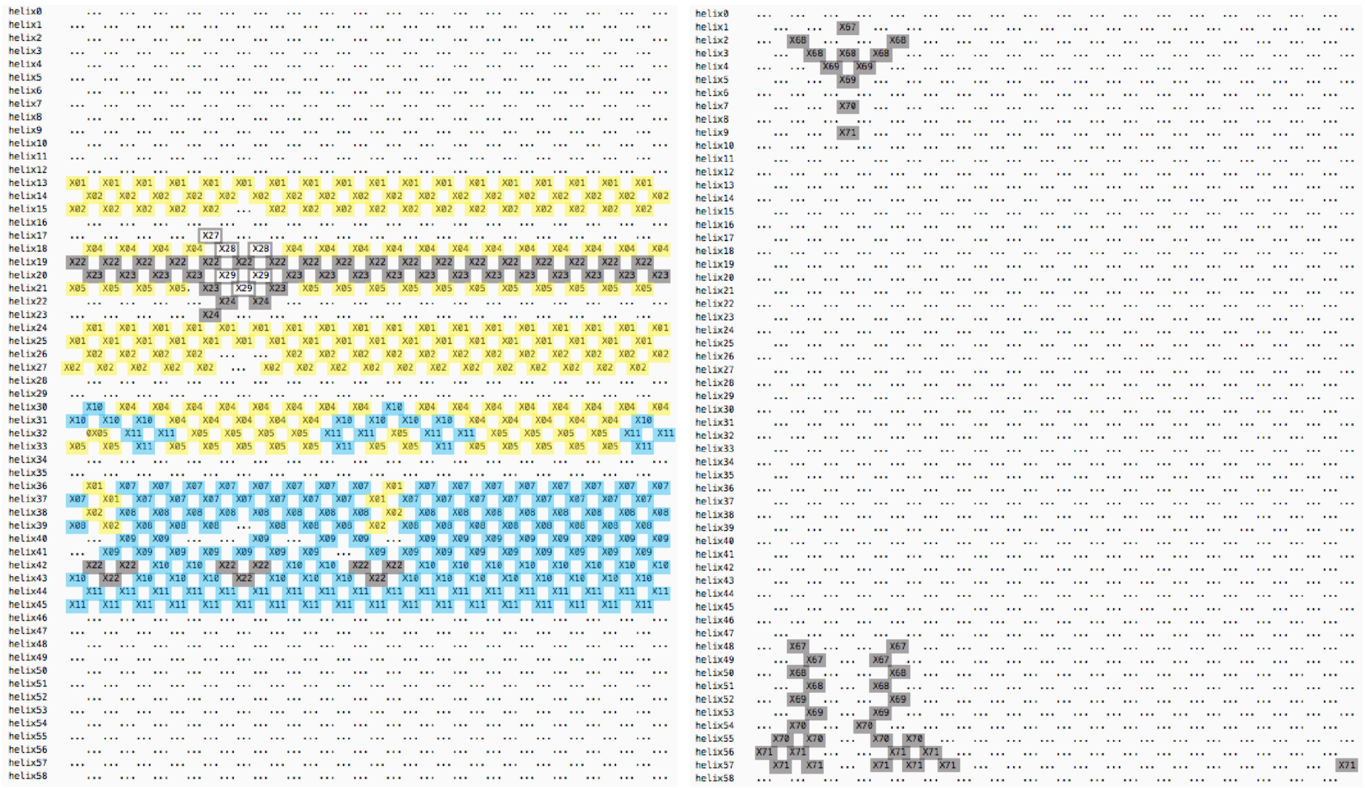

Supplementary Figure 75. DNA-PAINT docking site pattern locations for Figure 4

Pixel docking site identities for patterned 90 nm decamer in Figure 4, for outer (right) and inner (left) barrel surfaces. Docking site sequences are listed in Supplementary Table 9. Pixels are colored by their pseudocolor designation for visualisation in Figure 4.

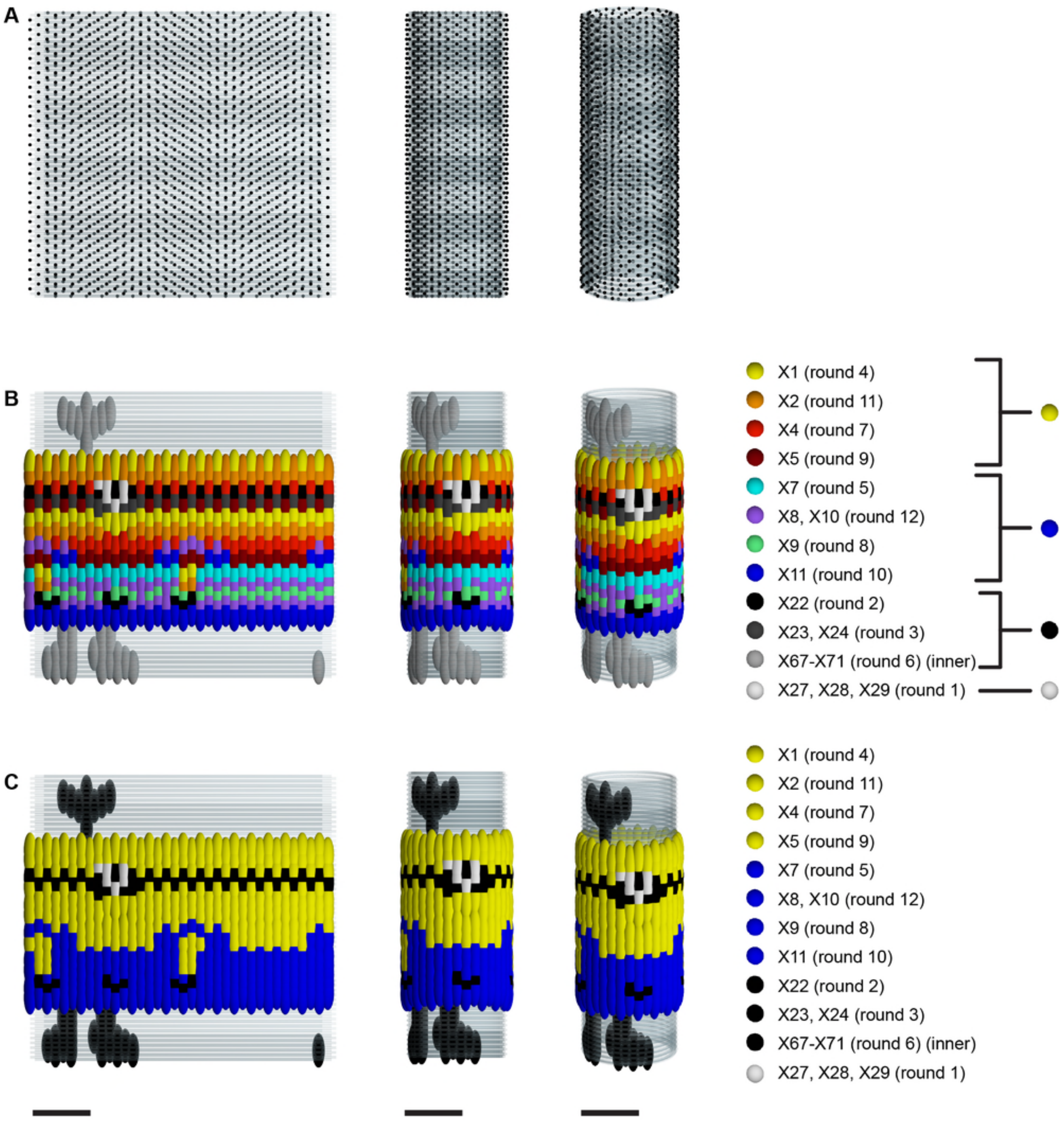

Supplementary Figure 76. 3D models for DNA-PAINT docking site pseudocolor allocation

A All docking site pixel locations for 90-23 nm decamer, **B** docking site pattern for decamer in Figure 4, coloured by PAINT imaging rounds (1-12), and **C** docking site pattern for decamer in Figure 4, coloured by pseudocolor. All models are shown as 'flattened' structure (left), orthographic view (middle), and perspective view (right). Docking site sequences are listed in Supplementary Table 9. In **B**, pixels colored scheme indicates equivalent pseudocolor designation for each imaging round, as used for visualisation in **C** and Figure 4. Pseudocolours are used to combine data from multiple Exchange-PAINT rounds for ease of 3D visualisation. Scale bars 50 nm.

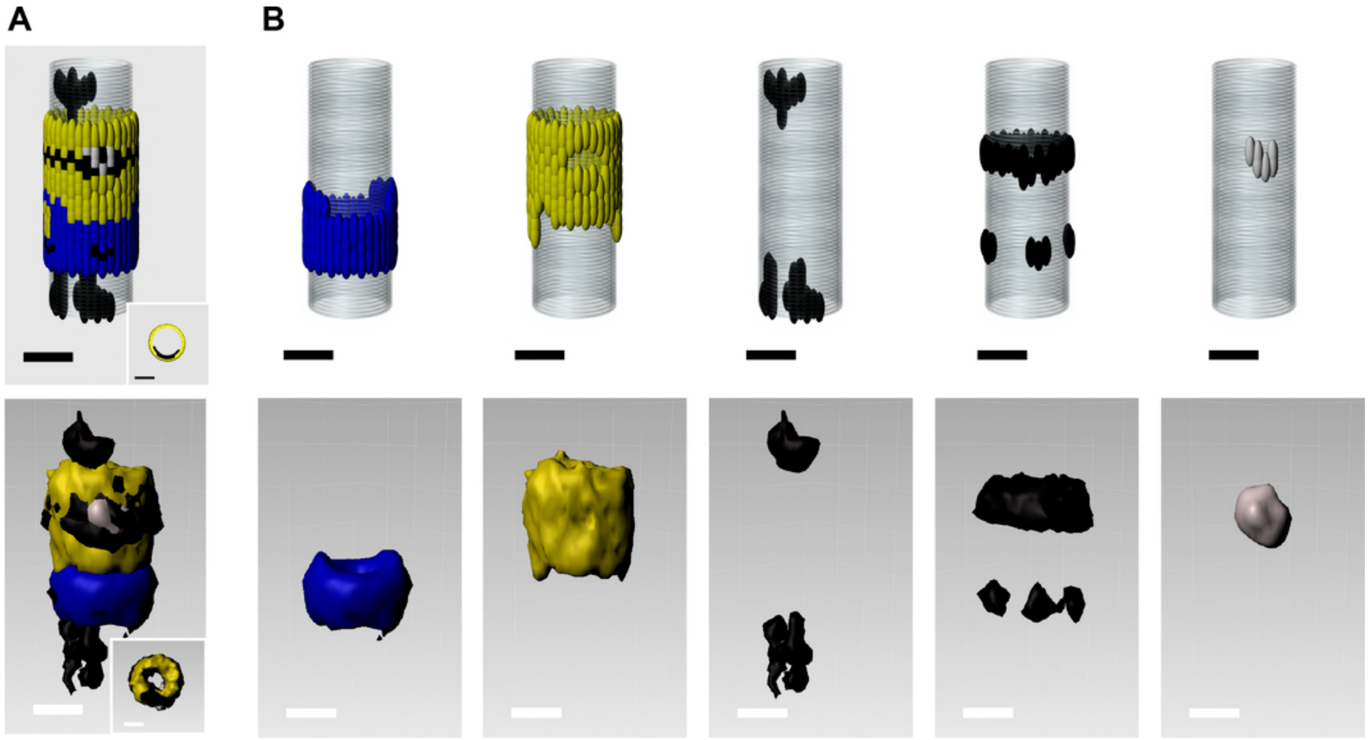

Supplementary Figure 77. DNA-PAINT decamer pattern, rendered as separate pseudocolors
A Patterned 90-23 nm decamer image (Fig 4D), **B** separately visualised as the 5 pseudocolor components corresponding to DNA-PAINT docking sequences, from left to right: outer 'blue' (X07, X08, X09, X10, X11), outer 'yellow' (X01, X02, X04, X05), inner 'black' (X67, X68, X69, X70, X71), outer 'black' (X22, X23, X24), and outer 'white' (X27, X28, X29). Top row shows design, bottom row shows DNA-PAINT data for summed particles. Scale bars 50 nm

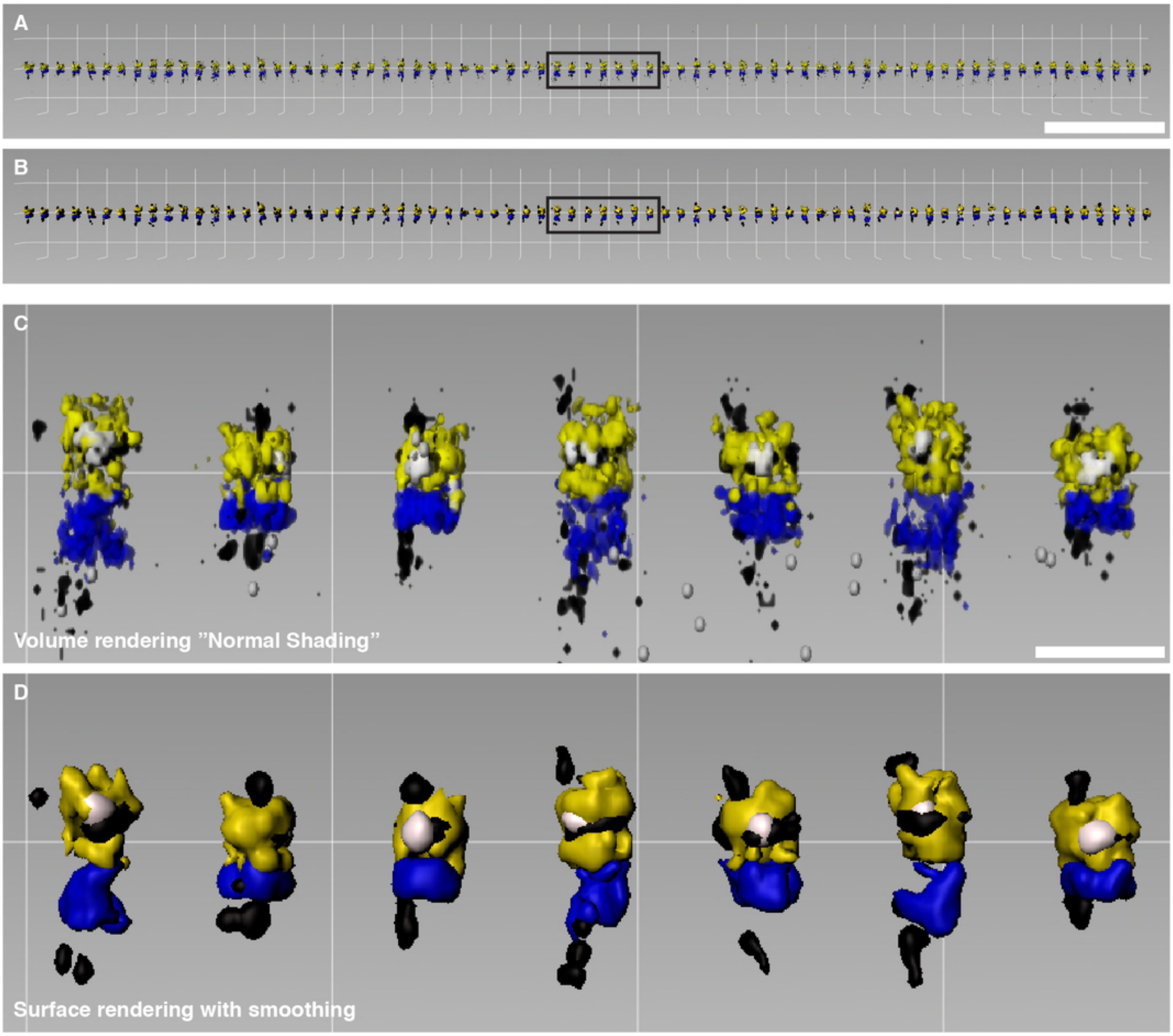

Supplementary Figure 78. DNA-PAINT decamer pattern, volume and surface rendering

For 3D super-resolution images, the data was imported as voxels to Bitplane Imaris 9 (Oxford Instruments) and rendered. In Figure 4, two different rendering options are used: volume rendering (Figure 4c,f) and surface rendering (Figure 4 d,e,f). Figure 4 e and f are shown here with both volume (a,c) and surface rendering (b,d) views. Scale 2 um (A,B), 200 nm (C,D)

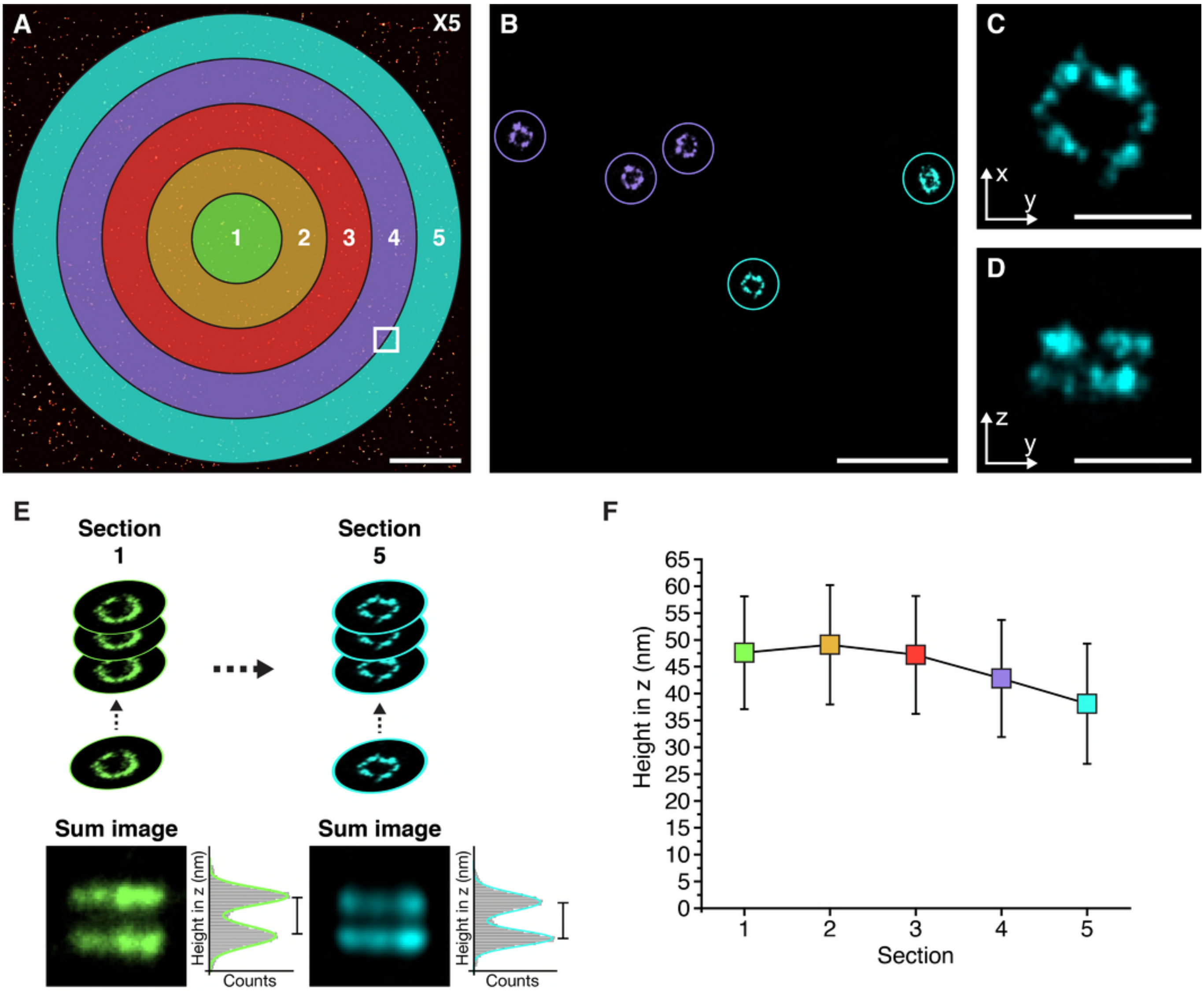

Supplementary Figure 79. DNA-PAINT z-height variation over field-of-view

Unexpected variations in the z-height of the 90 nm barrel decamers were observed in Figure 4 and Supplementary Figure 78. A single DNA-PAINT imager round ('X5') was used to analyse the apparent z-height variation across the DNA-PAINT field of view. For this imager sequence, the barrel pattern contains 2 rings with a designed spacing of 41.6 nm, and a predicted height from DNA-PAINT monomer measurements in Supplementary Table 5 of 44 nm. **A** The field of view was split into radial sections, 1-5, **B,C,D** decamers were selected for each region (N = 30, 99, 145, 213, 262 for sections 1-5 respectively), **E** particles were aligned and summed for each section, **F** ring spacing was plotted as a function of section. The apparent height was found to be correlated with the radial position of the particle in the field of view, indicating an imaging artefact, possibly due to the astigmatism lens used for 3D imaging.

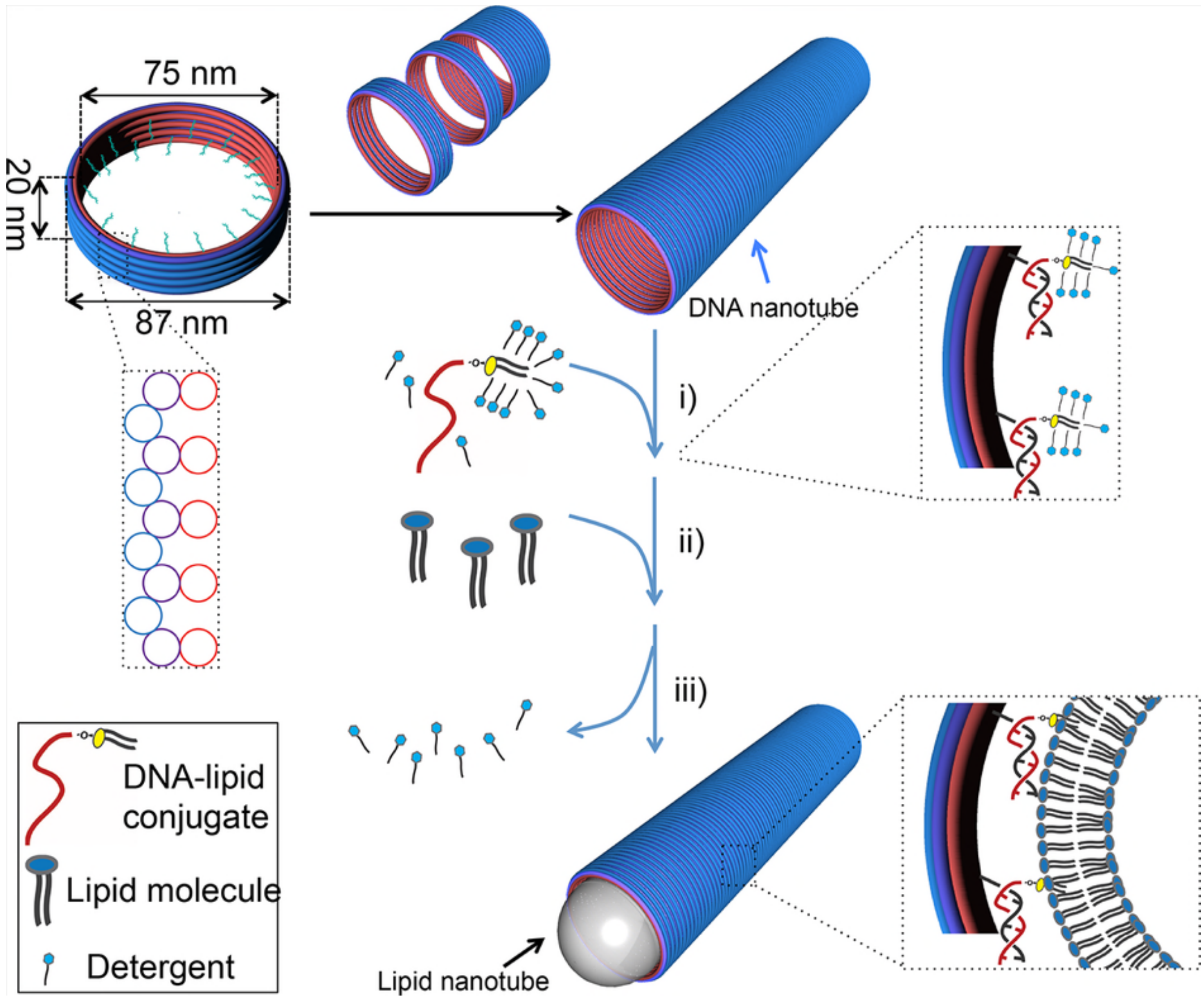

Supplementary Figure 80. Lipid functionalization of DNA-barrel polymers (Supplement to Figure 3) Schematic of DNA-nanotube-scaffolded lipid-nanotube reconstitution. Top, DNA nanotube formed through DNA barrel polymerization mediated by ssDNA hybridization. i) Saturation of DNA-nanotube handles (black) by lipid-DNA anti-handles (red) in the presence of detergent OG; ii) addition of free lipid molecules; iii) liposome reconstitution using dialysis to remove free detergent molecules.

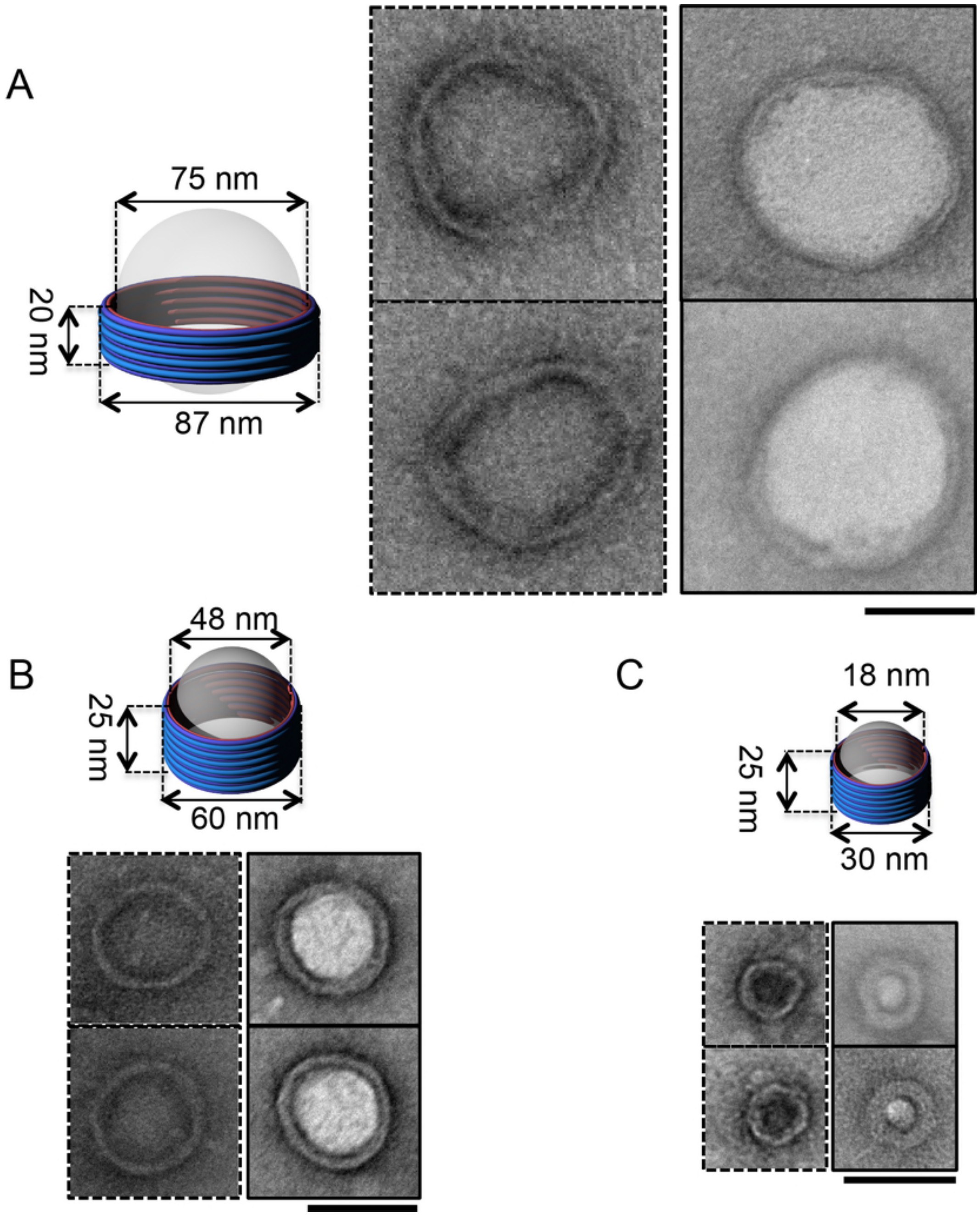

Supplementary Figure 81. DNA-barrel spherical-liposome reconstitution

Negative-stain TEM images of (A) 75-nm, (B) 48-nm, (C) 18-nm inner diameter DNA barrels before and after liposome reconstitution. (scale bar: 50 nm).

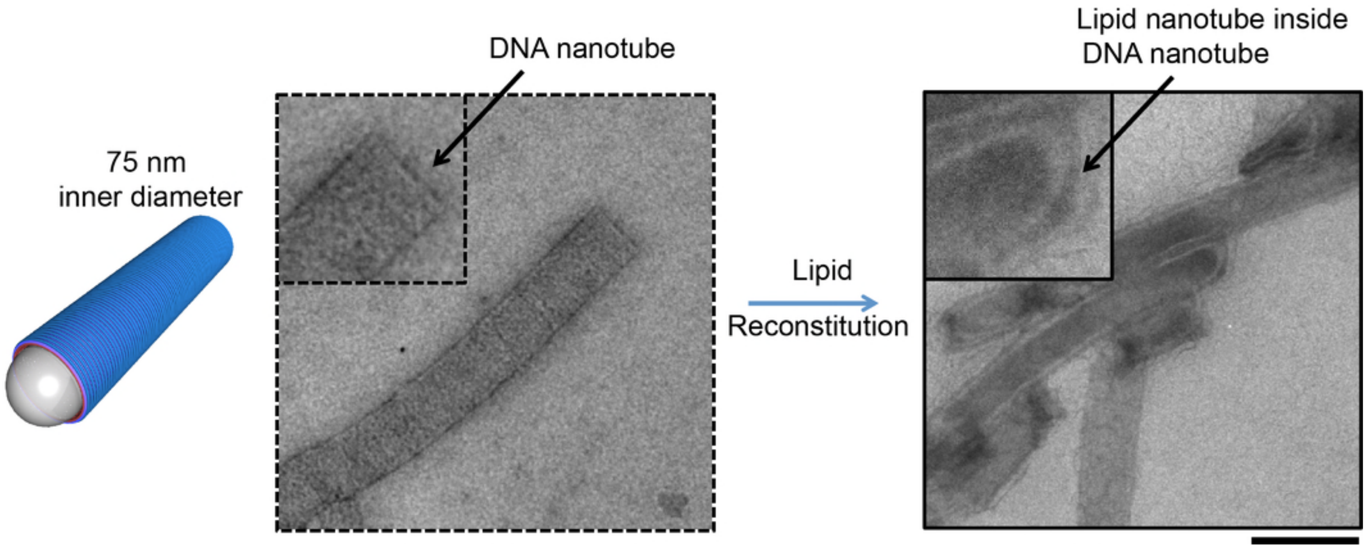

Supplementary Figure 82. DNA-barrel nanotube-liposome reconstitution
Negative-stain TEM images of DNA-barrel polymer ‘nanotube’, with 75 nm inner diameter, before (left) and after (right) scaffolded liposome reconstitution. (Scale bar: 100 nm).

Supplementary Tables

Supplementary Table 1. Monomer folding yields, quantified from agarose gel analysis

Structure	Type	Gel yield of monomer (%)
30-27 nm	Plain	80
	Connectors	54
	Pixels	86
	Connectors + Pixels	46
30-65 nm	Plain	77
	Connectors	64
	Pixels	73
	Connectors + Pixels	71
60-30 nm	Plain	85
	Connectors	73
	Pixels	82
	Connectors + Pixels	68
90-19 nm	Plain	68
	Connectors	64
	Pixels	73
	Connectors + Pixels	69
90-23 nm	Plain	80
	Connectors	73
	Pixels	76
	Connectors + Pixels	69

Supplementary Table 2. Purified 90-23 nm monomer yields, by monomer, by UV absorption

Design	Monomer	Purified yield (%)
Plain<br>0 outer pixels<br>0 inner pixels<br>0 pixels total	m0	50.5
	m1	53
	m2	55
	m3	46.5
	m4	51
	m5	50
	m6	50.5
	m7	45
	m8	48
	m9	51
All pixels<br>1062 outer pixels<br>1062 inner pixels<br>2124 pixels total	m0	13.5
	m1	28.5
	m2	28.5
	m3	27.5
	m4	15.5
	m5	23
	m6	34
	m7	22
	m8	39
	m9	11
Pixel design 1 (Fig 4)<br>612 outer pixels<br>152 inner pixels<br>764 pixels total	m0	57
	m1	43
	m2	28.5
	m3	47
	m4	48
	m5	46
	m6	42
	m7	232.5
	m8	50.5
	m9	48

Supplementary Table 3. Purified 90-23 nm monomer yields, averaged m0-m9, by UV absorption

Design	Number of pixels	Average purified monomer yield (%)
Plain	0	50.1
All outer + inner	1062 outer + 1062 inner = 2124	23.2
All outer	1062 outer	34.6
All inner	1062 inner	40.4
Pattern 1 (Fig 4)	612 outer + 152 inner = 764	42.5

Supplementary Table 4. Final purified 90-23 nm decamer yields, by UV absorption

Design	Number of pixels	Final purified 10mer yield (%)
Plain	0	5
All outer + inner	1062 outer + 1062 inner = 2124	0.7
All outer	1062 outer	0.7
All inner	1062 inner	1
Pattern 1 (Fig 4)	612 outer + 152 inner = 764	0.5
Pattern 2	575 outer + 576 inner = 1151	2
Pattern 3	412 outer + 236 inner = 648	1.8

Supplementary Table 5. Barrel dimensions from TEM and DNA-PAINT Quantification

Height

Structure	Height - Design	Height – TEM sideway, monomers (n = 5)	Height - PAINT sideways, polymers	Height - PAINT upright, polymers
30-27 nm	29 nm	21 ± 1 nm	-	-
30-65 nm	62 nm	59 ± 1 nm	63 ± 1.8 nm (n = 77)	-
60-30 nm	29 nm	26 ± 2 nm	33 ± 2.8 nm (n = 77)	-
60-32 nm	33 nm	28 ± 3 nm	-	-
90-19 nm	21nm	20 ± 1 nm	22 ± 1.5 nm (n = 60)	-
90-23 nm	25 nm	25 ± 1 nm	-	*28 ± 7 nm (3mer)*

Diameter

structure	Diameter - Design	Diameter - TEM upright	Diameter - PAINT upright	Diameter – TEM sideways, monomer (n = 5)
30-27 nm	31 nm	29 ± 1 nm (n=85)		32 ± 1 nm (~10% distortion)
30-65 nm	31 nm	29 ± 1 nm (n=52)	31 ± 3.8 nm (n=726)	33 ± 1 nm (~10%)
60-30 nm	60 nm	57 ± 3 nm (n=49)	60 ± 4.1 nm (n =16)	81 ± 2 nm (~35%)
90-19/23 nm	87 nm	87 ± 3 nm (n=43)	85 ± 6.1 nm (n=27)	123 ± 4 nm (~50%)
90-23 nm 10mer	87 nm			124 ± 7 nm (~50%)

Supplementary Table 6. Experimental conditions for super-resolution imaging in Figure 2

Microscope setting	Condition
**Microscope**	Setup 1
**Objective**	Apo SR TIRF 100X Oil
**Camera**	Andor Zyla 4.2
**Field of view**	512×512 pixel after binning
**Exposure time**	200 ms
**Binning**	2×2
**Tube lens**	1×
**Excitation laser**	561 nm [max power 200 mW]

Sample settings	Condition
**Sample target**	30 nm DNA barrel monomer
**Imager sequence**	P10
**Imager concentration**	3 nM
**Imaging buffer**	B with PCA/PCD/TX
**Dye**	Cy3B
**Frames**	50 000
**Laser power**	50 mW
**NeNA localization precision**	2.0 nm

Sample settings	Condition
**Sample target**	60 nm DNA barrel monomer
**Imager sequence**	P1
**Imager concentration**	2.5 nM
**Imaging buffer**	B with PCA/PCD/TX
**Dye**	Cy3B
**Frames**	15 000
**Laser power**	150 mW
**NeNA localization precision**	2.7 nm

Sample settings	Condition
**Sample target**	90 nm DNA barrel monomer
**Imager sequence**	X1
**Imager concentration**	800 pM
**Imaging buffer**	B with PCA/PCD/TX
**Dye**	Cy3B
**Frames**	25 000
**Laser power**	200 mW
**NeNA localization precision**	3.0 nm

Supplementary Table 7. Experimental conditions for super-resolution imaging in Figure 3

Microscope setting	Condition
**Microscope**	Setup B
**Objective**	Apo TIRF 100X Oil
**Camera**	Andor Zyla 4.2
**Field of view**	512×512 pixel after binning
**Exposure time**	200 ms
**Binning**	2×2
**Tube lens**	1×
**Excitation laser**	561 nm [max power 300 mW]

Sample settings	Condition
**Sample target**	30 nm DNA barrel polymer
**Imager sequence**	X1, P1
**Imager concentration**	1 nM, 1 nM
**Imaging buffer**	B with PCA/PCD/TX
**Dye**	Cy3B
**Frames**	50 000
**Laser power**	50 mW
**NeNA localization precision**	2.6 nm

Sample settings	Condition
**Sample target**	60 nm DNA barrel polymer
**Imager sequence**	X1, P1
**Imager concentration**	500 pM, 2 nM
**Imaging buffer**	B with PCA/PCD/TX
**Dye**	Cy3B
**Frames**	50 000
**Laser power**	35 mW
**NeNA localization precision**	3.0 nm

Sample settings	Condition
**Sample target**	90 nm DNA barrel polymer
**Imager sequence**	X2, P1
**Imager concentration**	200 pM, 2 nM
**Imaging buffer**	B with PCA/PCD/TX
**Dye**	Cy3B
**Frames**	30 000
**Laser power**	40 mW
**NeNA localization precision**	2.3 nm

Supplementary Table 8. Experimental conditions for super-resolution imaging in Figure 4

Microscope setting	Condition
**Microscope**	Setup B
**Objective**	Apo SR TIRF 100X Oil
**Camera**	Andor Zyla 4.2
**Field of view**	512×512 pixel after binning
**Exposure time**	200 ms
**Binning**	2×2
**Tube lens**	1×
**Excitation laser**	561 nm [max power 200 mW]

Sample settings	Condition
**Sample target**	90 nm DNA barrels 10mers
**Imaging buffer**	B with PCA/PCD/TX
**Dye**	Cy3B
**Frames**	25 000 each round
**Laser power**	100 mW
**Round 1: Eye**	**X27**: 4 nM, **X28**: 2 nM , **X29**: 1.5 nM & X64: 100 pM<br>NeNA localization precision: 6.3 nm
**Round 2: Eye band**	**X22**: 4 nM & X64: 100 pM<br>NeNA localization precision: 4.0 nm
**Round 3: Eye band**	**X23**: 500 pM, **X24**: 3.5 nM & X64: 100 pM<br>NeNA localization precision: 4.5 nm
**Round 4: Torso**	**X1**: 2 nM & X64: 100 pM<br>NeNA localization precision: 4.7 nm
**Round 5: Pants**	**X7**: 3 nM & X64: 100 pM<br>NeNA localization precision: 4.0 nm
**Round 6: Hair and feet**	**X67**: 900 pM, **X68**: 500 pM, **X69**: 600 pM, **X70**: 600 pM, **X71**: 400 pM & X64: 100 pM<br>NeNA localization precision: 4.4 nm
**Round 7: Torso**	**X4**: 4 nM & X64: 100 pM<br>NeNA localization precision: 4.2 nm
**Round 8: Pants**	**X9**: 3 nM & X64: 100 pM<br>NeNA localization precision: 3.9 nm
**Round 9: Torso**	**X5**: 3 nM & X64: 100 pM<br>NeNA localization precision: 4.4 nm
**Round 10: Pants**	**X11**: 2.5 nM & X64: 100 pM<br>NeNA localization precision: 3.6 nm
**Round 11: Torso**	**X2**: 2 nM & X64: 100 pM<br>NeNA localization precision: 4.3 nm
**Round 12: Pants**	**X8**: 1 nM, **X10**: 1 nM & X64: 100 pM<br>NeNA localization precision: 3.7 nm

Supplementary Table 9. DNA-PAINT docking site sequences

Name	Sequence	Modification
**P1 docking sequence**	TT-ATACATCTA	-
**X1 docking sequence**	TT-AATTTACCTC	-
**X2 docking sequence**	TT-ACCTTTACTA	-
**X4 docking sequence**	TT-TTCTCCAATA	-
**X5 docking sequence**	TT-ATCTTCATAC	-
**X7 docking sequence**	TT-ACTTTAATCC	-
**X8 docking sequence**	TT-AATTCCTCTA	-
**X9 docking sequence**	TT-AATTACTTCC	-
**X10 docking sequence**	TT-ACACCTTATT	-
**X11 docking sequence**	TT-ATATTTCACC	-
**X22 docking sequence**	TT-TCATCTAACT	-
**X23 docking sequence**	TT-ACACATCTTT	-
**X24 docking sequence**	TT-TTAACTCTCA	-
**X27 docking sequence**	TT-ATCATACCTT	-
**X28 docking sequence**	TT-ATCATTCTAC	-
**X29 docking sequence**	TT-TCCATACATT	-
**X64 docking sequence**	TT-TCTTATACAC	-
**X67 docking sequence**	TT-ACTCTATTCA	-
**X68 docking sequence**	TT-ATCAATCTTC	-
**X69 docking sequence**	TT-TTTCTAAACC	-
**X70 docking sequence**	TT-TCAATATCTC	-
**X71 docking sequence**	TT-ATTCTATCCA	-

Supplementary Table 10. DNA-PAINT imager sequences

Name	Sequence	Modification
**P1 imager sequence**	CTAGATGTAT	3' – Cy3B
**X1 imager sequence**	GAGGTAAATT	3' – Cy3B
**X2 imager sequence**	TAGTAAAGGT	3' – Cy3B
**X4 imager sequence**	TATTGGAGAA	3' – Cy3B
**X5 imager sequence**	GTATGAAGAT	3' – Cy3B
**X7 imager sequence**	GGATTAAAGT	3' – Cy3B
**X8 imager sequence**	TAGAGGAATT	3' – Cy3B
**X9 imager sequence**	GGAAGTAATT	3' – Cy3B
**X10 imager sequence**	AATAAGGTGT	3' – Cy3B
**X11 imager sequence**	GGTGAAATAT	3' – Cy3B
**X22 imager sequence**	AGTTAGATGA	3' – Cy3B
**X23 imager sequence**	AAAGATGTGT	3' – Cy3B
**X24 imager sequence**	TGAGAGTTAA	3' – Cy3B
**X27 imager sequence**	AAGGTATGAT	3' – Cy3B
**X28 imager sequence**	GTAGAATGAT	3' – Cy3B
**X29 imager sequence**	AATGTATGGA	3' – Cy3B
**X64 imager sequence**	GTGTATAAGA	3' – Cy3B
**X67 imager sequence**	TGAATAGAGT	3' – Cy3B
**X68 imager sequence**	GAAGATTGAT	3' – Cy3B
**X69 imager sequence**	GGTTTAGAAA	3' – Cy3B
**X70 imager sequence**	GAGATATTGA	3' – Cy3B
**X71 imager sequence**	TGGATAGAAT	3' – Cy3B
