# Supplementary material for: Complex multicomponent patterns rendered on a 3D DNA-barrel pegboard
Source: Nat Commun. 2020 Nov 13;11:5768. doi: 10.1038/s41467-020-18910-x (PMC7666213; doi:10.1038/s41467-020-18910-x)
Supplement: Supplementary file 18 — Description of Additional Supplementary Files [file 41467_2020_18910_MOESM18_ESM.pdf]

Title: Supplementary Movie 1
Description: Design of pixel pattern programmed on decamer 90 nm barrel as in Figure 4a–d.

Title: Supplementary Movie 2
Description: Rotating view of composite sum Exchange-PAINT image with surface rendering as in Figure 4f.

Title: Supplementary Movie 3
Description: Rotating view of Exchange-PAINT image of a field of upright patterned particles alongside a few registration particles, with volume rendering as in Figure 4e.

Title: Supplementary Movie 4
Description: Scanning view of Exchange-PAINT image of selected particles with volume rendering, computationally lined up as a panel as in Figure 4g.

Title: Supplementary Movie 5
Description: Rotating view of Exchange-PAINT image of select particle with volume rendering, as in Figure 4j.

Title: Supplementary Movie 6
Description: Rotating view of Exchange-PAINT image of a select pair of particles with volume rendering, as in Figure 4i.

Title: Supplementary Data 1
Description: 30-27nm barrel DNA sequences in csv format

Title: Supplementary Data 2
Description: 30-65nm barrel DNA sequences in csv format

Title: Supplementary Data 3
Description: 30-61nm barrel DNA sequences in csv format

Title: Supplementary Data 4
Description: 60-30nm barrel DNA sequences in csv format

Title: Supplementary Data 5
Description: 60-32nm barrel DNA sequences in csv format

Title: Supplementary Data 6
Description: 90-19nm barrel DNA sequences in csv format

Title: Supplementary Data 7
Description: 90-23nm barrel DNA sequences in csv format

Title: Supplementary Data 8
Description: 120-15nm barrel DNA sequences in csv format
